# FNDC5 is produced in the stomach and associated to body composition

**DOI:** 10.1038/srep23067

**Published:** 2016-03-10

**Authors:** S. Barja-Fernández, C. Folgueira, C. Castelao, O. Al-Massadi, S. B. Bravo, T. Garcia-Caballero, R. Leis, M. Pardo, F. F. Casanueva, L. M. Seoane

**Affiliations:** 1 Grupo Fisiopatología Endocrina, Instituto de Investigación Sanitaria de Santiago de Compostela (IDIS), Complexo Hospitalario Universitario de Santiago (CHUS/SERGAS). Santiago de Compostela, Spain; 2 Departamento de Pediatría, Universidade de Santiago de Compostela, Instituto de Investigación Sanitaria de Santiago de Compostela (IDIS), Complexo Hospitalario Universitario de Santiago (CHUS/SERGAS). Santiago de Compostela, Spain; 3 CIBER Fisiopatología Obesidad y Nutrición, Instituto de Salud Carlos III, Santiago de Compostela, Spain; 4 Department of Physiology, Research Centre of Molecular Medicine and Chronic Diseases (CIMUS), Universidade de Santiago de Compostela, Instituto de Investigación Sanitaria de Santiago de Compostela (IDIS). Santiago de Compostela, Spain; 5 Unidad de Proteómica, Instituto de Investigación Sanitaria de Santiago de Compostela (IDIS), Complexo Hospitalario Universitario de Santiago CHUS/SERGAS. Santiago de Compostela, Spain; 6 Departamento de Ciencias Morfológicas, Facultad de Medicina, Universidade de Santiago de Compostela. Complexo Hospitalario Universitario de Santiago (CHUS/SERGAS). Santiago de Compostela, Spain; 7 Grupo Obesidomica, Instituto de Investigación Sanitaria de Santiago de Compostela (IDIS), Complexo Hospitalario Universitario de Santiago (CHUS/SERGAS). Santiago de Compostela, Spain; 8 Laboratorio de Endocrinología Molecular y Celular, Instituto de Investigación Sanitaria de Santiago de Compostela (IDIS), Complexo Hospitalario Universitario de Santiago (CHUS/SERGAS), Santiago de Compostela, Spain

## Abstract

The fibronectin type III domain-containing protein 5 (FNDC5) discovered in 2002 has recently gained attention due to its potential role in protecting against obesity. In rat, no data exist regarding FNDC5 production and regulation in the stomach. The aim of the present work was to determine the expression of FNDC5 in the rat stomach and its potential regulation by body composition. The present data shows FNDC5 gene expression in the gastric mucosa. Immunohistochemical studies found FNDC5 immunopositivity in chief cells of gastric tissue. By the use of three different antibodies FNDC5 was found expressed in gastric mucosa and secreted by the stomach. The rate of gastric FNDC5 secretion parallels the circulating levels of FNDC5. The body fat mass increase after intervention with high fat diet coincided with a decrease in the secretion of FNDC5 from the stomach and a diminution in the FNDC5 circulating levels. In summary, the present data shows, for the first time, the expression of FNDC5 in the stomach of rats and its regulation by body composition, suggesting a potential role of gastric FNDC5 in energy homeostasis.

Obesity represents a major public health problem in developed countries. Currently, the most effective treatment for this pathology is gastric surgery^1^,^2^. This finding suggests that signals from the gastrointestinal tract are crucial for the regulation of energy balance^3^,^4^,^5^. Accordingly, the stomach plays a key role in the homeostatic mechanism that is involved in the control of energy homeostasis, and therefore, gastrointestinal-derived peptides have been revealed to be one of the most promising targets in treating obesity^6^.

Fibronectin type III domain-containing protein 5 (FNDC5), also called FRCP2 and Pep, was first discovered and characterized in 2002 by two independent groups^7^,^8^. FNDC5 mRNA was identified in several tissues, such as the heart, brain, ovary, testis, kidney and liver, among others^9^. FNDC5 has recently received great attention due to the identification of a novel peptide in muscle^10^,^11^ and adipose tissue^12^, named irisin, which has been proposed to be a soluble product of FNDC5 by cleavage at the C-terminal region (at amino acid position 30 and 140) by an unknown protease. Böstrom and colleagues reported increased FNDC5 mRNA levels in the skeletal muscle of mice and humans after exercise^10^. Moreover, a potential role of irisin in protecting against obesity and associated disorders was proposed based on the fact that forced FNDC5 overexpression in both lean and diet-induced obese mice provoked the browning of white adipose tissue (WAT). In addition, a moderate increase in the circulating irisin levels was shown to increase energy expenditure, to reduce body weight gain and to improve insulin resistance induced by a high fat diet. FNDC5 gene expression has been described to be regulated by peroxisome proliferator-activated receptor-γ coactivator-1 alpha (PGC1α), and it has been proposed to induce the browning of subcutaneous adipocytes and thermogenesis by increasing uncoupling protein 1 (UCP1) levels, both in animal models and cell cultures^9^.

Controversial data were recorded in the literature on the correlation between serum/plasma irisin and BMI. Some authors reported that irisin was positively correlated with BMI^9^,^13^,^14^,^15^,^16^,^17^. However, others reported an inverse relationship between circulating irisin levels and obesity^18^,^19^ or no correlation with BMI^20^.

Interestingly, it was shown that irisin levels dropped after six months post-surgery in obese patients who had undergone bariatric surgery^9^. This finding might suggest that in humans the FNDC5/irisin produced by the stomach would contribute to the circulating irisin levels. On the other hand, a more recent study proposes that bariatric surgery does not affect FNDC5/irisin levels^21^. Anyhow, gastric FNDC5/irisin production in rat stomach has not yet been assayed.

Taking into account the relevant role of the gastrointestinal tract in energy homeostasis, the hypothesis of the present study is based in the potential expression of FNDC5 in gastric mucosa as a component of the stomach-adipose tissue axis to regulate body composition in rat.

In this context, the main objective of the present work was to determine the expression of FNDC5 in gastric mucosa and its potential regulation by body composition.

## Materials and Methods

### Ethics Statement

The authors of this manuscript declare that all of the procedures carried out with animal models in this study were performed under 15005AE/10/FUN01/FIS02/LSC1 according to the institutional guidelines and the European Union standards for the care and use of experimental animals (Real Decreto 1201/2005, October 10th, regarding the animals used for the protection of research animals). The procedures were approved by Conselleria de Medio Rural, Government of Galicia and the Animal Care Committee of Santiago de Compostela University (Santiago de Compostela, Spain). Animal experimentation was designed by LM Seoane, with diploma type C, which was expedited by Conselleria de Medio Rural, Government of Galicia, Spain.

### Animal models and experimental designs

Male Sprague-Dawley rats were used. Rats were housed in a temperature-controlled environment of 22 °C and on a 12:12-h light-dark cycle (light on 08:00 to 20:00 hours).

To cause diet-induced obesity, after weaning the animals were fed with free access to a high fat diet (HF; n = 10) purchased from Research Diets, Inc. USA (4.73 kcal/g; composed of 45% of energy as lipids, 35% of energy as carbohydrate and 20% of energy as protein). As a control, an additional group was fed ad libitum with a standard laboratory diet (C; n = 10) (3.85 kcal/g; composed of 10% of energy as lipids, 70% of energy as carbohydrate and 20% of energy as protein; Research Diets, Inc. USA). Both experimental groups were maintained on these diets ad libitum until they reached adulthood (for 13 weeks).

The animals were euthanatized at 16 weeks of age by decapitation. Upon euthanasia, trunk blood was collected and the stomach, adipose tissue (visceral, subcutaneous and interscapular brown adipose tissue), and muscle (skeletal muscle) were excised. Gastric tissue explants obtained from the experimental model were incubated as described below. Trunk blood was immediately centrifuged, and plasma was stored at −80 °C for the biochemical measurements.

### Biometric parameters and food intake

Body weight and food consumption were monitored weekly. Body composition (fat mass) was assessed using nuclear magnetic resonance (Echo-MRI, Houston, USA).

### Experimental techniques

#### Tissue explant culture

Tissue explants were obtained from adult Sprague-Dawley rats^3^,^4^. After euthanasia, the stomach was rapidly excised and transported to the lab in a sterile Krebs-Ringer-HEPES buffer (NaCl, 125 mmol/l; KCl, 5 mmol/l; MgSO4, 1.2 mmol/l; KH2PO4, 1.3 mmol/l; CaCL2, 2 mmol/l; glucose, 6 mmol/l; HEPES 25 mmol/l; pH 7.4) with penicillin (100 U/ml) and streptomycin (100 μg/ml). After removing the blood vessels and connective tissue, the stomach tissue was washed with sterile Krebs-Ringer-HEPES buffer. The tissue explants were placed in six-well dishes containing 2.5 ml of Dulbecco´s modified Eagle´s medium supplemented with L-glutamine (200 mmol/l), penicillin (1000 U/ml) and streptomycin (100 μg/ml). After a pre-incubation period of 1 hour at 37 °C under a humidified atmosphere of 95% air-5% CO2, the media was aspirated and 2.5 ml of fresh medium was dispensed into each well. The culture medium was then collected at 2 hours, and the tissue explants were weighed. The gastric secretomes were processed for sample concentration by ultracentrifugation units (Amicon Ultra 3-kDa cut off, Millipore, Billerica, USA).

#### Protein extraction and western blotting analyses

Whole tissue proteins were prepared by homogenization using a TissueLyser II (Qiagen, Tokyo, Japan) in cold RIPA buffer [containing 200 mmol/l Tris/HCl (pH 7.4), 130 mmol/l NaCl, 10%(v/v) glycerol, 0.1%(v/v) SDS, 1%(v/v) Triton X-100, 10 mmol/l MgCl2] with anti-proteases and anti-phosphatases (Sigma-Aldrich; St. Louis, MO). The tissue lysates were centrifuged for 10 minutes (gastric mucosa) or 30 minutes (adipose tissue and muscle) at 18,000 g in a microfuge at 4 °C. Then, 30 μg of stomach mucosa, adipose tissue (white and brown adipose tissue) and muscle, as well as 25 μg of stomach tissue secreted proteins (secretome) and 2 μl of plasma were separated in 15% (FNDC5 and irisin) or 10% (PGC1α) sodium–dodecyl sulfate-polyacrylamide gels (SDS–PAGE) and electroblotted onto nitrocellulose membranes (catalog 162–0115, Bio-Rad Laboratories, Inc. Gernany). Equal loading of the gels and proper transfer of the proteins to the membrane were confirmed by membrane staining with Ponceau S (Sigma-Aldrich; St Louis, MO) in the case of secretome protein extracts and plasma samples or by measuring the amount of GAPDH or β-actin in whole tissue protein extracts. The membranes were blocked for 1 h in 5% Bovine Serum Albumin (BSA, Sigma-Aldrich, USA) and successively probed with primary antibodies over night at 4 °C and peroxide-conjugated secondary antibodies for 1 h at room temperature. Specific antigen-antibody binding was visualized using a chemiluminescence method according to the manufacturer’s instructions (Pierce ECL western blotting Sustrate, Thermo Scientific). Image J software (NIH, Bethesda, MD, USA) was used to quantify the volumes of specific bands.

Primary anti-FNDC5 and anti-PGC1α were purchased from Abcam (dilute 1:1000) (catalog ab93373 and ab191838 respectively, Cambridge, UK). Anti-Irisin was purchased from Phoenix Pharmaceuticals Inc. (1:1000) (catalog G-067–17; CA, USA). Primary anti-GAPDH was purchased from Life Technologies Ltd (1:5000) (catalog AM4300, Paisley, UK), and anti-β-actin from Sigma-Aldrich (1:5000) (catalog A-5316; St Louis, MO, USA). In addition, gastric expression and plasma levels of FNDC5 were verified by using a monoclonal FNDC5 antibody purchased from Abcam (1:1000) (catalog ab174833, Abcam, Cambridge, UK). We used goat anti-rabbit secondary antibody (1:5000) (catalog 111–035–003) in the case of FNDC5, irisin and PGC1α, and goat anti-mouse secondary antibody (1:10000) (catalog 115–035–003) in the case of GAPDH and β-actin. Both secondary antibodies were purchased by Jackson ImmunoResearch (Baltimore, USA) (Table 1).

Western blots were performed using independent samples from different rats belonging to each group. GAPDH detection was performed for all western blots as a loading control, except in brown adipose tissue (BAT) where β-actin was used. The western blot data are expressed as the mean ± SEM of percentages normalized to GAPDH or β-actin levels (arbitrary units).

In order to demonstrate that the anti-FNDC5 polyclonal antibody used is binding specifically to the antigen of interest an immunizing peptide blocking experiment was also performed. To do this, the primary antibody was preincubated with an excess of the immunogen (recombinant FNDC5 100 ng/ml, catalog AG-40B-0153 Adipogen AG, Switzerland) overnight at 4 °C. Then, the gastric tissue membrane was incubated with the neutralized antibody instead of the primary antibody alone. In addition, negative control without primary antibody was included to reconfirm the antibody specificity.

#### RNA isolation and real-time quantitative RT-PCR analysis

Total RNA was isolated from the stomach mucosa of animals using TRIzol (Invitrogen, CA, USA), according to the manufacturer’s recommendations. The extracted total RNA was purified with DNase treatment using a DNA-free kit as a template (Ambion, USA) to generate first-strand cDNAs using a high-capacity cDNA Reverse Transcription kit (Applied Biosystems, USA). Quantitative real-time PCR was performed using a StepOne Plus Instrument (Applied Biosystems) with specific Taqman qRT-PCR primers and probes (Table 2). For analysis, the FNDC5 gene expression levels were normalized using the mRNA expression of two known housekeeping genes as recommended by the guidelines of MIQUE (The Minimum Information for Publication of Quantitative Real-Time PCR Experiments)^22^, hypoxanthine phosphoribosyltransferase 1 (HPRT1) and eukaryotic 18S rRNA (18S) (TaqMan: Applied Biosystems). The delta-delta-Cp-method with the geometric mean of the two reference genes for normalization was performed^23^. Results are expressed as fold-changes in experimental group compared to a control group.

#### Immunohistochemical studies

The gastric wall samples were immersion fixed in 10% neutral buffered formalin for 24 h and embedded in paraffin. Four μm thick sections were mounted on FLEX IHC microscope slides (Dako-Agilent, Glostrup, Denmark) and heated in an oven at 60 °C for 1 h. Immunohistochemical analyses was automatically performed using an AutostainerLink 48 (Dako-Agilent).

After deparaffinization and epitope retrieval in EnVision FLEX target retrieval solution (high pH) for 20 min at 97 °C, the slides were allowed to cool in PT Link to 65 °C and then in Dako-Agilent Wash Buffer for 5 min at room temperature. The immunostaining protocol included the following steps: (1) EnVision FLEX peroxidase-blocking reagent (Dako) for 5 min; (2) rabbit monoclonal FNDC5 antibody (Abcam, catalog ab174833, Cambridge, UK) at a dilution of 1/100 for 30 min); (3) Envision FLEX Mouse Linker (Dako-Agilent) for 15 min; (4) EnVision FLEX/HRP (dextran polymer conjugated with horseradish peroxidase and affinity-isolated goat anti-mouse and anti-rabbit immunoglobulins) (Dako-Agilent) for 20 min; (5) substrate working solution (mix) (3,3′-diaminobenzidine tetrahydrochloride chromogen solution) (Dako-Agilent) for 10 min and (6) EnVision FLEX hematoxylin (Dako-Agilent) for 9 min.

#### Biochemical analysis

Leptin levels in plasma were determined with a commercial enzyme-linked immunosorbent assay (ELISA) kit purchased from Millipore/DRG (catalog #EZRL-83K, Billerica, MA, USA). The assay sensitive limit was 0.08 ng/ml. The results are presented as ng/ml.

### Statistical analyses

Data are presented as the mean ± SEM. Statistical analyses were performed using the SPSS version 20.0 software statistical package (SPSS, Chicago, IL). Data were analyzed using one-way ANOVA (normally distributed data) or Kruskal-Wallis test (non-normally distributed data). A p value less than 0.05 was considered to be statistically significant. The statistical significance is indicated as follows: *p < 0.05, **p < 0.01 and ***p < 0.001. Given the small data set of this work a power study was carried out to determine the convenience of the correlation analysis. The results showed a β error higher than 0.20 therefore the correlation analysis was rejected. In order to show a dependency among data, plots of individual data points with marked groups was performed.

## Results

### Full length FNDC5 protein is expressed in the stomach

**Figure 1:**
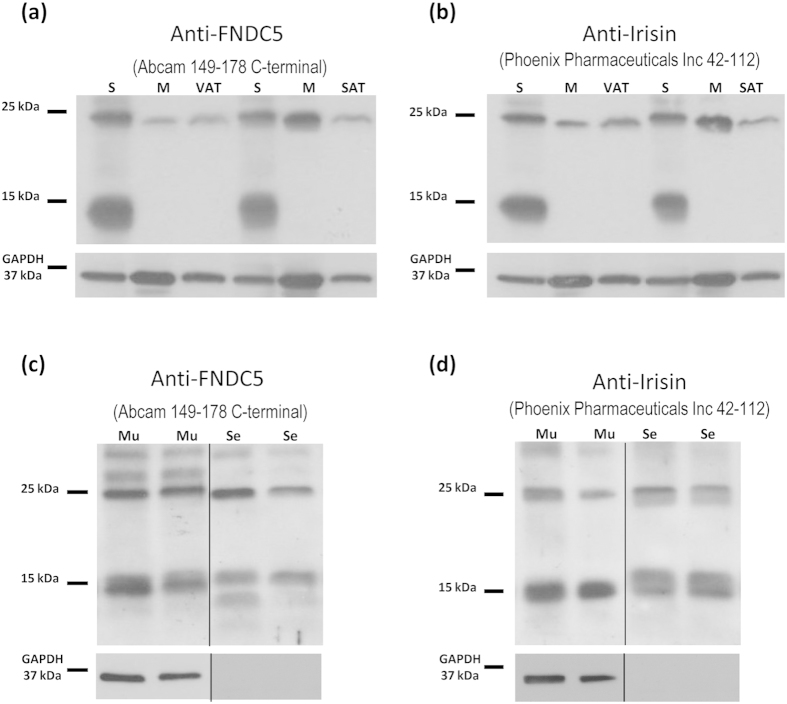
Representative immunoblot images showing the full length FNDC5 protein. The presence of a 25 kDa band was detected in the stomach with anti-FNDC5 (**a**) and anti-Irisin (**b**) antibodies as well as in subcutaneous and visceral adipose tissue and muscle. In the stomach, the FNDC5 band of 25 kDa was detected both in mucosa and secretomes with anti-FNDC5 (**c**) and anti-Irisin (**d**) antibodies. GAPDH was used as a loading control. Dividing lines indicate splicing of the same gel. Full-length blots are presented in Supplementary Figure 1. M: muscle; Mu: gastric mucosa; S: stomach; Se, gastric secretoma; SAT: subcutaneous adipose tissue; VAT: visceral adipose tissue.

Protein levels of FNDC5/Irisin were assessed by western blot with two different antibodies: 1) specific polyclonal antibody directed against FNDC5 (amino acids 149–178 from Abcam) and 2) an antibody directed against Irisin (amino acids 42–112 from Phoenix Pharmaceuticals). By using these anti-FNDC5/irisin antibodies, high level of expression was found in gastric mucosa and, as previously published^12^, in muscle, visceral and subcutaneous adipose tissues of ad libitum standard diet-fed animals. The band corresponding to the full length FNDC5 protein had the predicted molecular size of 25 kDa (Fig. 1a) previously showed in the bibliography for other tissues^11^,^12^. As formerly described^12^, with the anti-Irisin antibody it was not possible to detect the irisin peptide at the predicted size (12 kDa) in any of the studied samples (Fig. 1). However, the 25 kDa band was also found with the anti-Irisin antibody (Fig. 1b), which after densitometry analysis was shown to be regulated identically to the 25 kDa band found with the anti-FNDC5 antibody. This fact highlights the possibility that both antibodies recognize the FNDC5 protein.

The western blot assay of the secretomes obtained from the gastric mucosa explants model showed that the 25 kDa FNDC5 was secreted, and the protein was identified in gastric secretomes independently of the antibody used (Fig. 1c,d). These findings prove that the stomach secretes FNDC5. Accordingly, the results obtained from secretion and location prediction software shows that FNDC5 is in the secretory pathway containing signal peptide (supplementary information).

When we focused on the full immunoblot images of Fig. 1a,b, a very strong 15 kDa band was detected in the gastric mucosa that was absent in muscle or adipose tissue. This band was detected with both antibodies, anti-FNDC5 (Fig. 1a) and anti-Irisin (Fig. 1b), which clearly discards the possibility that this band corresponds to irisin. The results obtained from quantification of the 15 kDa band (Supplementary Figure 2) showed an identical regulation to the 25 kDa band. In addition, this band was detected in the secretomes from gastric mucosa (Fig. 1c,d). Because it was not previously described any 15 kDa FNDC5 isoform in rat, and considering that we were not able to prove by mass spectrometry if this band contained any form related to FNDC5 (Supplementary information) we focused the succeeding study on the 25 kDa band corresponding to FNDC5.

**Figure 2:**
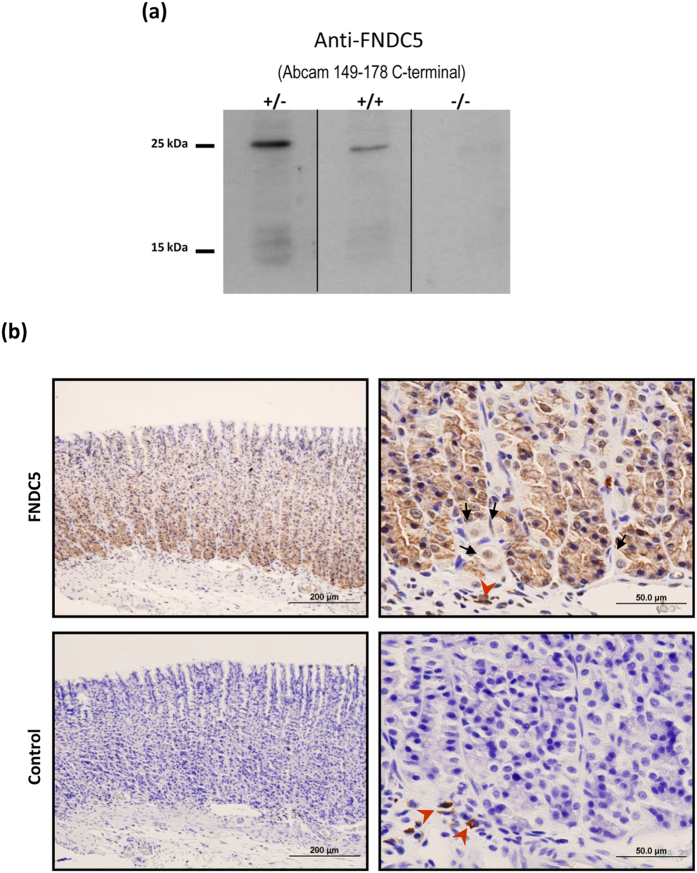
FNDC5 antibody specificity assay and immunohistochemical study. (**a**) Blockade of the FNDC5 antibody and negative control. (+/−) FNDC5 antibody not preincubated with the immunogen, (+/+) FNDC5 antibody preincubated with FNDC5 recombinant protein, (−/−) negative control without primary antibody. Dividing lines indicate splicing of the same gel. Full-length blot are presented in Supplementary Figure 1. (**b**) Photomicrographs of the FNDC5 immunohistochemical studies in gastric tissue from ad libitum fed rats. FNDC5 immunoreactivity was mainly located at the bottom of the gastric glands (upper left panel). Detail of the bottom of gastric glands that show the FNDC5 positive cells (upper right panel). These cells might be identified as chief cells by their morphology and localization. The arrows indicate FNDC5 no immunoreactive cells, which were identified as parietal cells (occasional round cells with large, centrally located nuclei) (upper right panel). The corresponding negative controls are showed in the lower panels. The red arrowheads show non-specific immunoreactive cells situated at the lamina propia (probably macrophages).

In order to test the specificity of the FNDC5 antibody, a western blot assay was performed by previously blocking the antibody with a FNDC5 recombinant protein. This assay showed that the band corresponding to 25 kDa FNDC5 was significantly reduced in gastric mucosa (Fig. 2a). Additionally, the negative control without primary antibody allowed reconfirming the antibody specificity (Fig. 2a).

With the aim of investigating which type of gastric cells are the main source of FNDC5 production, immunohistochemical studies using a specific monoclonal antibody for FNDC5 were performed on gastric fundus sections obtained from ad libitum fed rats. The FNDC5 immunoreactivity was found in cells situated primarily at the bottom of the gastric glands (Fig. 2b upper left panel). The morphology (columnar cells) and localization (basal third of the gastric glands) of these cells might let to identify them as chief cells. Parietal cells were no immunoreactive for FNDC5 (Fig. 2b upper right panel). The negative controls by omitting FNDC5 antibody are shown in Fig. 2b down panels.

### Influence of a high fat diet on FNDC5 expression in the gastric mucosa of rats

To study the effect of the body composition on the FNDC5 levels, two groups of male rats were used: one group was fed a standard chow diet (C) and another group was fed a high fat diet (HF) for 13 weeks after weaning.

**Figure 3:**
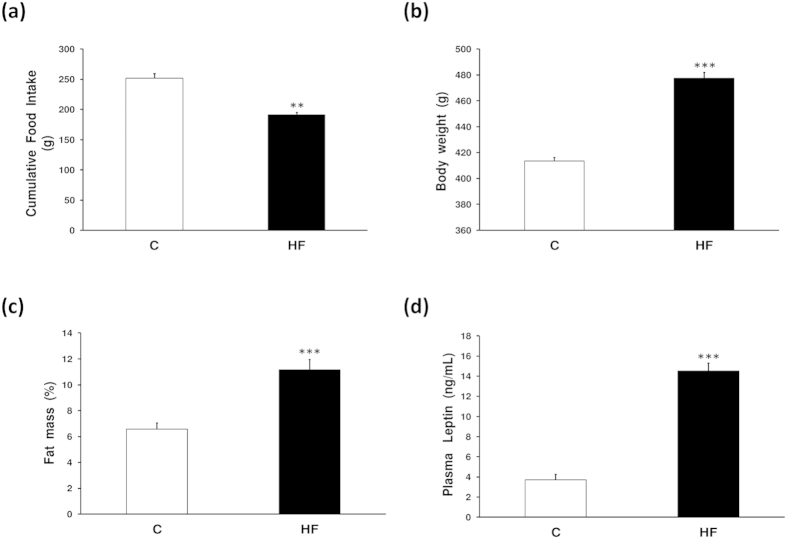
Effects of diet on energy homeostasis. (**a**) Cumulative food intake, (**b**) body weight, (**c**) fat mass and (**d**) leptin circulating levels measured by ELISA. Data are expressed as the mean ± SEM. N = 10. Error bars indicate the SEM. **P < 0.01, ***P < 0.001 vs. Control. C, control; HF, high fat diet.

As expected in high fat diet fed animals, the amount of food intake in grams was significantly decreased; probably due to the more satiating effect of this food (Fig. 3a). The manipulation of the diet composition caused the expected changes in body weight, with increased body weight in the group fed with a high fat diet (Fig. 3b). The amount of fat mass was also increased in animals with a high fat diet with respect to the chow-fed animals (Fig. 3c). Accordingly to the fat mass increase in high fat diet fed animals, an increase in leptin circulating levels were found in obese animals with respect to controls (Fig. 3d).

**Figure 4:**
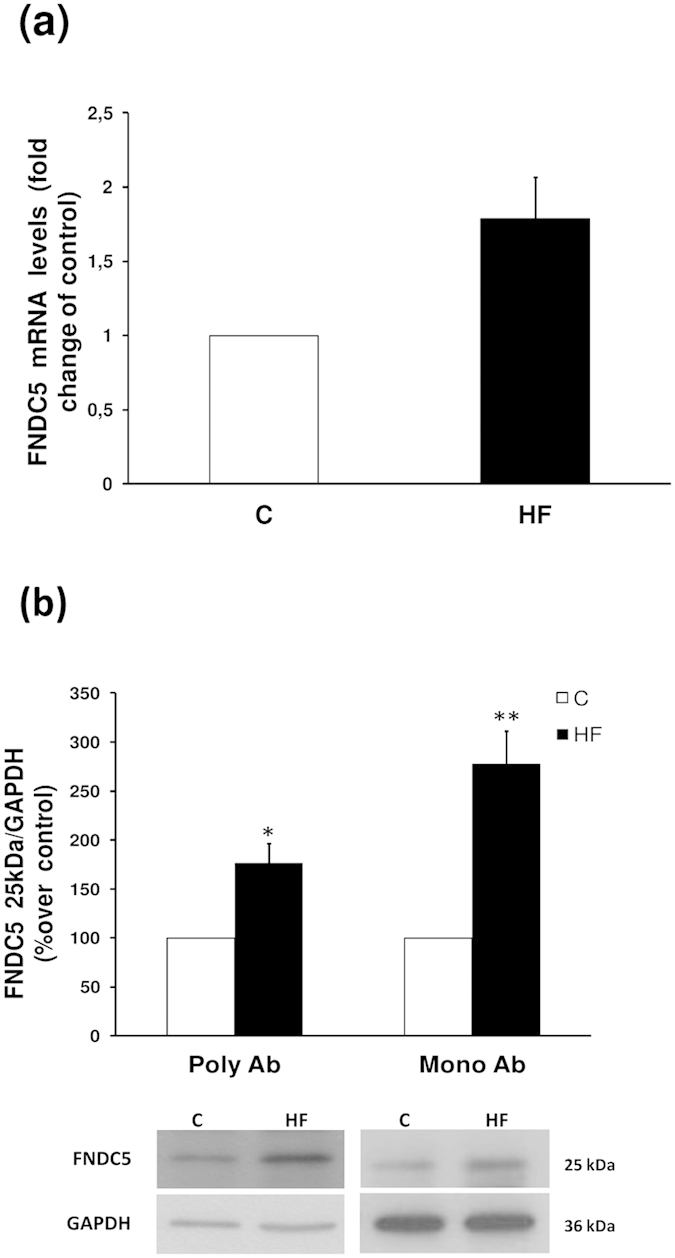
Expression of the full lenght FNDC5 protein in gastric mucosa. (**a**) FNDC5 mRNA levels measured by real-time PCR. (**b**) FNDC5 protein levels and representative western blot images from 25 kDa FNDC5 protein detected with two FNDC5 antibodies (Poly Ab, policlonal antibody; Mono Ab, monoclonal antibody). GAPDH was used to normalize the protein levels. Values are presented as the mean ± SEM. N = 6–8. Error bars indicate the SEM. *P < 0.05, **P < 0.01 vs. Control. C, control; HF, high fat diet.

Expression studies by the real time-PCR performed in the stomachs from the above animals showed a tendency towards an increase in the mRNA levels of FNDC5 in the gastric mucosa of obese animals (Fig. 4a).

At protein level FNDC5 immunoblots showed that the diet with a high fat content caused an increase of FNDC5 expression in the gastric lysate (Fig. 4b). In order to reconfirm the obtained data, the immunodetection studies were also performed with a new released monoclonal antibody purchased from Abcam. The results obtained with both FNDC5 antibodies were identical (Fig. 4b).

### Influence of the body composition on FNDC5 secretion by the stomach and circulating levels

**Figure 5:**
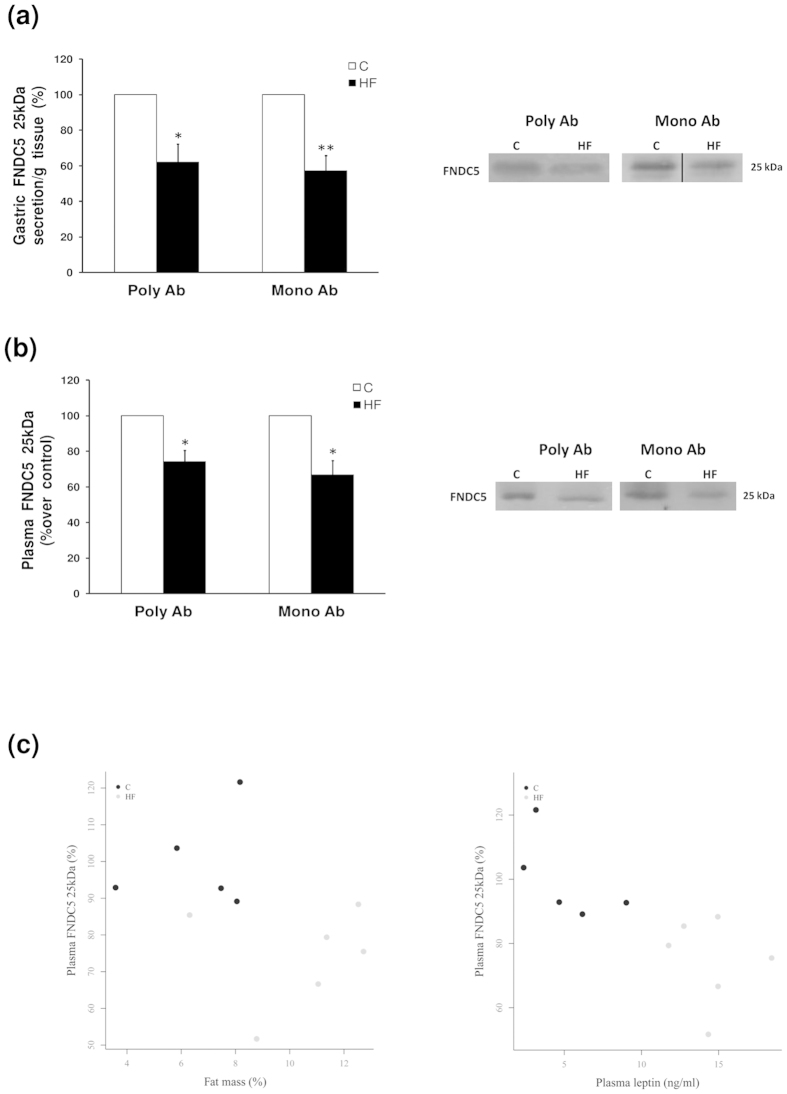
FNDC5 secretion from gastric explants and circulating levels. (**a**) FNDC5 protein levels and representative western blot images detected with two FNDC5 antibodies (Poly Ab, policlonal antibody; Mono Ab, monoclonal antibody). Dividing lines indicate splicing of the same gel. Full-length blot are presented in Supplementary Figure 1. (**b**) Plasma FNDC5 protein levels and representative western blot images from FNDC5 peptide detected with two FNDC5 antibodies (Poly Ab, policlonal antibody; Mono Ab, monoclonal antibody). (**c**) Plots of individual data points represent dependency among plasma FNDC5 levels with fat mass and plasma leptin, showing clearly separated clouds of data points. Values are expressed as the mean ± SEM. N = 6–8. Error bars indicate the SEM. *P < 0.05, **P < 0.01 vs. Control. C, control; HF, high fat diet.

Western blot analysis of the secretome, obtained from the gastric explants system developed in the experimental models, showed that FNDC5 is secreted from the stomach to the extracellular medium. Contrary to the data on the gastric mucosa content, obese rats presented a decreased secretion of FNDC5 (Fig. 5a).

The data in this study unequivocally showed that the stomach secreted FNDC5 and that this secretion is modulated by body composition; therefore, the next step was to prove if this secretion was reflected in the circulating levels of FNDC5. To do this, given the controversy over the FNDC5 commercial kits, we decided to perform the western blot study of the plasma with two antibodies (poyclonal and monoclonal) against FNDC5. Immunoblots showed that the diet with a high fat content produced a decrease in FNDC5 circulating levels (Fig. 5b). In addition, plasmatic levels of FNDC5 were negatively associated with fat mass and circulating leptin as showed by the individual data plot (Fig. 5c).

### PGC1α expression in stomach and BAT

**Figure 6:**
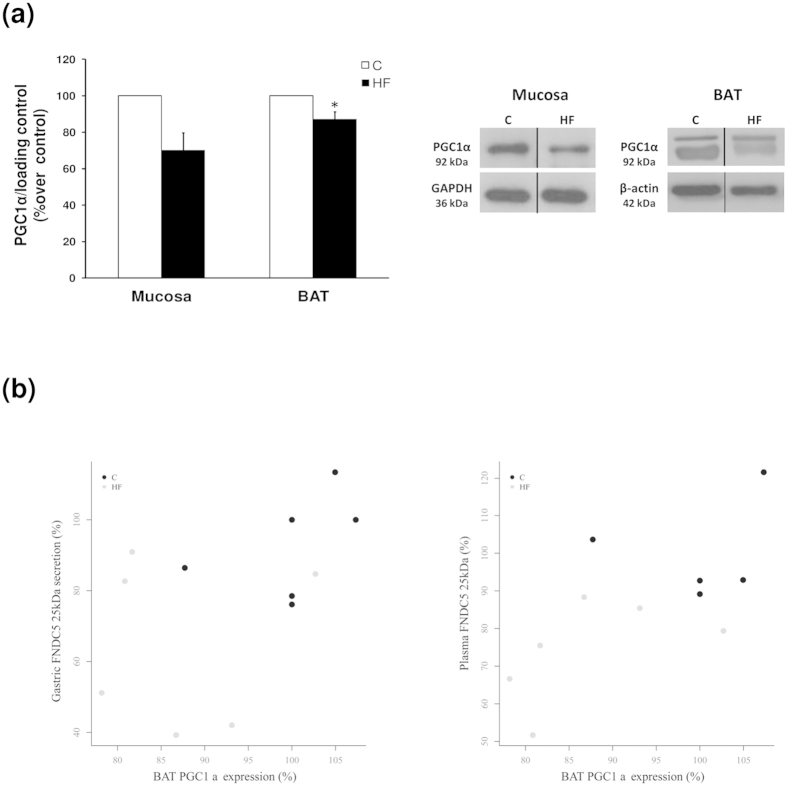
PGC1α expression in stomach and BAT. (**a**) PGC1α protein levels and representative western blot images in gastric mucosa and BAT. GAPDH and β-actin was used to normalize the protein levels. Dividing lines indicate splicing of the same gel. Full-length blots are presented in Supplementary Figure 1. (**b**) Plots of individual data points represent dependency of PGC1α expression in BAT with FNDC5 gastric secretion and circulating levels, showing clearly separated clouds of data points. Values are expressed as the mean ± SEM. N = 6–8. Error bars indicate the SEM. *P < 0.05 vs. Control. C, control; HF, high fat diet. BAT, brown adipose tissue.

PGC1α protein expression was studied in gastric mucosa and BAT by western blot analysis. Rats with obesity induced by diet showed a tendency towards a reduction in gastric PGC1α expression compared with the lean animals (Fig. 6a). In the case of BAT, western blot analysis clearly showed that obese animals under a high fat diet presented a decrease in PGC1α expression compared with the lean controls (Fig. 6a). Additionally, a positive association was observed between PGC1α expression in BAT and gastric FNDC5 secretion (Fig. 6b). Moreover, plasmatic levels of FNDC5 were positively associated with BAT PGC1α expression (Fig. 6b).

## Discussion

The main findings of the present paper are: first, the FNDC5 gene is expressed in gastric mucosa, being the chief cells the main source of FNDC5 in the stomach. Second, FNDC5 is secreted directly from the stomach, and it is regulated by body composition. Third, the rate of FNDC5 gastric secretion was related with its circulating levels and with PGC1 expression in BAT.

As previously reported^1^,^3^,^24^,^25^,^26^, the gastrointestinal tract is crucial for energy homeostasis regulation. Currently, an increasing number of signals secreted from the gastrointestinal tract are proposed as components to the gastrointestinal-brain-adipose tissue axe in charge of body weight regulation^24^,^25^,^26^. FNDC5/irisin was initially described in muscle^10^ and later in adipose tissue^12^. In addition, the immunopositivity of FNDC5 was very recently found in diverse peripheral tissues, such as the testis, pancreas, liver, spleen and human stomach^27^. A potential role for irisin in protecting against obesity-associated disorders has been proposed based on the initial article published by Bostrom *et al.* 2012 in Nature^10^, which reported that FNDC5 overexpression in mice provokes browning in WAT. Moreover, it was shown that after a moderate increase in the circulating irisin levels, an increase in energy expenditure and a reduced body weight gain as well as an improved insulin resistance was induced under high fat diet. Under this context the hypothesis of the present work proposes that FNDC5 might be also produced by the stomach as a major organ involved in energy homeostasis; this secretion at gastric level could be deregulated in obesity.

The present results reveal for the first time to the best of our knowledge, FNDC5 mRNA expression in gastric mucosa by real time PCR. Moreover, the present data showed FNDC5 protein content in gastric mucosa by western blot assay. Full length FNDC5 protein of 25 kDa was detected in the rat stomach accordingly to previous work by other groups in muscle^11^ and adipose tissue^12^ that used antibodies targeting the same FNDC5 carboxiterminal region (aa 149–178) that we have used in the present work. The specificity of the FNDC5 band was showed by including the negative controls without primary antibody and with the blocked antibody after incubation with recombinant FNDC5.

It should be noted that, in the present paper, western blots assays were performed by triplicate with three different antibodies, two of them against FNDC5 (monoclonal and polyclonal antibodies) and with the anti-Irisin antibody. The present data showed that FNDC5 vary with body composition in an identical fashion independently of the used antibody. This finding suggests that antibodies recognize FNDC5, showing identical bands at the same molecular weight (Fig. 1a,b), as previously suggested by several authors^28^.

Lee *et al.*, recently detected an irisin peptide by mass spectrometry study and determined that the 25 kDa band found in human serum samples represents FNDC5 or FNDC5 fragments^28^. The most recent demonstration of circulating irisin detection in plasma was relied in a work of Jedrychowski *et al.*, where they detect and quantify human irisin in plasma by quantitative mass spectrometry showing that irisin is translated from its non-canonical start codon^29^. However, the ~12 kDa irisin band was only detected by western blot in muscle and serum of mice by Brenmoehl *et al.* in 2014 with an anti-irisin antibody developed using full length recombinant irisin^11^. With the antibodies used in the present work we were not able to detect the 12 kDa irisin. Consequently in the present work we exclusively focus the study in FNDC5 expression and production from the stomach, and not in the soluble form irisin. Thus, all the shown changes correspond to the results obtained with the anti-FNDC5 antibodies.

In addition to the full length FNDC5 protein band at 25 kDa found in the immunoblots in all of the tested tissues (gastric mucosa, muscle and adipose tissue), another band of approximately 15 kDa was found expressed exclusively in the gastric mucosa (Fig. 1). The results obtained from the western blot quantification showed an identical regulation for the 15 and 25 kDa bands with body composition (Supplementary Figure 2). However, after mass spectrometry analysis through two different approaches we were not able of identify FNDC5 or FNDC5 derived peptides in this lower weight band (Supplementary Information). Accordingly, the findings in the present paper are focused in the data obtained for the full length FNDC5 protein band at 25 kDa, which was previously confirmed in other tissues different from the stomach^11^,^12^.

The present paper is the first to display immunohistochemically the location of FNDC5 protein in gastric chief cells of rat stomach. It should be taken into account that gastric chief cells are the type of cells that release pepsinogen^30^. In addition, this type of cells also secretes gastric lipase enzymes, involved in lipids digestion, and leptin in response to food ingestion. The presence of FNDC5 in this sort of cells strongly reinforces the idea of a direct role of gastric FNDC5 in energy homeostasis regulation by the gastrointestinal tract. Moreover, the observed variations on FNDC5 production by the stomach shown for the first time,in an animal model of obesity supports the potential role of gastric FNDC5 on the gastric chief cells function on energy metabolism.

In addition to the expression of FNDC5 in gastric tissue and concretely in the gastric chief cells, the present work shows for the first time that FNDC5 is secreted from the stomach. Thus, the full length of FNDC5 protein was detected by western blot,in the gastric secretomes obtained from an *ex vivo* gastric tissue explants system developed by our group^3^. Because this finding might seem contradictory considering that FNDC5 is a membrane protein, an exhaustive study was performed by using a secretion and location prediction software to fully clarify this point (supplementary information). By the use of this software it was found that FNDC5 is in the secretory pathway containing signal peptide, which explains the FNDC5 secretion from gastric tissue described in the present work.

The most interesting finding regarding gastric FNDC5 secretion is that its secretion decreased with fat mass increase. Moreover, in the studied models it was found that full length FNDC5 circulating levels detected by western blot in rat plasma with both, polyclonal and monoclonal FNDC5 antibodies, were also decreased in diet-induced obesity. Therefore, in obesity the levels of circulating FNDC5 were decreased, together with a decrease in FNDC5 secretion from the stomach. Differently to the present data, a previous report shows a significant increase of circulating FNDC5/irisin in DIO rats^12^. This discrepancy might be due to the obesity model which differs in diet composition and time exposition to the diet, and certainly due the different animal age. Thus, previous work showing a positive correlation of circulating FNDC5/irisin levels with increased BMI and fat mass were performed in adult individuals^15^,^16^,^17^. Moreover, it should be taken into account that the cited work refers to a 25 kDa band identified with the antibody against the soluble form irisin while the present data was recorded with two different antibodies against full FNDC5. Indeed this fact highlights once again the necessity of accurate FNDC5/irisin commercial antibodies due to the great uncertainty in detecting the real irisin^15^.

The data in the present paper showing a reduced gastric production of FNDC5 in obese animals reinforce the initial idea of a protective effect of FNDC5/Irisin against obesity, which fit well with a decrease in energy expenditure reported in diet induced obese animals^31^. Bostrom study^10^ shows that overexpression of the complete FNDC5 induces browning and thermogenic program. BAT is specialized in energy expenditure through adaptive thermogenesis, which is a physiological mechanism by which energy is dissipated to generate heat in response to cold temperatures and diet^32^. PGC1α has been suggested to be a central and indispensable mediator in BAT mitochondrial thermogenesis^33^. The present paper showed that the gastric FNDC5 secretion pattern is positively related with PGC1α expression in BAT, suggesting a potential role of gastric FNDC5 in the PGC1α–mediated thermogenesis of BAT and contributing to the proposed effect of FNDC5 in increasing energy expenditure.

The present work shows in rats, for the first time, that the gastric production of FNDC5 follows changes in the body composition. It should be defined whether changes in the fat mass are responsible for the variations in the secretion from the stomach and the circulating levels of FNDC5 or if, on the contrary, the modifications at this level act directly on adipose tissue. Either way, all together the data in the present paper indicate that there may be an unexplored communication among the stomach and adipose tissue mediated by FNDC5. Reinforcing this theory, in 2012 Huh *et al.*^9^ reported that bariatric surgery led to significant weight loss after 6 months, which was accompanied by a decrease in the circulating irisin levels attributed to a diminution in fat mass. However, it should be also considered that the restriction of the gastric size under bariatric surgery may also decrease gastric contribution to circulating FNDC5 levels, explaining the reduced irisin circulating levels found 6 months after the intervention. Further work should asses this possibility. It should also be taken into account that, contrary to the reduction in irisin circulating levels after bariatric surgery, another recently reported article has found no variations in FNDC5/Irisin circulating levels after this intervention^21^. In humans a controversy exists regarding the relation among the circulating irisin levels and BMI^9^,^13^,^18^,^34^. The most recent studies have found a positive correlation between BMI and circulating irisin^15^ which conflicts with the beneficial and antiobesity effect of irisin^10^. The discrepancies in circulating irisin measurements published in the last years has led some authors to propose that some of the available ELISA kits are unspecific and might present cross reactivity to other proteins^35^,^36^. Under this context, it should be taken into account that the bariatric surgery data were recorded in humans while the data in the present paper are extracted from animal and so the contribution of the gastric FNDC5 cannot be extrapoled to the beneficial effects of gastric surgery in humans.

In sum, for the first time, the data in the present paper in rat model provide a new source of FNDC5 in the organism, the stomach. The present work prove that FNDC5 is secreted from the stomach and this secretion is reflected in the circulating levels and regulated by the body composition, suggesting a potential physiological role of gastric FNDC5 in regulating energy expenditure and body weight homeostasis.

In conclusion, the present work reveals FNDC5 as one of the gastrointestinal derived signals that form part of the gastrointestinal-adipose tissue axe involved in energy homeostasis regulation.

## Additional Information

**How to cite this article**: Barja-Fernández, S. *et al.* FNDC5 is produced in the stomach and associated to body composition. *Sci. Rep.* **6**, 23067; doi: 10.1038/srep23067 (2016).

## Supplementary Material

Supplementary Information

## Figures and Tables

**Table 1:** Antibodies used in western blot analysis.

Antibody	Manufacturer	Catalog No	Clonality	Source/Host	Dilution
*Primary antibodies*					
Anti-FNDC5	Abcam	ab93373	Polyclonal	Rabbit	1:1000
Anti-Irisin	Phoenix Pharmaceuticals	G-067-17	Polyclonal	Rabbit	1:1000
Anti-FNDC5	Abcam	ab-174833	Monoclonal	Rabbit	1:1000
Anti-PGC1α	Abcam	ab191838	Polyclonal	Rabbit	1:1000
Anti-GAPDH	Life Technologies	AM4300	Monoclonal	Mouse	1:5000
Anti-β-actin	Sigma-Aldrich	A5316	Monoclonal	Mouse	1:5000
*Secondary antibodies*					
Anti-Rabbit	Jackson ImmunoResearch	111-035-003	Polyclonal	Goat	1:5000
Anti-Mouse	Jackson ImmunoResearch	115-035-003	Polyclonal	Goat	1:10000

**Table 2:** Primers for real time qPCR analysis.

Gene symbol	Gene description	TaqMan gene expression assay number	GenBank accession number
HPRT	Hypoxanthinephosphoribosyltransferase 1 (REFERENCE GENE)	Rn01527840_m1	NM_012583.2
18 S	Eukaryotic 18 S rRNA (REFERENCE GENE)	Hs99999901_s1	X03205
FNDC5	Fibronectin type III domain containing 5	Rn01519161_m1	NM_001270981.1

## References

[b1] Folgueira C., Seoane L. M. & Casanueva F. F. The Brain-Stomach Connection. in How Gut and Brain Control Metabolism, Vol. 42 (eds Delhanty P. J. D. & VanDerLely A. J. ) 83–92 (2014). 10.1159/000358316 24732927

[b2] Scholtz S. *et al.* Obese patients after gastric bypass surgery have lower brain-hedonic responses to food than after gastric banding. Gut 63, 891–902 (2014). 23964100 10.1136/gutjnl-2013-305008 PMC4033279

[b3] Seoane L. M. *et al.* Sensory stimuli directly acting at the central nervous system regulate gastric ghrelin secretion. An *ex Vivo* organ culture study. Endocrinology 148, 3998–4006 (2007). 17495002 10.1210/en.2007-0226

[b4] Seoane L. M., Al-Massadi O., Barreiro F., Dieguez C. & Casanueva F. F. Growth hormone and somatostatin directly inhibit gastric ghrelin secretion. An *in vitro* organ culture system. Journal of Endocrinological Investigation 30, RC22–RC25 (2007). 17993760 10.1007/BF03350806

[b5] Al-Massadi O. *et al.* Age, sex, and lactating status regulate ghrelin secretion and GOAT mRNA levels from isolated rat stomach. American Journal of Physiology-Endocrinology and Metabolism 299, E341–E350 (2010). 20501877 10.1152/ajpendo.00057.2010

[b6] Barja-Fernandez S., Leis R., Casanueva F. F. & Seoane L. M. Drug development strategies for the treatment of obesity: how to ensure efficacy, safety, and sustainable weight loss. Drug Design Development and Therapy 8, 2391–2400 (2014). 10.2147/DDDT.S53129 PMC4257050 25489237

[b7] Ferrer-Martinez A., Ruiz-Lozano P. & Chien K. R. Mouse PeP: a novel peroxisomal protein linked to myoblast differentiation and development. Dev Dyn 224, 154–67 (2002). 12112469 10.1002/dvdy.10099

[b8] Teufel A., Malik N., Mukhopadhyay M. & Westphal H. Frcp1 and Frcp2, two novel fibronectin type III repeat containing genes. Gene 297, 79–83 (2002). 12384288 10.1016/s0378-1119(02)00828-4

[b9] Huh J. Y. *et al.* FNDC5 and irisin in humans: I. Predictors of circulating concentrations in serum and plasma and II. mRNA expression and circulating concentrations in response to weight loss and exercise. Metabolism 61, 1725–38 (2012). 23018146 10.1016/j.metabol.2012.09.002 PMC3614417

[b10] Bostrom P. *et al.* A PGC1-alpha-dependent myokine that drives brown-fat-like development of white fat and thermogenesis. Nature 481, 463–8 (2012). 22237023 10.1038/nature10777 PMC3522098

[b11] Brenmoehl J. *et al.* Irisin is elevated in skeletal muscle and serum of mice immediately after acute exercise. Int J Biol Sci 10, 338–49 (2014). 24644429 10.7150/ijbs.7972 PMC3957089

[b12] Roca-Rivada A. *et al.* FNDC5/Irisin Is Not Only a Myokine but Also an Adipokine. PLoS One 8, 10.1371/journal.pone.0060563. (2013). PMC3623960 23593248

[b13] Stengel A. *et al.* Circulating levels of irisin in patients with anorexia nervosa and different stages of obesity–correlation with body mass index. Peptides 39, 125–30 (2013). 23219488 10.1016/j.peptides.2012.11.014

[b14] Park K. H. *et al.* Diet quality is associated with circulating C-reactive protein but not irisin levels in humans. Metabolism 63, 233–41 (2014). 24315778 10.1016/j.metabol.2013.10.011 PMC4373656

[b15] Pardo M. *et al.* Association of irisin with fat mass, resting energy expenditure, and daily activity in conditions of extreme body mass index. Int J Endocrinol 2014, 857270 (2014), 10.1155/2014/857270. 24864142 PMC4016898

[b16] Crujeiras A. B. *et al.* Longitudinal variation of circulating irisin after an energy restriction-induced weight loss and following weight regain in obese men and women. Am J Hum Biol 26, 198–207 (2014). 24375850 10.1002/ajhb.22493

[b17] Crujeiras A. B., Pardo M. & Casanueva F. F. Irisin: ‘fat’ or artefact. Clin Endocrinol (Oxf) 82, 467–74 (2015). 25287317 10.1111/cen.12627

[b18] Moreno-Navarrete J. M. *et al.* Irisin is expressed and produced by human muscle and adipose tissue in association with obesity and insulin resistance. J Clin Endocrinol Metab 98, E769–78 (2013). 23436919 10.1210/jc.2012-2749

[b19] Sanchis-Gomar F. *et al.* Circulating irisin levels are not correlated with BMI, age, and other biological parameters in obese and diabetic patients. Endocrine 46, 674–7 (2014). 24510629 10.1007/s12020-014-0170-9

[b20] Sanchis-Gomar F. & Perez-Quilis C. Irisinemia: a novel concept to coin in clinical medicine? Ann Nutr Metab 63, 60–1 (2013). 23941905 10.1159/000354090

[b21] Gutierrez-Repiso C. *et al.* FNDC5 could be regulated by leptin in adipose tissue. Eur J Clin Invest 44, 918–25 (2014). 25112714 10.1111/eci.12324

[b22] Bustin S. A. *et al.* The MIQE guidelines: minimum information for publication of quantitative real-time PCR experiments. Clin Chem 55, 611–22 (2009). 19246619 10.1373/clinchem.2008.112797

[b23] Hellemans J., Mortier G., De Paepe A., Speleman F. & Vandesompele J. qBase relative quantification framework and software for management and automated analysis of real-time quantitative PCR data. Genome Biol 8, R19, 10.1186/gb-2007-8-2-r19 (2007). 17291332 PMC1852402

[b24] Barja-Fernandez S. *et al.* Peripheral signals mediate the beneficial effects of gastric surgery in obesity. Gastroenterol Res Pract 2015, 560938, 10.1155/2015/560938 (2015). 25960740 PMC4413036

[b25] Folgueira C. *et al.* Uroguanylin action in the brain reduces weight gain in obese mice via different efferent autonomic pathways. Diabetes, 10.2337/db15-0889 (2015). 26566631

[b26] Senin L. L. *et al.* Regulation of NUCB2/nesfatin-1 production in rat’s stomach and adipose tissue is dependent on age, testosterone levels and lactating status. Mol Cell Endocrinol 411, 105–12 (2015). 25916958 10.1016/j.mce.2015.04.016

[b27] Aydin S. *et al.* A comprehensive immunohistochemical examination of the distribution of the fat-burning protein irisin in biological tissues. Peptides 61, 130–6 (2014). 25261800 10.1016/j.peptides.2014.09.014

[b28] Lee P. *et al.* Irisin and FGF21 are cold-induced endocrine activators of brown fat function in humans. Cell Metab 19, 302–9 (2014). 24506871 10.1016/j.cmet.2013.12.017 PMC7647184

[b29] Jedrychowski M. P. *et al.* Detection and Quantitation of Circulating Human Irisin by Tandem Mass Spectrometry. Cell Metab 22, 734–40 (2015). 26278051 10.1016/j.cmet.2015.08.001 PMC4802359

[b30] Ramsey V. G. *et al.* The maturation of mucus-secreting gastric epithelial progenitors into digestive-enzyme secreting zymogenic cells requires Mist1. Development 134, 211–22 (2007). 17164426 10.1242/dev.02700

[b31] Corbett S. W., Stern J. S. & Keesey R. E. Energy expenditure in rats with diet-induced obesity. Am J Clin Nutr 44, 173–80 (1986). 3728354 10.1093/ajcn/44.2.173

[b32] Cannon B. & Nedergaard J. Brown adipose tissue: function and physiological significance. Physiol Rev 84, 277–359 (2004). 14715917 10.1152/physrev.00015.2003

[b33] Lin J. *et al.* Defects in adaptive energy metabolism with CNS-linked hyperactivity in PGC-1alpha null mice. Cell 119, 121–35 (2004). 15454086 10.1016/j.cell.2004.09.013

[b34] Timmons J. A., Baar K., Davidsen P. K. & Atherton P. J. Is irisin a human exercise gene? Nature 488, E9–10; discussion E10-1 (2012). 22932392 10.1038/nature11364

[b35] Albrecht E. *et al.* Irisin - a myth rather than an exercise-inducible myokine. Sci Rep 5, 8889 (2015). 25749243 10.1038/srep08889 PMC4352853

[b36] Erickson H. P. Irisin and FNDC5 in retrospect: An exercise hormone or a transmembrane receptor? Adipocyte 2, 289–93 (2013). 24052909 10.4161/adip.26082 PMC3774709

