# Supplementary material for: FNDC5 is produced in the stomach and associated to body composition
Source: Sci Rep. 2016 Mar 10;6:23067. doi: 10.1038/srep23067 (PMC4785389; doi:10.1038/srep23067)
Supplement: Supplementary Information [file srep23067-s1.pdf]

Manuscript title: FNDC5 is produced in the stomach and regulated by body fat composition

Authors: Barja-Fernández S, Folgueira C, Castelao C, Al-Massadi O, Bravo SB, Garcia-Caballero T, Leis R, Pardo M, Casanueva FF and Seoane LM.

Results obtained from an analysis with location and secretion prediction software of the full length FNDC5.

Results of the secretion and location prediction software showing that FNDC5 is in the secretory pathway containing signal peptide:

SecretomeP: http://www.cbs.dtu.dk/services/SecretomeP/

# Name	NN-score	Odds	Weighted	Warning
Sequence	0.185	0.374	0.001	signal peptide predicted by **SignalP**

===

===

Non-classically secreted proteins should obtain an NN-score / SecP score exceeding the threshold, but not at the same time be predicted to contain a signal peptide.
The recommended thresholds are 0.5 for bacterial sequences and 0.6 for mammalian sequences.

TargetP: http://www.cbs.dtu.dk/services/TargetP/

### targetp v1.1 prediction results ##################################
Number of query sequences: 1
Cleavage site predictions included.
Using NON-PLANT networks.

Name	Len	mTP	SP	other	Loc	RC	TPlen
Sequence	212	0.172	0.735	0.039	S	3	31

--

cutoff **0.000 0.000 0.000**

Legend:

**Name**	Sequence name truncated to 20 characters
**Len**	Sequence length
**cTP, mTP, SP, other**	Final NN scores on which the final prediction is based (Loc, see below). Note that the scores are not really probabilities, and they do not necessarily add to one. However, the location with the highest score is the most likely according to TargetP, and the relationship between the scores (the reliability class, see below) may be an indication of how certain the prediction is.
**Loc**	Prediction of localization, based on the scores above; the possible values are:<br>**C** Chloroplast, i.e. the sequence contains **cTP**, a chloroplast transit peptide;<br>**M** Mitochondrion, i.e. the sequence contains **mTP**, a mitochondrial targeting peptide;<br>**S** Secretory pathway, i.e. the sequence contains **SP**, a signal peptide;<br>**_** Any other location;<br>***** "don't know"; indicates that cutoff restrictions were set (see instructions) and the winning network output score was below the requested cutoff for that category.
**RC**	Reliability class, from 1 to 5, where 1 indicates the strongest prediction. RC is a measure of the size of the difference ('diff') between the highest (winning) and the second highest output scores. There are 5 reliability classes, defined as follows:<br>1 : diff > 0.800<br>2 : 0.800 > diff > 0.600<br>3 : 0.600 > diff > 0.400<br>4 : 0.400 > diff > 0.200<br>5 : 0.200 > diff<br>Thus, the lower the value of RC the safer the prediction.
**TPlen**	Predicted presequence length; it appears only when TargetP was asked to perform cleavage site predictions

(see instructions).

The analysis of FNDC5 protein sequence by PeptideCutter (ExPASy: http://web.expasy.org/peptide_cutter/) shows the following cleaving sites:

These enzymes cleave the sequence:

Name of enzyme	No. of cleavages	Positions of cleavage sites
Arg-C proteinase	11	11 15 40 72 75 85 123 143 148 171 206
Asp-N endopeptidase	9	28 51 55 69 90 94 133 173 177
Asp-N endopeptidase + N-terminal Glu	25	28 51 54 55 56 69 78 90 92 93 94 96 114 123 125 133 134 139 150 173 177 179 185 192 197
BNPS-Skatole	5	8 17 51 90 162
CNBr	6	1 73 128 138 141 161
Chymotrypsin-high specificity (C-term to [FYW], not before P)	12	17 23 51 62 76 90 98 119 160 162 169 173
Chymotrypsin-low specificity (C-term to [FYWML], not before P)	36	14 16 17 18 23 25 41 42 51 54 62 73 74 76 89 90 92 98 101 118 119 128 138 141 147 153 159 160 161 162 168 169 173 199 204 205
Clostripain	11	11 15 40 72 75 85 123 143 148 171 206
Formic acid	9	29 52 56 70 91 95 134 174 178
Glutamyl endopeptidase	16	55 57 79 93 94 97 115 124 126 135 140 151 180 186 193 198
Iodosobenzoic acid	5	8 17 51 90 162
LysC	13	43 68 69 120 127 131 133 139 177 185 187 189 208
LysN	13	42 67 68 119 126 130 132 138 176 184 186 188 207
NTCB (2-nitro-5-thiocyanobenzoic acid)	5	5 19 21 86 169
Pepsin (pH1.3)	33	14 16 18 22 23 24 25 41 53 54 61 62 73 76 88 89 91 92 118 119 146 147 152 153 158 159 160 167 168 169 203 204 205
Pepsin (pH>2)	41	14 16 18 22 23 24 25 41 50 51 53 54 61 62 73 76 88 89 90 91 92 97 98 118 119 146 147 152 153 158 159 160 161 162 167 168 169 172 203 204 205
Proteinase K	106	7 8 12 13 14 16 17 18 21 23 24 25 26 28 33 35 37 38 39 42 44 47 48 49 51 53 54 55 57 58 59 60 62 63 64 71 74 76 77 79 80 82 83 84 88 89 90 92 93 94 96 97 98 99 100 102 104 105 107 113 115 117 118 119 121 124 125 126 129 135 136 137 140 147 149 151 152 153 154 155 156 157 158 159 160 162 163 165 166 167 168 169 173 175 176 180 186 188 191 193 194 196 198 204 205 209
Staphylococcal peptidase I	15	55 57 79 93 97 115 124 126 135 140 151 180 186 193 198
Thermolysin	66	6 11 12 13 15 17 20 22 23 24 25 27 34 36 38 41 43 46 47 48 53 58 59 61 62 63 72 73 75 76 87 88 98 99 101 103 104 106 112 116 117 118 127 128 137 146 152 153 154 155 156 157 158 159 160 162 164 165 166 167 168 175 190 203 204 208
Trypsin	24	11 15 40 43 68 69 72 75 85 120 123 127 131 133 139 143 148 171 177 185 187 189 206 208

These chosen enzymes do not cut:

Additionally, unconventional secretion of this protein could be also considered
Zhang and Schekman, Science 2013; Butler and Overall 2009 Nat Rev Drug Discov 2009

Butler GS, Overall CM. Proteomic identification of multitasking proteins in unexpected locations complicates drug targeting.;8:935-48.

Zhang M1, Schekman R. Cell biology. Unconventional secretion, unconventional solutions. Science. 2013 May 3;340(6132):559-61.

Methods and Results of Mass Spectrometry Assay.

FNDC5 immunoprecipitation :

1 mg of total protein was incubated with 2 µg of FNDC5 monoclonal antibody (catalog ab174833, Abcam, Cambridge, UK) overnight at 4ºC, followed by addition of 20 µL of 50%

protein A/G-agarose beads (Santa Cruz Biotechnology) for 2 h at 4ºC. After incubation, beads were washed three times with RIPA buffer and three times with H_2Omq. The pellet was loaded in a 10% SDS-PAGE gel and the bands were visualized using coomassie blue staining. Two bands (25 and 15 kDa) were selected to perform the FNDC5 identification assays by two different proteomics approaches: LC-MALDI/TOF assay or using higher sensitive equipment, 1D-nano LC ESI-MSMS.

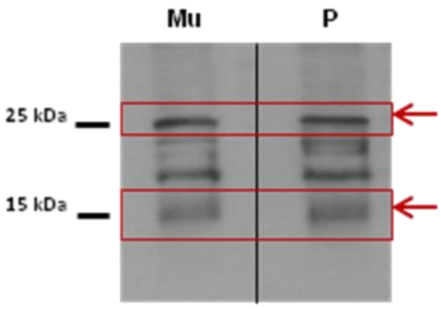

In-gel protein digestion and sample preparation

Bands of interest from 1D gels were excised manually, deposited in 96-well plates and processed automatically in a Proteineer DP (Bruker Daltonics, Bremen, Germany) or subjected to a manual digestion. The digestion protocol used was based on Schevchenko et al. [1] with minor variations: gel plugs were washed firstly with 50 mM ammonium bicarbonate and secondly with ACN prior to reduction with 10 mM DTT in 25 mM ammonium bicarbonate solution, and alkylation was carried out with 55 mM IAA in 50 mM ammonium bicarbonate solution. Gel pieces were then rinsed firstly with 50 mM ammonium bicarbonate and secondly with ACN, and then were dried under a stream of nitrogen. Proteomics Grade Trypsin (Sigma Aldrich), at a final concentration of 16 ng/µl in 25% ACN/50 mM ammonium bicarbonate solution, was added and the digestion took place at 37°C for 4 h. The reaction was stopped by adding 50%ACN/0.5%TFA for peptide extraction. The tryptic eluted peptides were dried by speed-vacuum centrifugation and storaged at -20°C for further analysis.

Protein Identification by **Liquid chromatography and 4800 MALDI-TOF mass spectrometer analysis**

Separation of the resulting tryptic peptide mixtures was performed by nanoscale reversed-phase LC-MS/MS. The nanoLC Ultra 1D plus*, (EKsigent,* ABSciex *Boston, USA)* was coupled to a MALDI-spotter (Eksigent, ABSciex). Peptides mixtures were re-dissolved in 0.1% formic acid, 2% ACN and injected into the trapping column (ChromXP nanoLC Trap column 350 µm id x 0.5 mm, ChromXP C18 3 µm 120Å, ABSciex) at a flow rate of 10 µl/min (0.1% formic acid 2% ACN). After 15 min the trapped peptides were separated in a nanocolumn (ChromXP nanoLC column 75 µm id x 15 cm, ChromXP C18 3um 120Å, ABSciex) at a flow rate of 300 nl/min in a linear gradient elution from 95% A (0.1% formic acid 2% ACN) to 60% B (90% ACN, 0.1% FA) in 30 min followed by an increase up to 95% B in 5 min. The eluting peptides were mixed with a matrix solution, consisting of 3 mg alpha-cyano-4-hydroxycinnamic acid (α-CHCA) dissolved in 1 mL of 50% ACN in 0.1% trifluoroacetic acid, and 10 fmol/µl angiotensin (as internal standard) and deposited, onto a Opti-TOF LC/MALDI insert (ABSciex) with a speed of one spot per 12 seconds.

Mass spectrometry analysis was made using a 4800 MALDI-TOF/TOF analyzer (ABSciex). MS spectra were acquired in reflector positive-ion mode with a Nd:YAG, 355 nm wavelength laser, averaging 1000 laser shots and using 1296.685 peaks of angiotensin I (Sigma) as internal calibration. All MS/MS spectra were performed by selecting the precursors with a relative resolution of 300 (FWHM) and metastable suppression.

Peptide and protein identification were performed using the Protein Pilot software vs 4.0.80.85 (ABSciex) with Paragon Algorithm. MS/MS data was searched against the UniProt/Swiss-Prot database of protein sequences (release 2015-2; Swiss-Prot, Geneva, Switzerland). Searches were restricted to Rattus Novergicus taxonomy allowing carbamidomethyl cysteine as a fixed modification and oxidized methionine as variable modification. Both the precursor mass tolerance and the MS/MS tolerance were set at 30 ppm and 0.35 Da, respectively, allowing 1 missed tryptic cleavage site. Only proteins with a threshold >95% confidence (>1.3 unused score) were considered as positive hits.\

Liquid chromatography and triple TOF 5600 mass spectrometer analysis

Digested peptides of each sample was subjected to 1D-nano LC ESI-MSMS analysis using a nano liquid chromatography system (Eksigent Technologies nanoLC Ultra 1D plus, AB SCIEX, Foster City, CA) coupled to high speed Triple TOF 5600 mass spectrometer (AB SCIEX , Foster City, CA) with a Nanospray III source. The analytical column used was a silica-based reversed phase column C18 ChromXP 75 µm × 15 cm, 3 µm particle size and 120 Å pore size (Eksigent Technologies, AB SCIEX, Foster City, CA). The trap column was a C18 ChromXP (Eksigent Technologies, AB SCIEX, Foster City, CA), 3 µm particle

diameter, 120 Å pore size, switched on-line with the analytical column. The loading pump delivered a solution of 0.1% formic acid in water at 2 µl/min. The nano-pump provided a flow-rate of 250 nl/min and was operated under gradient elution conditions, using 0.1% formic acid in water as mobile phase A, and 0.1% formic acid in acetonitrile as mobile phase B. Peptides were separated using a 100 minutes gradient ranging from 2% to 90% mobile phase B (mobile phase A: 2% acetonitrile, 0.1% formic acid; mobile phase B: 100% acetonitrile, 0.1% formic acid). Injection volume was 5 µl.

Data acquisition was performed with a TripleTOF 5600 System (AB SCIEX, Foster City, CA). To detect lower abundance proteins such as FNDC5 protein, data was acquired using pseudo-MRM approach -an adaptation of the selected reaction monitoring (SRM) scanning mode- that consists of targeted acquisition of product ion spectra associated to unique and specific peptides from these proteins.

All data was acquired using an ionspray voltage floating (ISVF) 2300 V, curtain gas (CUR) 35, interface heater temperature (IHT) 150 and ion source gas 1 (GS1) 15 with Analyst TF 1.7 software (AB SCIEX, USA). For pseudo-MRM parameters, 0.25 s MS survey scan in the mass range of 350–1250 Da were followed by 32 MS/MS scans of 100ms in the mass range of 100–1800 (total cycle time: 3.8 s). Switching criteria were set to ions greater than mass to charge ratio (m/z) 350 and smaller than m/z 1250 with charge state of 2–5 and an abundance threshold of more than 90 counts (cps). Former target ions were excluded for 20 s.

MS and MS/MS data obtained for individual samples were processed using Analyst® TF 1.7 Software (AB SCIEX, USA). Raw data file conversion tools generated mgf files which were also searched against the Rodentia UniProtKB/SwissProt database using the Mascot Server v. 2.5.0 (Matrix Science, London, UK). Search parameters were set as follows: carbamidomethylation of cysteines as fixed and oxidized methionines as variable modification. Peptide mass tolerance was set to 25 ppm and 0.05 Da for fragment masses, also 2 missed cleavages were allowed.

[1] Schevchenko, A., Wilm, M., Vorm, O., Mann, M., Mass spectrometric sequencing of proteins from silver stained polyacrylamide gels. Anal. Chem. 68: 850-858, 1996.

Results

We were not able to identify FNDC5 protein in either proteomic approaches. In the first assay we tried to identify FNDC5 using the commercial FNDC5 peptide as sample; however the LC-MALDI technology was not able to identify this protein. Therefore, we hypothesize that MALDI ionization approach may not be sensitive for FNDC5 detection. In order to improve the identification assay we performed a high sensitive approach, the triple TOF;

however, we were unable to identify FNDC5. We tried a simple search approach or a RMN approach aimed to search the specific peptides obtained in the Silico tryptic digestion. As show in the attachment data, again we did not find FNDC5 in any of these approaches. We believe that the majority of the tryptic peptides obtained by the trypsin digestion might not be efficiently ionizated for its identification.

Supporting this hypothesis, there was only one previous work of Lee et al., detecting an irisin peptide by mass spectrometry study and determined that the 25 kDa band found in human serum samples represents FNDC5 or FNDC5 fragments. Very recently, FNDC5 was identified by mass spectrometry in human plasma in a very interesting article published in Cell metabolism journal by Spiegelman group by using LTQ Orbitrap Elite in an AQUA label samples. Unfortunately these advanced methodology and equipment are not available for our group. However, until now nobody has published data regarding mass spectrometry identification of FNDC5 in rat samples.

Mascot Search Results

```
User                     : proteomics.masas
Email                    : proteomics.masas@cnb.csic.es
Search title             : Susana Bravo_Rodentia_20150603
MS data file             : C:\Users\proteo\Desktop\MGF_Triple Tof\Susana Bravo\20150730\20150729_03_PseudoMRM_25kDa.mgf
Database                 : _UKBsp_all sprot_20150210 (547599 sequences; 195014757 residues)
Timestamp                : 30 Jul 2015 at 11:38:09 GMT
Enzyme                   : Trypsin/P
Fixed modifications      : Carbamidomethyl (C)
Variable modifications   : Oxidation (M)
Mass values              : Monoisotopic
Protein Mass             : Unrestricted
Peptide Mass Tolerance   : ± 25 ppm (# 13C = 1)
Fragment Mass Tolerance: ± 0.05 Da
Max Missed Cleavages     : 2
Instrument type          : ESI-QUAD-TOF
Number of queries        : 37020
Protein hits             : Q99P74 Ras-related protein Rab-27B OS=Rattus norvegicus GN=Rab27b PE=2 SV=3
```

Select Summary Report

Format As | Select Summary (protein hits) | Help

Significance threshold p< 0.05 Max. number of hits AUTO

Standard scoring ◉ MudPIT scoring ○ Ions score or expect cut-off 20 Show sub-sets 0

Show pop-ups ◉ Suppress pop-ups ○ Require bold red ☑

Preferred taxonomy All entries

Re-Search ◉ All queries ○ Unassigned ○ Below homology threshold ○ Below identity threshold

1. Q99P74 **Mass:** 24832 **Score:** 48 **Matches:** 2(2) **Sequences:** 1(1) **emPAI:** 0.16

Ras-related protein Rab-27B OS=Rattus norvegicus GN=Rab27b PE=2 SV=3

Query	Observed	Mr(expt)	Mr(calc)	ppm	Miss	Score	Expect	Rank	Unique	Peptide
12987	415.72	829.43	829.43	0.02	0	48	0.029	1	U	**K.ADLLDQR.E** 12672

Mascot: http://www.matrixscience.com/

```
                          Carbamidomethyl (C)
Variable modifications  : Oxidation (M)
Mass values             : Monoisotopic
Protein Mass            : Unrestricted
Peptide Mass Tolerance  : ± 25 ppm (# 13C = 1)
Fragment Mass Tolerance : ± 0.05 Da
Max Missed Cleavages    : 2
Instrument type         : ESI-QUAD-TOF
Number of queries       : 37025
Protein hits            : P31944 Caspase-14 OS=Homo sapiens GN=CASP14 PE=1 SV=2
```

Select Summary Report

Format As | Select Summary (protein hits) | Help

Significance threshold p< 0.05 | Max. number of hits AUTO

Standard scoring ◉ MudPIT scoring ○ Ions score or expect cut-off 20 | Show sub-sets 0

Show pop-ups ◉ Suppress pop-ups ○ | Require bold red ☑

Preferred taxonomy All entries

Re-Search ◉ All queries ○ Unassigned ○ Below homology threshold ○ Below identity threshold

1. P31944 **Mass:** 27947 **Score:** 59 **Matches:** 2(1) **Sequences:** 1(1) **emPAI:** 0.14

Caspase-14 OS=Homo sapiens GN=CASP14 PE=1 SV=2

Query	Observed	Mr(expt)	Mr(calc)	ppm	Miss	Score	Expect	Rank	Unique	Peptide
17393	482.74	963.47	963.48	-8.15	0	59	0.0021	1	U	K.FQQAIDSR.E 17296

Mascot: http://www.matrixscience.com/

Mascot Search Results

User : **proteomics.masas**
Email : **proteomics.masas@cnb.csic.es**
Search title : **Susana Bravo_Rodentia_20150603**
Database : **_UKBsp_all sprot_20150210 (547599 sequences; 195014757 residues)**
Taxonomy : **Rattus (7932 sequences)**
Timestamp : **3 Jun 2015 at 09:24:06 GMT**
Enzyme : **Trypsin/P**
Fixed modifications : Carbamidomethyl (C)
Variable modifications : Oxidation (M)
Mass values : **Monoisotopic**
Protein Mass : **Unrestricted**
Peptide Mass Tolerance : **± 25 ppm**
Fragment Mass Tolerance: **± 0.05 Da**
Max Missed Cleavages : **1**
Instrument type : **ESI-QUAD-TOF**
Number of queries : **3693**
Protein hits :

P08010 Glutathione S-transferase Mu 2 OS=Rattus norvegicus GN=Gstm2 PE=1 SV=2
P04905 Glutathione S-transferase Mu 1 OS=Rattus norvegicus GN=Gstm1 PE=1 SV=2
P48500 Triosephosphate isomerase OS=Rattus norvegicus GN=Tpi1 PE=1 SV=2
P60711 Actin, cytoplasmic 1 OS=Rattus norvegicus GN=Actb PE=1 SV=1
P04906 Glutathione S-transferase P OS=Rattus norvegicus GN=Gstp1 PE=1 SV=2
P67779 Prohibitin OS=Rattus norvegicus GN=Phb PE=1 SV=1
P27139 Carbonic anhydrase 2 OS=Rattus norvegicus GN=Ca2 PE=1 SV=2
P63102 14-3-3 protein zeta/delta OS=Rattus norvegicus GN=Ywhaz PE=1 SV=1
P14604 Enoyl-CoA hydratase, mitochondrial OS=Rattus norvegicus GN=Echs1 PE=1 SV=1
Q09073 ADP/ATP translocase 2 OS=Rattus norvegicus GN=Slc25a5 PE=1 SV=3
P08009 Glutathione S-transferase Yb-3 OS=Rattus norvegicus GN=Gstm3 PE=1 SV=2
Q68FU3 Electron transfer flavoprotein subunit beta OS=Rattus norvegicus GN=Etfb PE=2 SV=3
Q63279 Keratin, type I cytoskeletal 19 OS=Rattus norvegicus GN=Krt19 PE=1 SV=2
Q10758 Keratin, type II cytoskeletal 8 OS=Rattus norvegicus GN=Krt8 PE=1 SV=3
P62703 40S ribosomal protein S4, X isoform OS=Rattus norvegicus GN=Rps4x PE=2 SV=2
P21913 Succinate dehydrogenase [ubiquinone] iron-sulfur subunit, mitochondrial OS=Rattus norvegicus GN=Sdhb P[illegible]
P01836 Ig kappa chain C region, A allele OS=Rattus norvegicus PE=1 SV=1
P62260 14-3-3 protein epsilon OS=Rattus norvegicus GN=Ywhae PE=1 SV=1
P41123 60S ribosomal protein L13 OS=Rattus norvegicus GN=Rpl13 PE=1 SV=2
P15999 ATP synthase subunit alpha, mitochondrial OS=Rattus norvegicus GN=Atp5a1 PE=1 SV=2
Q6IMF3 Keratin, type II cytoskeletal 1 OS=Rattus norvegicus GN=Krt1 PE=2 SV=1
P00406 Cytochrome c oxidase subunit 2 OS=Rattus norvegicus GN=Mtco2 PE=2 SV=2
P38983 40S ribosomal protein SA OS=Rattus norvegicus GN=Rpsa PE=1 SV=3
Q6IFW6 Keratin, type I cytoskeletal 10 OS=Rattus norvegicus GN=Krt10 PE=3 SV=1
Q05962 ADP/ATP translocase 1 OS=Rattus norvegicus GN=Slc25a4 PE=1 SV=3
Q6P6Q2 Keratin, type II cytoskeletal 5 OS=Rattus norvegicus GN=Krt5 PE=1 SV=1
A7VJC2 Heterogeneous nuclear ribonucleoproteins A2/B1 OS=Rattus norvegicus GN=Hnrnpa2b1 PE=1 SV=1
P10719 ATP synthase subunit beta, mitochondrial OS=Rattus norvegicus GN=Atp5b PE=1 SV=2
Q6IFV3 Keratin, type I cytoskeletal 15 OS=Rattus norvegicus GN=Krt15 PE=1 SV=1
P61983 14-3-3 protein gamma OS=Rattus norvegicus GN=Ywhag PE=1 SV=2
P68255 14-3-3 protein theta OS=Rattus norvegicus GN=Ywhaq PE=1 SV=1
P35435 ATP synthase subunit gamma, mitochondrial OS=Rattus norvegicus GN=Atp5c1 PE=1 SV=2
P25113 Phosphoglycerate mutase 1 OS=Rattus norvegicus GN=Pgam1 PE=1 SV=4
P19234 NADH dehydrogenase [ubiquinone] flavoprotein 2, mitochondrial OS=Rattus norvegicus GN=Ndufv2 PE=1 SV=2
P56571 ES1 protein homolog, mitochondrial OS=Rattus norvegicus PE=1 SV=2
P29410 Adenylate kinase 2, mitochondrial OS=Rattus norvegicus GN=Ak2 PE=2 SV=2
P84100 60S ribosomal protein L19 OS=Rattus norvegicus GN=Rpl19 PE=1 SV=1
Q4FZU2 Keratin, type II cytoskeletal 6A OS=Rattus norvegicus GN=Krt6a PE=1 SV=1
P20767 Ig lambda-2 chain C region OS=Rattus norvegicus PE=4 SV=1
Q99P74 Ras-related protein Rab-27B OS=Rattus norvegicus GN=Rab27b PE=2 SV=3
P35213 14-3-3 protein beta/alpha OS=Rattus norvegicus GN=Ywhab PE=1 SV=3
P32551 Cytochrome b-c1 complex subunit 2, mitochondrial OS=Rattus norvegicus GN=Uqcrc2 PE=1 SV=2
P49242 40S ribosomal protein S3a OS=Rattus norvegicus GN=Rps3a PE=1 SV=2
Q5BJY9 Keratin, type I cytoskeletal 18 OS=Rattus norvegicus GN=Krt18 PE=1 SV=3
P23965 Enoyl-CoA delta isomerase 1, mitochondrial OS=Rattus norvegicus GN=Eci1 PE=1 SV=1
P04904 Glutathione S-transferase alpha-3 OS=Rattus norvegicus GN=Gsta3 PE=1 SV=3
P01946 Hemoglobin subunit alpha-1/2 OS=Rattus norvegicus GN=Hba1 PE=1 SV=3
Q6IG03 Keratin, type II cytoskeletal 73 OS=Rattus norvegicus GN=Krt73 PE=1 SV=1
P04692 Tropomyosin alpha-1 chain OS=Rattus norvegicus GN=Tpm1 PE=1 SV=3
P62907 60S ribosomal protein L10a OS=Rattus norvegicus GN=Rpl10a PE=1 SV=2
Q9Z270 Vesicle-associated membrane protein-associated protein A OS=Rattus norvegicus GN=Vapa PE=1 SV=3
P29411 GTP:AMP phosphotransferase AK3, mitochondrial OS=Rattus norvegicus GN=Ak3 PE=2 SV=2
P34058 Heat shock protein HSP 90-beta OS=Rattus norvegicus GN=Hsp90ab1 PE=1 SV=4
P82995 Heat shock protein HSP 90-alpha OS=Rattus norvegicus GN=Hsp90aa1 PE=1 SV=3
P47727 Carbonyl reductase [NADPH] 1 OS=Rattus norvegicus GN=Cbr1 PE=1 SV=2
P63039 60 kDa heat shock protein, mitochondrial OS=Rattus norvegicus GN=Hspd1 PE=1 SV=1
P68511 14-3-3 protein eta OS=Rattus norvegicus GN=Ywhah PE=1 SV=2
P12001 60S ribosomal protein L18 OS=Rattus norvegicus GN=Rpl18 PE=2 SV=2
O70351 3-hydroxyacyl-CoA dehydrogenase type-2 OS=Rattus norvegicus GN=Hsd17b10 PE=1 SV=3
P85834 Elongation factor Tu, mitochondrial OS=Rattus norvegicus GN=Tufm PE=1 SV=1
Q9Z1B2 Glutathione S-transferase Mu 5 OS=Rattus norvegicus GN=Gstm5 PE=1 SV=3
P16036 Phosphate carrier protein, mitochondrial OS=Rattus norvegicus GN=Slc25a3 PE=1 SV=1
Q6AYQ8 Acylpyruvase FAHD1, mitochondrial OS=Rattus norvegicus GN=Fahd1 PE=2 SV=1
P02770 Serum albumin OS=Rattus norvegicus GN=Alb PE=1 SV=2
Q63610 Tropomyosin alpha-3 chain OS=Rattus norvegicus GN=Tpm3 PE=1 SV=2
P09626 Potassium-transporting ATPase alpha chain 1 OS=Rattus norvegicus GN=Atp4a PE=2 SV=3
P70580 Membrane-associated progesterone receptor component 1 OS=Rattus norvegicus GN=Pgrmc1 PE=1 SV=3
O35760 Isopentenyl-diphosphate Delta-isomerase 1 OS=Rattus norvegicus GN=Idi1 PE=2 SV=2
Q63507 60S ribosomal protein L14 OS=Rattus norvegicus GN=Rpl14 PE=1 SV=3
P21670 Proteasome subunit alpha type-4 OS=Rattus norvegicus GN=Psma4 PE=1 SV=1
Q29TV8 Gastrokine-2 OS=Rattus norvegicus GN=Gkn2 PE=2 SV=1
P08699 Galectin-3 OS=Rattus norvegicus GN=Lgals3 PE=1 SV=4
Q66H80 Coatomer subunit delta OS=Rattus norvegicus GN=Arcn1 PE=2 SV=1
P34064 Proteasome subunit alpha type-5 OS=Rattus norvegicus GN=Psma5 PE=2 SV=1
Q8K4G6 O-acetyl-ADP-ribose deacetylase MACROD1 (Fragment) OS=Rattus norvegicus GN=Macrod1 PE=2 SV=2
P18422 Proteasome subunit alpha type-3 OS=Rattus norvegicus GN=Psma3 PE=1 SV=3
Q6URK4 Heterogeneous nuclear ribonucleoprotein A3 OS=Rattus norvegicus GN=Hnrnpa3 PE=1 SV=1
P83732 60S ribosomal protein L24 OS=Rattus norvegicus GN=Rpl24 PE=2 SV=1
Q5XI73 Rho GDP-dissociation inhibitor 1 OS=Rattus norvegicus GN=Arhgdia PE=1 SV=1
P11348 Dihydropteridine reductase OS=Rattus norvegicus GN=Qdpr PE=1 SV=1
P22062 Protein-L-isoaspartate(D-aspartate) O-methyltransferase OS=Rattus norvegicus GN=Pcmt1 PE=1 SV=2
P19511 ATP synthase F(0) complex subunit B1, mitochondrial OS=Rattus norvegicus GN=Atp5f1 PE=1 SV=1
P62630 Elongation factor 1-alpha 1 OS=Rattus norvegicus GN=Eef1a1 PE=1 SV=1
P61314 60S ribosomal protein L15 OS=Rattus norvegicus GN=Rpl15 PE=1 SV=2
P60901 Proteasome subunit alpha type-6 OS=Rattus norvegicus GN=Psma6 PE=1 SV=1
Q4KM65 Cleavage and polyadenylation specificity factor subunit 5 OS=Rattus norvegicus GN=Nudt21 PE=2 SV=1

Q00715 Histone H2B type 1 OS=Rattus norvegicus PE=1 SV=2
Q6MG61 Chloride intracellular channel protein 1 OS=Rattus norvegicus GN=Clic1 PE=1 SV=1
Q6PDV7 60S ribosomal protein L10 OS=Rattus norvegicus GN=Rpl10 PE=1 SV=3
Q68FT1 Ubiquinone biosynthesis protein COQ9, mitochondrial OS=Rattus norvegicus GN=Coq9 PE=2 SV=2
P52555 Endoplasmic reticulum resident protein 29 OS=Rattus norvegicus GN=Erp29 PE=1 SV=2
Q68G31 Phenazine biosynthesis-like domain-containing protein OS=Rattus norvegicus GN=Pbld PE=2 SV=1
P30713 Glutathione S-transferase theta-2 OS=Rattus norvegicus GN=Gstt2 PE=1 SV=3
P62243 40S ribosomal protein S8 OS=Rattus norvegicus GN=Rps8 PE=1 SV=2
Q5I0K8 28S ribosomal protein S7, mitochondrial OS=Rattus norvegicus GN=Mrps7 PE=2 SV=2
Q7TP52 Carboxymethylenebutenolidase homolog OS=Rattus norvegicus GN=Cmbl PE=2 SV=1
Q9Z0V5 Peroxiredoxin-4 OS=Rattus norvegicus GN=Prdx4 PE=2 SV=1
Q63716 Peroxiredoxin-1 OS=Rattus norvegicus GN=Prdx1 PE=1 SV=1
P62824 Ras-related protein Rab-3C OS=Rattus norvegicus GN=Rab3c PE=1 SV=1
P63012 Ras-related protein Rab-3A OS=Rattus norvegicus GN=Rab3a PE=1 SV=1
Q63942 GTP-binding protein Rab-3D OS=Rattus norvegicus GN=Rab3d PE=1 SV=2
P04256 Heterogeneous nuclear ribonucleoprotein A1 OS=Rattus norvegicus GN=Hnrnpa1 PE=1 SV=3
Q5RKI1 Eukaryotic initiation factor 4A-II OS=Rattus norvegicus GN=Eif4a2 PE=1 SV=1
P13803 Electron transfer flavoprotein subunit alpha, mitochondrial OS=Rattus norvegicus GN=Etfa PE=1 SV=4
Q63797 Proteasome activator complex subunit 1 OS=Rattus norvegicus GN=Psme1 PE=2 SV=1
P23640 Ras-related protein Rab-27A OS=Rattus norvegicus GN=Rab27a PE=1 SV=1
Q498R3 DnaJ homolog subfamily C member 10 OS=Rattus norvegicus GN=Dnajc10 PE=2 SV=2
P48004 Proteasome subunit alpha type-7 OS=Rattus norvegicus GN=Psma7 PE=1 SV=1
Q5XI72 Eukaryotic translation initiation factor 4H OS=Rattus norvegicus GN=Eif4h PE=1 SV=1
P02773 Alpha-fetoprotein OS=Rattus norvegicus GN=Afp PE=2 SV=1
P17077 60S ribosomal protein L9 OS=Rattus norvegicus GN=Rpl9 PE=1 SV=1
P38918 Aflatoxin B1 aldehyde reductase member 3 OS=Rattus norvegicus GN=Akr7a3 PE=1 SV=2
Q9JHW0 Proteasome subunit beta type-7 OS=Rattus norvegicus GN=Psmb7 PE=1 SV=1
Q5XIF6 Tubulin alpha-4A chain OS=Rattus norvegicus GN=Tuba4a PE=2 SV=1
P62804 Histone H4 OS=Rattus norvegicus GN=Hist1h4b PE=1 SV=2
Q810U0 Coiled-coil domain-containing protein 50 OS=Rattus norvegicus GN=Ccdc50 PE=2 SV=1
Q63798 Proteasome activator complex subunit 2 OS=Rattus norvegicus GN=Psme2 PE=2 SV=3
Q3KRE8 Tubulin beta-2B chain OS=Rattus norvegicus GN=Tubb2b PE=1 SV=1
P17136 Small nuclear ribonucleoprotein-associated protein B (Fragment) OS=Rattus norvegicus GN=Snrpb PE=2 SV=
Q9QWN8 Spectrin beta chain, non-erythrocytic 2 OS=Rattus norvegicus GN=Sptbn2 PE=1 SV=2
Q9Z0W7 Chloride intracellular channel protein 4 OS=Rattus norvegicus GN=Clic4 PE=1 SV=3
O35952 Hydroxyacylglutathione hydrolase, mitochondrial OS=Rattus norvegicus GN=Hagh PE=1 SV=2
O35264 Platelet-activating factor acetylhydrolase IB subunit beta OS=Rattus norvegicus GN=Pafah1b2 PE=1 SV=1
Q5XI32 F-actin-capping protein subunit beta OS=Rattus norvegicus GN=Capzb PE=1 SV=1
B0BN44 Tumor protein p53-inducible protein 13 OS=Rattus norvegicus GN=Tp53i13 PE=2 SV=1
Q4KLH6 Centrosomal protein of 162 kDa OS=Rattus norvegicus GN=Cep162 PE=1 SV=2
Q5U316 Ras-related protein Rab-35 OS=Rattus norvegicus GN=Rab35 PE=1 SV=1
Q6NYB7 Ras-related protein Rab-1A OS=Rattus norvegicus GN=Rab1A PE=1 SV=3
P20294 Ciliary neurotrophic factor OS=Rattus norvegicus GN=Cntf PE=1 SV=1
Q5PPI1 Serine/arginine-rich splicing factor 9 OS=Rattus norvegicus GN=Srsf9 PE=1 SV=1
Q9Z269 Vesicle-associated membrane protein-associated protein B OS=Rattus norvegicus GN=Vapb PE=1 SV=3
O35263 Platelet-activating factor acetylhydrolase IB subunit gamma OS=Rattus norvegicus GN=Pafah1b3 PE=2 SV=1
Q711G3 Isoamyl acetate-hydrolyzing esterase 1 homolog OS=Rattus norvegicus GN=Iah1 PE=2 SV=2
P20759 Ig gamma-1 chain C region OS=Rattus norvegicus PE=1 SV=1
Q6AYK6 Calcyclin-binding protein OS=Rattus norvegicus GN=Cacybp PE=1 SV=1
Q923M1 Mitochondrial peptide methionine sulfoxide reductase OS=Rattus norvegicus GN=Msra PE=1 SV=1
P07896 Peroxisomal bifunctional enzyme OS=Rattus norvegicus GN=Ehhadh PE=1 SV=2
O35244 Peroxiredoxin-6 OS=Rattus norvegicus GN=Prdx6 PE=1 SV=3
Q9JKS6 Protein piccolo OS=Rattus norvegicus GN=Pclo PE=1 SV=1
Q4V8Q1 Calpain 11 OS=Rattus norvegicus GN=Capn11 PE=2 SV=2
P24268 Cathepsin D OS=Rattus norvegicus GN=Ctsd PE=1 SV=1
Q3KR99 Protein Spindly OS=Rattus norvegicus GN=Spdl1 PE=2 SV=1
P16086 Spectrin alpha chain, non-erythrocytic 1 OS=Rattus norvegicus GN=Sptan1 PE=1 SV=2
P68101 Eukaryotic translation initiation factor 2 subunit 1 OS=Rattus norvegicus GN=Eif2s1 PE=1 SV=2
Q6AY09 Heterogeneous nuclear ribonucleoprotein H2 OS=Rattus norvegicus GN=Hnrnph2 PE=1 SV=1
P97532 3-mercaptopyruvate sulfurtransferase OS=Rattus norvegicus GN=Mpst PE=1 SV=3
Q5RJR8 Leucine-rich repeat-containing protein 59 OS=Rattus norvegicus GN=Lrrc59 PE=1 SV=1
P27605 Hypoxanthine-guanine phosphoribosyltransferase OS=Rattus norvegicus GN=Hprt1 PE=1 SV=1
P11232 Thioredoxin OS=Rattus norvegicus GN=Txn PE=1 SV=2
Q505J8 Phenylalanine--tRNA ligase alpha subunit OS=Rattus norvegicus GN=Farsa PE=1 SV=1
Q32PZ3 Protein unc-45 homolog A OS=Rattus norvegicus GN=Unc45a PE=2 SV=1
Q5I0E7 Transmembrane emp24 domain-containing protein 9 OS=Rattus norvegicus GN=Tmed9 PE=1 SV=1
Q64595 cGMP-dependent protein kinase 2 OS=Rattus norvegicus GN=Prkg2 PE=1 SV=1
Q4G069 Regulator of microtubule dynamics protein 1 OS=Rattus norvegicus GN=Rmdn1 PE=2 SV=1
P21575 Dynamin-1 OS=Rattus norvegicus GN=Dnm1 PE=1 SV=2
Q3KR73 KAT8 regulatory NSL complex subunit 3 OS=Rattus norvegicus GN=Kansl3 PE=2 SV=1
O54939 Testosterone 17-beta-dehydrogenase 3 OS=Rattus norvegicus GN=Hsd17b3 PE=2 SV=1
O88761 26S proteasome non-ATPase regulatory subunit 1 OS=Rattus norvegicus GN=Psmd1 PE=2 SV=1
Q6AXQ7 IQ and AAA domain-containing protein 1-like OS=Rattus norvegicus GN=Iqca1p1 PE=2 SV=2
P41739 Aryl hydrocarbon receptor nuclear translocator OS=Rattus norvegicus GN=Arnt PE=1 SV=2
P08689 Sex hormone-binding globulin OS=Rattus norvegicus GN=Shbg PE=1 SV=1
Q5EBA1 ATP-dependent RNA helicase SUPV3L1, mitochondrial OS=Rattus norvegicus GN=Supv3l1 PE=2 SV=1
Q9Z0T0 Thiopurine S-methyltransferase OS=Rattus norvegicus GN=Tpmt PE=2 SV=1
F1M649 NLR family CARD domain-containing protein 4 OS=Rattus norvegicus GN=Nlrc4 PE=3 SV=2
Q5QE80 Aldehyde oxidase 3 OS=Rattus norvegicus GN=Aox3 PE=1 SV=1
D3ZBP4 Protein-methionine sulfoxide oxidase MICAL1 OS=Rattus norvegicus GN=Mical1 PE=3 SV=1
Q00566 Methyl-CpG-binding protein 2 OS=Rattus norvegicus GN=Mecp2 PE=1 SV=1
Q5U2Q4 Mono [ADP-ribose] polymerase PARP16 OS=Rattus norvegicus GN=Parp16 PE=2 SV=1
F1MA98 Nucleoprotein TPR OS=Rattus norvegicus GN=Tpr PE=1 SV=1
Q9ERD9 Indoleamine 2,3-dioxygenase 1 OS=Rattus norvegicus GN=Ido1 PE=2 SV=1
Q05030 Platelet-derived growth factor receptor beta OS=Rattus norvegicus GN=Pdgfrb PE=2 SV=2
Q63517 Centromere protein I OS=Rattus norvegicus GN=Cenpi PE=2 SV=1
Q8VIL3 ZW10 interactor OS=Rattus norvegicus GN=Zwint PE=1 SV=1
Q498M5 Putative E3 ubiquitin-protein ligase SH3RF2 OS=Rattus norvegicus GN=Sh3rf2 PE=2 SV=1
P13383 Nucleolin OS=Rattus norvegicus GN=Ncl PE=1 SV=3

Select Summary Report

Format As | Select Summary (protein hits) | Help

Significance threshold p< 0.05 | Max. number of hits AUTO

Standard scoring ◉ MudPIT scoring ○ Ions score or expect cut-off 20 | Show sub-sets 0

Show pop-ups ◉ Suppress pop-ups ○ | Require bold red ☐

Preferred taxonomy All entries

Re-Search ◉ All queries ○ Unassigned ○ Below homology threshold ○ Below identity threshold

1. P08010 **Mass:** 25857 **Score:** 988 **Matches:** 21(21) **Sequences:** 17(17) **emPAI:** 19.53
Glutathione S-transferase Mu 2 OS=Rattus norvegicus GN=Gstm2 PE=1 SV=2

Query	Observed	Mr(expt)	Mr(calc)	ppm	Miss	Score	Expect	Rank	Unique	Peptide
81	378.68	755.35	755.35	1.13	0	31	0.0027	1		K.ISDYMK.S
388	439.22	876.43	876.43	-3.73	0	35	0.003	1	U	R.SQWLSEK.F
590	474.24	946.47	946.47	5.78	0	41	0.00073	1	U	K.QPWFAGNK.I
628	478.76	955.50	955.50	2.05	0	40	0.00056	1		K.LYSEFLGK.Q
791	508.29	1014.57	1014.58	-7.77	0	71	5.8e-007	1		K.ITQSNAILR.Y
901	350.88	1049.62	1049.63	-3.70	1	57	5.4e-006	1	U	R.FLSKPIFAK.M
977	539.27	1076.53	1076.53	-0.90	0	41	0.00066	1		K.CLDAFPNLK.D
1610	634.31	1266.60	1266.61	-2.75	0	88	2.3e-008	1		M.PMTLGYWDIR.G
1665	645.26	1288.50	1288.50	0.90	0	(38)	0.00017	2	U	K.YSMGDAPDYDR.S 1666
1693	434.90	1301.68	1301.69	-4.60	1	42	0.0004	1	U	K.KPEYLEGLPEK.M
1704	653.26	1304.50	1304.50	-1.47	0	76	2.5e-008	1	U	K.YSMGDAPDYDR.S
1887	458.53	1372.56	1372.57	-3.36	0	40	0.00017	1		K.HNLCGETEEER.I 1886
1976	703.83	1405.65	1405.65	1.37	0	87	1e-008	1	U	R.VDVLENQAMDTR.L
2029	478.54	1432.59	1432.59	-2.36	1	49	1.4e-005	1	U	K.KYSMGDAPDYDR.S
2541	812.38	1622.75	1622.74	11.9	0	29	0.0077	1	U	R.LFLEYTDTSYEDK.K
2609	833.42	1664.83	1664.83	-0.68	1	91	5.5e-009	1	U	K.CLDAFPNLKDFVAR.F
2715	576.94	1727.80	1727.80	0.82	0	(41)	0.00037	1	U	R.LQLAMVCYSPDFER.K
2748	872.90	1743.79	1743.80	-1.20	0	59	4.5e-006	1	U	R.LQLAMVCYSPDFER.K
2980	633.67	1897.99	1897.99	-1.57	0	110	5.3e-011	1		K.LGLDFPNLPYLIDGSHK.I

2. P04905 **Mass:** 26068 **Score:** 804 **Matches:** 17(17) **Sequences:** 16(16) **emPAI:** 11.47
Glutathione S-transferase Mu 1 OS=Rattus norvegicus GN=Gstm1 PE=1 SV=2

Query	Observed	Mr(expt)	Mr(calc)	ppm	Miss	Score	Expect	Rank	Unique	Peptide
13	356.69	711.37	711.36	5.33	0	28	0.0086	1	U	K.ISAYMK.S
312	423.73	845.44	845.44	-0.00	0	39	0.0015	1	U	K.LAQWSNK.-
434	445.26	888.50	888.51	-10.30	1	39	0.0011	1	U	K.QKPEFLK.T
628	478.76	955.50	955.50	2.05	0	40	0.00056	1		K.LYSEFLGK.R
678	488.74	975.47	975.49	-19.76	1	30	0.0074	1		K.RPWFAGDK.V
896	525.27	1048.53	1048.53	-3.64	0	30	0.01	1	U	K.ITQSNAIMR.Y
912	528.29	1054.57	1054.57	2.70	0	39	0.0007	1	U	R.YLSTPIFSK.L
977	539.27	1076.53	1076.53	-0.90	0	41	0.00066	1		K.CLDAFPNLK.D
1342	393.22	1176.62	1176.63	-3.50	1	43	0.00035	1	U	R.KITQSNAIMR.Y
1562	624.83	1247.65	1247.65	-1.36	0	29	0.01	1	U	M.PMILGYWNVR.G
1665	645.26	1288.50	1288.50	0.90	0	64	4.2e-007	1	U	R.YAMGDAPDYDR.S 1666
2003	710.33	1418.65	1418.65	0.00	0	87	7.4e-009	1	U	R.ADIVENQVMDNR.M
2481	795.38	1588.76	1588.75	2.88	0	84	2.2e-008	1		R.LLLEYTDSSYEEK.R
2638	560.62	1678.84	1678.85	-5.32	1	65	2.1e-006	1	U	K.CLDAFPNLKDFLAR.F
2820	895.48	1788.94	1788.94	0.55	0	117	1e-011	1	U	K.LGLDFPNLPYLIDGSR.K
3498	863.77	2588.29	2588.28	0.46	0	30	0.0047	1	U	K.VTYVDFLAYDILDQYHIFEPK.C

3. P48500 **Mass:** 27345 **Score:** 775 **Matches:** 15(14) **Sequences:** 11(11) **emPAI:** 4.25
Triosephosphate isomerase OS=Rattus norvegicus GN=Tpi1 PE=1 SV=2

Query	Observed	Mr(expt)	Mr(calc)	ppm	Miss	Score	Expect	Rank	Unique	Peptide
319	425.74	849.46	849.46	-3.55	0	46	0.00022	1	U	K.VVFEQTK.A
1761	663.84	1325.66	1325.66	-2.20	0	96	1.4e-009	1	U	R.IIYGGSVTGATCK.E
1907	690.80	1379.59	1379.59	-1.90	0	81	1.8e-008	1	U	K.CNVSEGVAQCTR.I
2122	731.87	1461.73	1461.73	0.34	0	80	8e-008	1	U	K.CLGELICTLNAAK.L
2138	489.58	1465.71	1465.72	-1.72	0	92	3.3e-009	1	U	K.TATPQQAQEVHEK.L 2135 2136
2360	770.40	1538.78	1538.78	-0.59	0	73	3.3e-007	1	U	K.DLGATWVVLGHSER.R
2506	801.93	1601.85	1601.88	-17.65	0	(55)	1.9e-005	1	U	K.VVLAYEPVWAIGTGK.T
2507	534.96	1601.86	1601.88	-10.94	0	76	1.5e-007	1	U	K.VVLAYEPVWAIGTGK.T
2535	811.41	1620.81	1620.82	-5.95	0	(21)	0.12	1	U	K.VTNGAFTGEISPGMIK.D
2550	407.96	1627.83	1627.83	-3.04	1	44	0.00026	1	U	R.RHIFGESDELIGQK.V
2565	819.41	1636.81	1636.81	-1.87	0	42	0.00047	1	U	K.VTNGAFTGEISPGMIK.D
2929	622.99	1865.96	1865.97	-4.00	0	69	5.8e-007	1	U	K.VNHALSEGLGVIACIGEK.L
3304	1103.55	2205.08	2205.08	0.55	0	76	1.2e-007	1	U	K.LPADTEVVCAPPTAYIDFAR.Q

4. P60711 **Mass:** 42052 **Score:** 607 **Matches:** 13(12) **Sequences:** 12(11) **emPAI:** 2.25
Actin, cytoplasmic 1 OS=Rattus norvegicus GN=Actb PE=1 SV=1

Query	Observed	Mr(expt)	Mr(calc)	ppm	Miss	Score	Expect	Rank	Unique	Peptide
139	398.24	794.46	794.47	-6.63	0	45	0.00012	1	U	K.IIAPPER.K
583	473.28	944.54	944.54	-8.14	0	36	0.0019	1	U	R.AVFPSIVGR.P
732	499.75	997.48	997.48	-1.94	0	(41)	0.00049	1	U	R.DLTDYLMK.I
784	507.75	1013.49	1013.47	11.9	0	43	0.00027	1	U	R.DLTDYLMK.I
1206	566.77	1131.52	1131.52	-1.01	0	42	0.00025	1	U	R.GYSFTTTAER.E
1285	581.31	1160.60	1160.61	-7.77	0	23	0.092	1	U	K.EITALAPSTMK.I
1415	599.76	1197.51	1197.51	-1.89	0	56	5e-006	1	U	K.DSYVGDEAQSK.R
1419	400.24	1197.70	1197.70	1.01	1	33	0.0021	1	U	R.AVFPSIVGRPR.H
1831	452.21	1353.62	1353.62	-0.53	1	82	3.3e-008	1	U	K.DSYVGDEAQSKR.G
2264	758.86	1515.70	1515.70	0.60	0	74	1.7e-007	1	U	K.QEYDESGPSIVHR.K
2821	895.95	1789.89	1789.88	4.26	0	99	7.9e-010	1	U	K.SYELPDGQVITIGNER.F
3060	652.03	1953.06	1953.06	0.48	0	38	0.00059	1	U	R.VAPEEHPVLLTEAPLNPK.A
3633	800.66	3198.60	3198.60	0.80	0	36	0.00068	1	U	R.TTGIVMDSGDGVTHTVPIYEGYALPHAILR.L

Proteins matching the same set of peptides:
P63259 **Mass:** 42108 **Score:** 607 **Matches:** 13(12) **Sequences:** 12(11)
Actin, cytoplasmic 2 OS=Rattus norvegicus GN=Actg1 PE=1 SV=1

5. P04906 **Mass:** 23652 **Score:** 564 **Matches:** 9(9) **Sequences:** 8(8) **emPAI:** 3.78
Glutathione S-transferase P OS=Rattus norvegicus GN=Gstp1 PE=1 SV=2

Query	Observed	Mr(expt)	Mr(calc)	ppm	Miss	Score	Expect	Rank	Unique	Peptide
50	369.21	736.40	736.41	-15.45	0	29	0.0067	1	U	R.SLGLYGK.D

1305	583.80	1165.58	1165.58	-2.38	0	40	0.00074	1	U	K.STCLYGQLPK.F
1825	676.37	1350.73	1350.73	-2.24	0	71	4.2e-007	1	U	M.PPYTIVYFPVR.G
2750	873.42	1744.83	1744.83	-0.18	0	123	3e-012	1	U	K.EAALVDMVNDGVEDLR.C
2777	881.42	1760.82	1760.83	-1.26	0	(110)	4.6e-011	1	U	K.EAALVDMVNDGVEDLR.C
2905	927.97	1853.92	1853.92	1.22	0	114	2e-011	1	U	K.FEDGDLTLYQSNAILR.H
3026	485.00	1935.99	1935.99	-1.19	1	40	0.00048	1	U	K.AFLSSPDHLNRPINGNGK.Q
3249	712.39	2134.16	2134.15	0.79	1	60	2.5e-006	1	U	K.ALPGHLKPFETLLSQNQGGK.A
3264	719.34	2155.01	2155.01	0.91	1	87	7.7e-009	1	U	K.YGTLIYTNYENGKDDYVK.A

6. P67779 **Mass:** 29859 **Score:** 546 **Matches:** 10(10) **Sequences:** 10(10) **emPAI:** 2.98
Prohibitin OS=Rattus norvegicus GN=Phb PE=1 SV=1

Query	Observed	Mr(expt)	Mr(calc)	ppm	Miss	Score	Expect	Rank	Unique	Peptide
509	457.77	913.52	913.52	-1.38	0	33	0.0038	1	U	R.NVPVITGSK.D
811	512.25	1022.48	1022.49	-10.52	0	61	4.1e-006	1	U	K.EFTEAVEAK.Q
928	531.28	1060.54	1060.54	-0.60	0	46	0.0002	1	U	K.AAIISAEGDSK.A
934	531.76	1061.50	1061.50	-0.88	0	42	0.00043	1	U	R.QVSDDLTER.A
1252	575.30	1148.58	1148.58	-1.14	0	66	1.9e-006	1	U	R.FDAGELITQR.E
1458	607.37	1212.73	1212.73	-1.04	0	49	2.7e-005	1	U	R.VLPSITTEILK.S
546	466.28	1395.83	1395.84	-3.03	1	55	2.1e-005	1	U	R.ILFRPVASQLPR.I
2116	730.83	1459.64	1459.65	-2.61	0	84	1e-008	1	U	R.IYTSIGEDYDER.V
2517	536.29	1605.84	1605.84	3.13	1	55	1.7e-005	1	U	R.KLEAAEDIAYQLSR.S
2907	619.01	1854.02	1854.03	-3.80	0	53	1.2e-005	1	U	R.NITYLPAGQSVLLQLPQ.-

7. P27139 **Mass:** 29267 **Score:** 534 **Matches:** 10(10) **Sequences:** 9(9) **emPAI:** 2.54
Carbonic anhydrase 2 OS=Rattus norvegicus GN=Ca2 PE=1 SV=2

Query	Observed	Mr(expt)	Mr(calc)	ppm	Miss	Score	Expect	Rank	Unique	Peptide
651	483.24	964.46	964.46	-1.79	0	53	3.4e-005	1	U	K.EGPLSGSYR.L
734	499.78	997.56	997.56	-0.33	0	72	3.2e-007	1	U	K.IGPASQGLQK.I
776	506.29	1010.58	1010.58	-0.68	0	42	0.00024	1	U	K.ITEALHSIK.T
2465	396.21	1580.81	1580.81	0.45	0	(55)	1.8e-005	1	U	K.YAAELHLVHWNTK.Y
2466	791.41	1580.81	1580.81	1.17	0	88	9.9e-009	1	U	K.YAAELHLVHWNTK.Y
2607	555.26	1662.77	1662.77	2.10	0	87	9.7e-009	1	U	K.EPITVSSEQMSHFR.K
2632	559.99	1676.96	1676.96	-0.27	0	96	3.5e-010	1	U	K.AVQHPDGLAVLGIFLK.I
2686	428.23	1708.90	1708.90	-3.39	1	41	0.00044	1	U	K.KYAAELHLVHWNTK.Y
3496	861.41	2581.21	2581.21	-0.47	0	22	0.02	1	U	K.SIVNNGHSFNVEFDDSQDFAVLK.E
3565	728.61	2910.41	2910.41	-0.32	0	33	0.0016	1	U	R.QSPVDIDTGTAQHDPSLQPLLICYDK.V

8. P63102 **Mass:** 27925 **Score:** 531 **Matches:** 10(9) **Sequences:** 9(8) **emPAI:** 2.76
14-3-3 protein zeta/delta OS=Rattus norvegicus GN=Ywhaz PE=1 SV=1

Query	Observed	Mr(expt)	Mr(calc)	ppm	Miss	Score	Expect	Rank	Unique	Peptide
493	454.27	906.52	906.52	-0.94	0	44	0.00019	1		R.NLLSVAYK.N
1262	576.29	1150.56	1150.55	6.60	0	20	0.068	1	U	R.YLAEVAAGDDK.K
1394	595.33	1188.65	1188.65	-3.18	0	67	1.7e-006	1		K.DSTLIMQLLR.D
1442	603.33	1204.65	1204.65	-1.59	0	(66)	1.9e-006	1		K.DSTLIMQLLR.D
1646	427.22	1278.65	1278.65	0.35	1	76	1.7e-007	1	U	R.YLAEVAAGDDKK.G
1773	444.24	1329.69	1329.69	-0.03	0	52	4.7e-005	1	U	K.FLIPNASQPESK.V
2000	709.86	1417.71	1417.71	0.37	0	68	1.2e-006	1	U	R.DICNDVLSLLEK.F
2384	774.86	1547.71	1547.71	0.31	0	100	4.4e-010	1	U	K.SVTEQGAELSNEER.N
3169	1021.00	2039.98	2039.98	2.24	0	58	7.4e-006	1	U	K.GIVDQSQQAYQEAFEISK.K
3280	723.70	2168.08	2168.07	2.85	1	46	0.00012	1	U	K.GIVDQSQQAYQEAFEISKK.E

9. P14604 **Mass:** 31895 **Score:** 524 **Matches:** 11(11) **Sequences:** 9(9) **emPAI:** 3.15
Enoyl-CoA hydratase, mitochondrial OS=Rattus norvegicus GN=Echs1 PE=1 SV=1

Query	Observed	Mr(expt)	Mr(calc)	ppm	Miss	Score	Expect	Rank	Unique	Peptide
348	432.23	862.45	862.45	-4.43	0	55	3.1e-005	1	U	K.AFAAGADIK.E
781	507.23	1012.45	1012.45	1.02	0	36	0.00074	1	U	R.EGMSAFVEK.R
1116	553.24	1104.46	1104.45	1.18	0	35	0.00035	1	U	R.TFQDCYSGK.F
1753	661.83	1321.65	1321.64	9.33	0	(32)	0.0045	1	U	K.SLAMEMVLTGDR.I
1781	668.33	1334.65	1334.61	23.4	0	29	0.0088	1	U	K.LFYSTFATDDR.R
1788	669.83	1337.64	1337.63	2.64	0	(42)	0.00063	1	U	K.SLAMEMVLTGDR.I
1832	677.82	1353.62	1353.63	-3.49	0	46	0.00016	1	U	K.SLAMEMVLTGDR.I
2022	475.94	1424.80	1424.81	-4.53	1	103	1.6e-010	1	U	K.NSSVGLIQLNRPK.A
2197	497.91	1490.72	1490.72	2.94	1	40	0.00065	1	U	K.LFYSTFATDDRR.E
2352	513.25	1536.73	1536.74	-1.35	1	105	2.3e-010	1	U	K.AFAAGADIKEMQNR.T
3087	659.34	1975.00	1975.00	-0.24	0	75	1.6e-007	1	U	K.IFPVETLVEEAIQCAEK.I

10. Q09073 **Mass:** 33108 **Score:** 497 **Matches:** 11(11) **Sequences:** 10(10) **emPAI:** 2.48
ADP/ATP translocase 2 OS=Rattus norvegicus GN=Slc25a5 PE=1 SV=3

Query	Observed	Mr(expt)	Mr(calc)	ppm	Miss	Score	Expect	Rank	Unique	Peptide
108	387.20	772.38	772.38	-3.64	0	30	0.0052	1		K.GMLPDPK.N
332	428.75	855.48	855.48	-1.23	0	54	2.7e-005	1		K.TAVAPIER.V
333	428.76	855.50	855.49	3.67	0	26	0.011	1		R.GNLANVIR.Y
344	431.23	860.44	860.44	-1.78	0	32	0.0072	1	U	K.GLGDCLVK.I
681	488.78	975.54	975.54	0.13	0	44	0.00027	1	U	K.QIFLGGVDK.R
1171	561.29	1120.57	1120.57	-0.91	0	49	9.6e-005	1	U	K.EQGVLSFWR.G
1472	610.31	1218.60	1218.59	8.43	0	59	9.8e-006	1	U	R.AAYFGIYDTAK.G
1476	610.34	1218.66	1218.66	-2.43	0	73	3.3e-007	1	U	K.DFLAGGVAAAISK.T 1475
1864	455.28	1362.82	1362.83	-9.05	1	67	2.1e-007	1		R.VKLLLQVQHASK.Q
2076	723.87	1445.74	1445.73	0.64	0	65	2.5e-006	1		R.YFPTQALNFAFK.D

11. P08009 **Mass:** 25835 **Score:** 495 **Matches:** 9(9) **Sequences:** 8(8) **emPAI:** 2.57
Glutathione S-transferase Yb-3 OS=Rattus norvegicus GN=Gstm3 PE=1 SV=2

Query	Observed	Mr(expt)	Mr(calc)	ppm	Miss	Score	Expect	Rank	Unique	Peptide

Query	Observed	Mr(expt)	Mr(calc)	ppm	Miss	Score	Expect	Rank	Unique	Peptide
81	378.68	755.35	755.35	1.13	0	31	0.0027	1		K.ISDYMK.S
628	478.76	955.50	955.50	2.05	0	40	0.00056	1		R.LYSEFLGK.R
678	488.74	975.47	975.49	-19.76	1	30	0.0074	1		K.RPWFAGDK.I
791	508.29	1014.57	1014.58	-7.77	0	71	5.8e-007	1		K.ITQSNAILR.Y
1610	634.31	1266.60	1266.61	-2.75	0	88	2.3e-008	1		M.PMTLGYWDIR.G
1887	458.53	1372.56	1372.57	-3.36	0	40	0.00017	1		K.HNLCGETEEER.I 1886
2481	795.38	1588.76	1588.75	2.88	0	84	2.2e-008	1		R.LLLEYTDSSYEEK.R
2980	633.67	1897.99	1897.99	-1.57	0	110	5.3e-011	1		K.LGLDFPNLPYLIDGSHK.I

12. Q68FU3 **Mass:** 27898 **Score:** 479 **Matches:** 9(9) **Sequences:** 8(8) **emPAI:** 2.26
Electron transfer flavoprotein subunit beta OS=Rattus norvegicus GN=Etfb PE=2 SV=3

Query	Observed	Mr(expt)	Mr(calc)	ppm	Miss	Score	Expect	Rank	Unique	Peptide
345	431.24	860.46	860.46	-2.21	0	53	4.9e-005	1	U	K.SGVVTDGVK.H
479	452.78	903.54	903.54	-4.54	0	53	1.7e-005	1	U	K.VDLLFLGK.Q
974	538.28	1074.55	1074.56	-0.73	0	96	1.8e-009	1	U	K.AGDLGVDLTSK.V
1287	581.34	1160.67	1160.68	-7.50	1	49	3.6e-005	1	U	K.EKVDLLFLGK.Q
1679	648.40	1294.79	1294.80	-2.14	1	60	9.5e-007	1	U	R.LKLPAVVTADLR.L
1792	447.24	1338.71	1338.71	-1.44	0	59	7.4e-006	1	U	K.VSVISVEEPPQR.L
1793	670.36	1338.71	1338.71	-0.86	0	(43)	0.00028	1	U	K.VSVISVEEPPQR.L
2445	524.97	1571.87	1571.88	-2.21	1	85	1.1e-008	1	U	R.LAGVKVETTEDLVAK.L
3265	1078.58	2155.14	2155.14	-0.37	0	23	0.031	1	U	R.GIHVEVPGAEAENLGPLQVAR.V

13. Q63279 **Mass:** 44609 **Score:** 456 **Matches:** 8(8) **Sequences:** 8(8) **emPAI:** 1.10
Keratin, type I cytoskeletal 19 OS=Rattus norvegicus GN=Krt19 PE=1 SV=2

Query	Observed	Mr(expt)	Mr(calc)	ppm	Miss	Score	Expect	Rank	Unique	Peptide
162	404.20	806.39	806.39	-0.81	0	43	0.00034	1		R.LAADDFR.T
830	515.30	1028.59	1028.59	-0.41	0	52	6.2e-005	1		R.VLDELTLAR.T
860	521.30	1040.59	1040.60	-4.84	0	60	4.3e-006	1		K.IVLQIDNAR.L
887	523.29	1044.56	1044.58	-20.19	0	54	4e-005	1	U	K.ILGATIENSK.I
950	355.54	1063.60	1063.60	-0.78	1	60	4.8e-006	1		R.LASYLDKVR.A
1491	613.31	1224.61	1224.61	-2.28	0	71	4.9e-007	1	U	R.IVSSSSGGYVGGR.G
1689	650.84	1299.66	1299.67	-4.23	0	81	5.1e-008	1	U	R.ALEQANGELEVK.I
1897	459.57	1375.69	1375.69	1.05	1	35	0.0023	1	U	R.MSVEADINGLRR.V

14. Q10758 **Mass:** 53985 **Score:** 439 **Matches:** 11(11) **Sequences:** 10(10) **emPAI:** 1.33
Keratin, type II cytoskeletal 8 OS=Rattus norvegicus GN=Krt8 PE=1 SV=3

Query	Observed	Mr(expt)	Mr(calc)	ppm	Miss	Score	Expect	Rank	Unique	Peptide
209	414.22	826.42	826.42	-2.19	0	32	0.004	1		K.FASFIDK.V
367	435.72	869.42	869.42	-0.18	0	52	3e-005	1	U	R.ISSSSFSR.V
391	440.22	878.42	878.42	-4.25	0	31	0.007	1	U	R.SFTSGPGAR.I
500	455.71	909.41	909.41	3.77	0	34	0.0019	1		K.YEDEINK.R
908	351.85	1052.52	1052.53	-3.72	0	49	7.7e-005	1	U	R.QIHEEEIR.E
1086	547.27	1092.52	1092.52	-0.95	0	60	8.1e-006	1	U	R.AQYEEIANR.S
1584	627.33	1252.65	1252.68	-19.25	0	54	2.5e-005	1	U	K.LEVDPNIQAVR.T
1815	674.84	1347.67	1347.67	-3.08	0	51	5.6e-005	1	U	R.SLDMDSIIAEVR.A
1867	682.84	1363.66	1363.67	-1.81	0	(34)	0.0027	1	U	R.SLDMDSIIAEVR.A
1877	684.84	1367.67	1367.66	7.27	0	44	0.00028	1	U	R.TEMENEFVLIK.K
2949	940.40	1878.78	1878.79	-5.62	0	32	0.00069	1	U	R.SNMDNMFESYINNLR.R

15. P62703 **Mass:** 29807 **Score:** 430 **Matches:** 10(10) **Sequences:** 10(10) **emPAI:** 2.98
40S ribosomal protein S4, X isoform OS=Rattus norvegicus GN=Rps4x PE=2 SV=2

Query	Observed	Mr(expt)	Mr(calc)	ppm	Miss	Score	Expect	Rank	Unique	Peptide
74	376.71	751.41	751.41	-0.96	0	33	0.0018	1	U	R.LIYDTK.G
106	386.74	771.46	771.46	-0.82	0	36	0.0025	1	U	R.IGVITNR.E
223	416.23	830.45	830.45	-0.82	0	55	3.6e-005	1	U	R.LTIAEER.D
310	423.24	844.47	844.47	-1.64	0	28	0.02	1	U	R.YPDPLIK.V
343	430.75	859.49	859.49	-2.35	0	68	1.3e-006	1	U	K.LTGVFAPR.P
712	495.80	989.59	989.59	-3.34	0	56	9.2e-006	1	U	R.LSNIFVIGK.G
726	498.26	994.50	994.50	-0.63	0	26	0.018	1	U	K.YALTGDEVK.K
1310	389.89	1166.65	1166.66	-1.96	1	55	1.2e-005	1	U	K.GNKPWISLPR.G
1629	637.37	1272.73	1272.73	3.28	0	47	7.3e-005	1	U	R.ECLPLIIFLR.N
2840	899.92	1797.82	1797.81	3.49	0	26	0.01	1	U	K.FDTGNLCMVTGGANLGR.I

16. P21913 **Mass:** 32607 **Score:** 426 **Matches:** 8(8) **Sequences:** 6(6) **emPAI:** 1.75
Succinate dehydrogenase [ubiquinone] iron-sulfur subunit, mitochondrial OS=Rattus norvegicus GN=Sdhb PE=2 SV=2

Query	Observed	Mr(expt)	Mr(calc)	ppm	Miss	Score	Expect	Rank	Unique	Peptide
101	385.71	769.41	769.41	-2.85	0	38	0.00072	1	U	K.TFAIYR.W
1230	569.80	1137.58	1137.58	-4.01	0	59	8.4e-006	1	U	K.LQDPFSLYR.C
1464	608.82	1215.63	1215.64	-3.16	0	(47)	0.00018	1	U	K.CGPMVLDALIK.I
1507	616.82	1231.63	1231.63	-2.92	0	107	1.8e-010	1	U	K.CGPMVLDALIK.I
1911	691.37	1380.72	1380.72	-4.75	0	(21)	0.052	1	U	K.YLGPAVLMQAYR.W
1957	699.36	1396.71	1396.72	-5.42	0	80	8.2e-008	1	U	K.YLGPAVLMQAYR.W
2038	479.60	1435.77	1435.77	3.07	1	79	6e-008	1	U	K.IKNEIDSTLTFR.R
2621	836.92	1671.82	1671.81	1.53	0	62	4e-006	1	U	K.DLVPDLSNFYAQYK.S

17. P01836 **Mass:** 11896 **Score:** 423 **Matches:** 9(9) **Sequences:** 5(5) **emPAI:** 6.73
Ig kappa chain C region, A allele OS=Rattus norvegicus PE=1 SV=1

Query	Observed	Mr(expt)	Mr(calc)	ppm	Miss	Score	Expect	Rank	Unique	Peptide
1156	559.78	1117.55	1117.55	-0.70	1	43	0.00041	1	U	K.WKIDGSEQR.D
1901	689.82	1377.63	1377.63	0.35	0	101	3.3e-010	1	U	R.DGVLDSVTDQDSK.D 1902 1903
1962	467.23	1398.67	1398.67	0.73	0	76	1.2e-007	1	U	R.HNLYTCEVVHK.T 1961
2273	760.87	1519.72	1519.71	7.73	0	(62)	4e-006	1	U	K.DSTYSMSSTLSLTK.V
2344	768.86	1535.70	1535.70	0.84	0	89	5.2e-009	1	U	K.DSTYSMSSTLSLTK.V

Query	Observed	Mr(expt)	Mr(calc)	ppm	Miss	Score	Expect	Rank	Unique	Peptide
3562	966.11	2895.32	2895.32	0.73	1	114	1.2e-011	1	U	R.DGVLDSVTDQDSKDSTYSMSSTLSLTK.V

18. P62260 **Mass:** 29326 **Score:** 404 **Matches:** 7(7) **Sequences:** 6(6) **emPAI:** 1.68
14-3-3 protein epsilon OS=Rattus norvegicus GN=Ywhae PE=1 SV=1

Query	Observed	Mr(expt)	Mr(calc)	ppm	Miss	Score	Expect	Rank	Unique	Peptide
493	454.27	906.52	906.52	-0.94	0	44	0.00019	1		R.NLLSVAYK.N
719	497.25	992.48	992.48	-1.09	0	53	4.8e-005	1	U	R.QMVETELK.L
1394	595.33	1188.65	1188.65	-3.18	0	67	1.7e-006	1		K.DSTLIMQLLR.D
1407	597.80	1193.59	1193.59	-2.30	0	78	1.4e-007	1	U	K.EAAENSLVAYK.A
1442	603.33	1204.65	1204.65	-1.59	0	(66)	1.9e-006	1		K.DSTLIMQLLR.D
2127	732.36	1462.70	1462.70	3.48	0	114	2.4e-011	1	U	K.VAGMDVELTVEER.N
2163	738.87	1475.73	1475.74	-1.03	0	49	9.2e-005	1	U	K.LICCDILDVLDK.H

19. P41123 **Mass:** 24351 **Score:** 369 **Matches:** 7(7) **Sequences:** 7(7) **emPAI:** 2.27
60S ribosomal protein L13 OS=Rattus norvegicus GN=Rpl13 PE=1 SV=2

Query	Observed	Mr(expt)	Mr(calc)	ppm	Miss	Score	Expect	Rank	Unique	Peptide
85	379.75	757.48	757.49	-3.62	0	39	0.00054	1	U	K.LILFPR.K
607	475.75	949.49	949.49	-1.06	0	40	0.00082	1	U	R.GFSLEELR.V
631	479.27	956.52	956.53	-6.87	0	42	0.00044	1	U	R.TIGISVDPR.R
1290	388.24	1161.70	1161.70	-0.34	1	38	0.00022	1	U	R.PASGPIRPIVR.C
1506	616.81	1231.60	1231.62	-8.83	0	69	1.2e-006	1	U	K.STESLQANVQR.L
1510	617.30	1232.60	1232.59	1.12	0	58	1e-005	1	U	R.VDTWFNQPAR.K
1989	706.89	1411.77	1411.79	-7.67	0	82	2.6e-008	1	U	K.LATQLTGPVMPIR.N

20. P15999 **Mass:** 59831 **Score:** 367 **Matches:** 6(6) **Sequences:** 6(6) **emPAI:** 0.52
ATP synthase subunit alpha, mitochondrial OS=Rattus norvegicus GN=Atp5a1 PE=1 SV=2

Query	Observed	Mr(expt)	Mr(calc)	ppm	Miss	Score	Expect	Rank	Unique	Peptide
186	408.23	814.44	814.45	-13.58	0	32	0.005	1	U	R.ELIIGDR.Q
736	500.79	999.56	999.57	-8.14	0	49	9.2e-005	1	U	R.VLSIGDGIAR.V
819	513.80	1025.58	1025.59	-5.20	0	51	3.4e-005	1	U	K.AVDSLVPIGR.G
1322	586.31	1170.61	1170.62	-8.13	0	78	1.1e-007	1	U	R.VVDALGNAIDGK.G
1738	658.86	1315.71	1315.73	-19.03	0	80	7e-008	1	U	K.TSIAIDTIINQK.R
2524	805.94	1609.86	1609.87	-3.79	0	76	1.5e-007	1	U	R.TGAIVDVPVGDELLGR.V

21. Q6IMF3 **Mass:** 65190 **Score:** 355 **Matches:** 6(6) **Sequences:** 6(6) **emPAI:** 0.47
Keratin, type II cytoskeletal 1 OS=Rattus norvegicus GN=Krt1 PE=2 SV=1

Query	Observed	Mr(expt)	Mr(calc)	ppm	Miss	Score	Expect	Rank	Unique	Peptide
209	414.22	826.42	826.42	-2.19	0	32	0.004	1		K.FASFIDK.V
500	455.71	909.41	909.41	3.77	0	34	0.0019	1		K.YEDEINK.R
1276	579.26	1156.51	1156.51	1.93	0	47	5.6e-005	1	U	R.DYQELMNTK.L
1944	465.25	1392.73	1392.72	0.11	1	89	1.1e-008	1	U	R.TNAENEFVTIKK.D
2158	738.38	1474.74	1474.74	1.03	0	70	6.7e-007	1	U	K.WELLQQVDTSTR.T
2159	738.40	1474.78	1474.78	-1.48	0	85	1.9e-008	1		R.FLEQQNQVLQTK.W

22. P00406 **Mass:** 26096 **Score:** 352 **Matches:** 10(10) **Sequences:** 7(7) **emPAI:** 3.13
Cytochrome c oxidase subunit 2 OS=Rattus norvegicus GN=Mtco2 PE=2 SV=2

Query	Observed	Mr(expt)	Mr(calc)	ppm	Miss	Score	Expect	Rank	Unique	Peptide
35	365.20	728.38	728.38	-0.78	0	40	0.00058	1	U	K.TDAIPGR.L
339	429.74	857.46	857.46	0.94	0	49	0.0001	1	U	R.LLEVDNR.V
1113	552.29	1102.57	1102.57	-4.50	0	53	4e-005	1	U	R.LNQATVTSNR.P
1307	583.85	1165.69	1165.69	-2.09	0	(54)	5.8e-006	1	U	R.VVLPMELPIR.M 1306
1369	591.85	1181.68	1181.68	-3.84	0	60	3.4e-006	1	U	R.VVLPMELPIR.M
1603	632.27	1262.53	1262.53	4.78	0	26	0.0051	1	U	K.YFENWSASMI.-
2991	954.99	1907.97	1907.97	0.11	0	(21)	0.05	1	U	R.ILYMMDEINNPVLTVK.T
3012	962.99	1923.97	1923.97	0.25	0	94	2.1e-009	1	U	R.ILYMMDEINNPVLTVK.T
3310	738.06	2211.16	2211.16	-1.15	0	30	0.0034	1	U	R.MLISSEDVLHSWAIPSLGLK.T

23. P38983 **Mass:** 32917 **Score:** 343 **Matches:** 6(6) **Sequences:** 6(6) **emPAI:** 1.12
40S ribosomal protein SA OS=Rattus norvegicus GN=Rpsa PE=1 SV=3

Query	Observed	Mr(expt)	Mr(calc)	ppm	Miss	Score	Expect	Rank	Unique	Peptide
506	456.78	911.54	911.54	-3.13	0	51	2e-005	1	U	R.LLVVTDPR.A
1604	632.37	1262.73	1262.72	4.44	1	41	0.00021	1	U	R.KSDGIYIINLK.R
1712	653.83	1305.64	1305.64	1.49	0	35	0.0023	1	U	R.YVDIAIPCNNK.G
2663	849.93	1697.85	1697.85	-0.43	0	78	8.1e-008	1	U	R.FTPGTFTNQIQAAFR.E
2739	870.98	1739.94	1739.94	-0.13	0	117	8.3e-012	1	U	R.AIVAIENPADVSVISSR.N
3589	999.50	2995.47	2995.47	0.52	0	21	0.025	1	U	R.ADHQPLTEASYVNLPTIALCNTDSPLR.Y

24. Q6IFW6 **Mass:** 56699 **Score:** 316 **Matches:** 11(11) **Sequences:** 7(7) **emPAI:** 0.67
Keratin, type I cytoskeletal 10 OS=Rattus norvegicus GN=Krt10 PE=3 SV=1

Query	Observed	Mr(expt)	Mr(calc)	ppm	Miss	Score	Expect	Rank	Unique	Peptide
162	404.20	806.39	806.39	-0.81	0	43	0.00034	1		R.LAADDFR.L
313	424.23	846.44	846.44	-1.94	0	37	0.002	1	U	K.SEITELR.R
1124	553.77	1105.52	1105.52	-0.99	0	78	1.3e-007	1		K.VTMQNLNDR.L 1121 1122 1123 1125
1132	555.26	1108.50	1108.48	15.2	0	39	0.00055	1	U	K.DAEAWFNEK.S
1425	401.21	1200.61	1200.61	-0.67	0	43	0.00039	1	U	R.QSVEADINGLR.R
1512	617.83	1233.65	1233.67	-14.09	1	24	0.03	1	U	R.LKYENEVALR.Q
1838	453.24	1356.71	1356.71	-1.24	1	51	5.4e-005	1	U	R.QSVEADINGLRR.V

25. Q05962 **Mass:** 33196 **Score:** 264 **Matches:** 7(7) **Sequences:** 6(6) **emPAI:** 1.11
ADP/ATP translocase 1 OS=Rattus norvegicus GN=Slc25a4 PE=1 SV=3

Query	Observed	Mr(expt)	Mr(calc)	ppm	Miss	Score	Expect	Rank	Unique	Peptide
108	387.20	772.38	772.38	-3.64	0	30	0.0052	1		K.GMLPDPK.N

332	428.75	855.48	855.48	-1.23	0	54	2.7e-005	1		K.TAVAPIER.V
333	428.76	855.50	855.49	3.67	0	26	0.011	1		R.GNLANVIR.Y
1476	610.34	1218.66	1218.66	-2.43	0	25	0.022	2	U	K.DFLAGGIAAAVSK.T 1475
1864	455.28	1362.82	1362.83	-9.05	1	67	2.1e-007	1		R.VKLLLQVQHASK.Q
2076	723.87	1445.74	1445.73	0.64	0	65	2.5e-006	1		R.YFPTQALNFAFK.D

26. Q6P6Q2 **Mass:** 61959 **Score:** 242 **Matches:** 5(5) **Sequences:** 5(5) **emPAI:** 0.40
Keratin, type II cytoskeletal 5 OS=Rattus norvegicus GN=Krt5 PE=1 SV=1

Query	Observed	Mr(expt)	Mr(calc)	ppm	Miss	Score	Expect	Rank	Unique	Peptide
209	414.22	826.42	826.42	-2.19	0	32	0.004	1		K.FASFIDK.V
500	455.71	909.41	909.41	3.77	0	34	0.0019	1		K.YEDEINK.R
753	**503.24**	**1004.47**	**1004.46**	**11.6**	**0**	**44**	**0.00026**	**1**		**K.LLEGEECR.L**
1432	**602.32**	**1202.64**	**1202.63**	**4.55**	**0**	**31**	**0.0056**	**1**	**U**	**K.WTLLQEQGTK.T**
1694	**651.86**	**1301.71**	**1301.71**	**-1.52**	**0**	**101**	**4.8e-010**	**1**		**R.SLDLDSIIAEVK.A**

27. A7VJC2 **Mass:** 37512 **Score:** 239 **Matches:** 5(5) **Sequences:** 5(5) **emPAI:** 0.73
Heterogeneous nuclear ribonucleoproteins A2/B1 OS=Rattus norvegicus GN=Hnrnpa2b1 PE=1 SV=1

Query	Observed	Mr(expt)	Mr(calc)	ppm	Miss	Score	Expect	Rank	Unique	Peptide
769	**505.25**	**1008.48**	**1008.47**	**6.50**	**0**	**50**	**0.00017**	**1**	**U**	**K.LTDCVVMR.D**
779	**507.23**	**1012.44**	**1012.44**	**4.53**	**0**	**22**	**0.017**	**1**	**U**	**R.GGNFGFGDSR.G**
1387	**594.83**	**1187.64**	**1187.64**	**3.95**	**0**	**70**	**8.1e-007**	**1**	**U**	**K.IDTIEIITDR.Q**
1481	**611.28**	**1220.54**	**1220.55**	**-3.30**	**0**	**36**	**0.00099**	**1**	**U**	**R.QEMQEVQSSR.S**
1984	**353.43**	**1409.68**	**1409.68**	**-1.60**	**0**	**61**	**5.7e-006**	**1**	**U**	**K.YHTINGHNAEVR.K**

28. P10719 **Mass:** 56318 **Score:** 237 **Matches:** 4(4) **Sequences:** 4(4) **emPAI:** 0.34
ATP synthase subunit beta, mitochondrial OS=Rattus norvegicus GN=Atp5b PE=1 SV=2

Query	Observed	Mr(expt)	Mr(calc)	ppm	Miss	Score	Expect	Rank	Unique	Peptide
115	**390.21**	**778.41**	**778.42**	**-13.10**	**0**	**35**	**0.0025**	**1**	**U**	**K.ILQDYK.S**
676	**488.28**	**974.55**	**974.55**	**-6.86**	**0**	**57**	**1.1e-005**	**1**	**U**	**K.IGLFGGAGVGK.T**
2036	**718.38**	**1434.75**	**1434.75**	**0.53**	**0**	**50**	**6.8e-005**	**1**	**U**	**R.FTQAGSEVSALLGR.I**
2045	**720.39**	**1438.77**	**1438.78**	**-8.00**	**0**	**94**	**2e-009**	**1**	**U**	**R.VALTGLTVAEYFR.D**

29. Q6IFV3 **Mass:** 49011 **Score:** 232 **Matches:** 8(8) **Sequences:** 4(4) **emPAI:** 0.40
Keratin, type I cytoskeletal 15 OS=Rattus norvegicus GN=Krt15 PE=1 SV=1

Query	Observed	Mr(expt)	Mr(calc)	ppm	Miss	Score	Expect	Rank	Unique	Peptide
162	404.20	806.39	806.39	-0.81	0	43	0.00034	1		R.LAADDFR.L
830	515.30	1028.59	1028.59	-0.41	0	52	6.2e-005	1		R.VLDELTLAR.T
950	355.54	1063.60	1063.60	-0.78	1	60	4.8e-006	1		R.LASYLDKVR.A
1124	553.77	1105.52	1105.52	-0.99	0	78	1.3e-007	1		K.VTMQNLNDR.L 1121 1122 1123 1125

30. P61983 **Mass:** 28456 **Score:** 219 **Matches:** 6(5) **Sequences:** 5(4) **emPAI:** 1.06
14-3-3 protein gamma OS=Rattus norvegicus GN=Ywhag PE=1 SV=2

Query	Observed	Mr(expt)	Mr(calc)	ppm	Miss	Score	Expect	Rank	Unique	Peptide
493	454.27	906.52	906.52	-0.94	0	44	0.00019	1		R.NLLSVAYK.N
992	**540.78**	**1079.55**	**1079.55**	**-1.37**	**0**	**49**	**9.4e-005**	**1**	**U**	**R.YLAEVATGEK.R**
1394	595.33	1188.65	1188.65	-3.18	0	67	1.7e-006	1		K.DSTLIMQLLR.D
1442	603.33	1204.65	1204.65	-1.59	0	(66)	1.9e-006	1		K.DSTLIMQLLR.D
1523	**412.89**	**1235.64**	**1235.65**	**-10.86**	**1**	**21**	**0.062**	**1**	**U**	**R.YLAEVATGEKR.A**
2576	**822.39**	**1642.77**	**1642.78**	**-3.36**	**0**	**39**	**0.00058**	**1**	**U**	**K.NVTELNEPLSNEER.N**

31. P68255 **Mass:** 28046 **Score:** 217 **Matches:** 5(5) **Sequences:** 4(4) **emPAI:** 1.08
14-3-3 protein theta OS=Rattus norvegicus GN=Ywhaq PE=1 SV=1

Query	Observed	Mr(expt)	Mr(calc)	ppm	Miss	Score	Expect	Rank	Unique	Peptide
493	454.27	906.52	906.52	-0.94	0	44	0.00019	1		R.NLLSVAYK.N
1394	595.33	1188.65	1188.65	-3.18	0	67	1.7e-006	1		K.DSTLIMQLLR.D
1442	603.33	1204.65	1204.65	-1.59	0	(66)	1.9e-006	1		K.DSTLIMQLLR.D
1937	**696.37**	**1390.73**	**1390.74**	**-7.27**	**0**	**68**	**2.6e-006**	**1**	**U**	**R.SICTTVLELLDK.Y**
1955	**466.22**	**1395.64**	**1395.65**	**-1.46**	**1**	**39**	**0.00055**	**1**	**U**	**R.YLAEVACGDDRK.Q**

32. P35435 **Mass:** 30229 **Score:** 214 **Matches:** 5(5) **Sequences:** 5(5) **emPAI:** 0.98
ATP synthase subunit gamma, mitochondrial OS=Rattus norvegicus GN=Atp5c1 PE=1 SV=2

Query	Observed	Mr(expt)	Mr(calc)	ppm	Miss	Score	Expect	Rank	Unique	Peptide
351	**432.75**	**863.49**	**863.49**	**0.11**	**0**	**38**	**0.00078**	**1**	**U**	**K.LTLTFNR.T**
987	**539.77**	**1077.52**	**1077.51**	**8.70**	**0**	**61**	**5.9e-006**	**1**	**U**	**K.NDMAALTAAGK.E**
1093	**366.21**	**1095.62**	**1095.60**	**10.6**	**0**	**21**	**0.033**	**1**	**U**	**K.HLIIGVSSDR.G**
1717	**654.83**	**1307.65**	**1307.65**	**-1.99**	**0**	**37**	**0.0014**	**1**	**U**	**R.THSDQFLVSFK.D**
1731	**657.86**	**1313.70**	**1313.71**	**-6.78**	**0**	**56**	**1.7e-005**	**1**	**U**	**K.ELIEIISGAAALD.-**

33. P25113 **Mass:** 28928 **Score:** 210 **Matches:** 4(4) **Sequences:** 4(4) **emPAI:** 0.77
Phosphoglycerate mutase 1 OS=Rattus norvegicus GN=Pgam1 PE=1 SV=4

Query	Observed	Mr(expt)	Mr(calc)	ppm	Miss	Score	Expect	Rank	Unique	Peptide
921	**530.29**	**1058.56**	**1058.55**	**8.62**	**0**	**43**	**0.00051**	**1**	**U**	**R.HYGGLTGLNK.A**
1261	**384.23**	**1149.66**	**1149.66**	**-1.28**	**0**	**66**	**6.7e-007**	**1**	**U**	**R.VLIAAHGNSLR.G**
2643	**842.46**	**1682.90**	**1682.90**	**-0.75**	**0**	**71**	**3.5e-007**	**1**	**U**	**R.ALPFWNEEIVPQIK.E**
2934	**934.94**	**1867.86**	**1867.85**	**2.92**	**0**	**30**	**0.0048**	**1**	**U**	**R.YADLTEDQLPSCESLK.D**

34. P19234 **Mass:** 27703 **Score:** 209 **Matches:** 5(5) **Sequences:** 4(4) **emPAI:** 0.81
NADH dehydrogenase [ubiquinone] flavoprotein 2, mitochondrial OS=Rattus norvegicus GN=Ndufv2 PE=1 SV=2

Query	Observed	Mr(expt)	Mr(calc)	ppm	Miss	Score	Expect	Rank	Unique	Peptide
3	**350.72**	**699.43**	**699.43**	**1.61**	**0**	**23**	**0.04**	**1**	**U**	**R.IEAIVR.N**

1548	622.82	1243.63	1243.63	-2.46	0	57	1.4e-005	1	U	K.DIEEIIDELR.A
1635	638.82	1275.63	1275.63	-1.64	0	67	1.5e-006	1	U	R.DSDSILETLQR.K
1784	668.90	1335.78	1335.79	-2.82	0	(60)	2.5e-006	1	U	R.AAAVLPVLDLAQR.Q
1785	446.27	1335.79	1335.79	-1.30	0	62	1.6e-006	1	U	R.AAAVLPVLDLAQR.Q

35. P56571 **Mass:** 28497 **Score:** 209 **Matches:** 4(4) **Sequences:** 4(4) **emPAI:** 0.78
ES1 protein homolog, mitochondrial OS=Rattus norvegicus PE=1 SV=2

Query	Observed	Mr(expt)	Mr(calc)	ppm	Miss	Score	Expect	Rank	Unique	Peptide
340	430.24	858.46	858.46	1.20	0	41	0.00096	1	U	R.NVLAESAR.I
2402	518.92	1553.73	1553.73	-1.82	0	24	0.021	1	U	K.GVEVTVGHEQEEGGK.W
2525	805.94	1609.87	1609.90	-19.21	1	100	5.2e-010	1	U	K.KPIGLCCIAPVLAAK.V
3436	819.10	2454.27	2454.27	0.36	0	45	0.00012	1	U	K.ITNLAQLSAANHDAAIFPGGFGAAK.N

36. P29410 **Mass:** 26648 **Score:** 207 **Matches:** 5(5) **Sequences:** 5(5) **emPAI:** 1.16
Adenylate kinase 2, mitochondrial OS=Rattus norvegicus GN=Ak2 PE=2 SV=2

Query	Observed	Mr(expt)	Mr(calc)	ppm	Miss	Score	Expect	Rank	Unique	Peptide
692	490.29	978.57	978.59	-15.25	0	30	0.0029	1	U	R.AVLLGPPGAGK.G
951	533.26	1064.51	1064.52	-4.84	0	44	0.00029	1	U	R.AMVASGSELGK.K
1216	568.30	1134.58	1134.58	-2.94	0	43	0.00045	1	U	K.NGFLLDGFPR.T
1556	416.53	1246.56	1246.56	-1.14	0	38	0.00055	1	U	R.SYHEEFNPPK.E
2270	760.40	1518.78	1518.79	-5.84	0	52	4e-005	1	U	K.LVSDEMVVELIEK.N

37. P84100 **Mass:** 23565 **Score:** 203 **Matches:** 6(5) **Sequences:** 6(5) **emPAI:** 1.39
60S ribosomal protein L19 OS=Rattus norvegicus GN=Rpl19 PE=1 SV=1

Query	Observed	Mr(expt)	Mr(calc)	ppm	Miss	Score	Expect	Rank	Unique	Peptide
62	373.23	744.44	744.45	-13.36	0	51	8.3e-005	1	U	R.LASSVLR.C
702	493.77	985.52	985.52	-1.26	0	63	3.9e-006	1	U	K.LLADQAEAR.R
846	518.78	1035.54	1035.55	-15.76	0	23	0.043	1	U	R.ILMEHIHK.L
1147	372.21	1113.61	1113.61	-6.39	1	20	0.063	1	U	K.KLLADQAEAR.R
1445	403.20	1206.58	1206.59	-3.52	0	24	0.03	1	U	R.HMYHSLYLK.V
3042	971.97	1941.93	1941.92	5.41	0	22	0.028	1	U	K.VWLDPNETNEIANANSR.Q

38. Q4FZU2 **Mass:** 59555 **Score:** 198 **Matches:** 3(3) **Sequences:** 3(3) **emPAI:** 0.23
Keratin, type II cytoskeletal 6A OS=Rattus norvegicus GN=Krt6a PE=1 SV=1

Query	Observed	Mr(expt)	Mr(calc)	ppm	Miss	Score	Expect	Rank	Unique	Peptide
209	414.22	826.42	826.42	-2.19	0	32	0.004	1		K.FASFIDK.V
1354	590.30	1178.59	1178.59	-0.90	0	65	2.4e-006	1	U	K.YEELQITAGR.H
1694	651.86	1301.71	1301.71	-1.52	0	101	4.8e-010	1		R.SLDLDSIIAEVK.A

39. P20767 **Mass:** 11482 **Score:** 192 **Matches:** 4(4) **Sequences:** 4(4) **emPAI:** 3.12
Ig lambda-2 chain C region OS=Rattus norvegicus PE=4 SV=1

Query	Observed	Mr(expt)	Mr(calc)	ppm	Miss	Score	Expect	Rank	Unique	Peptide
627	478.76	955.50	955.51	-9.89	0	35	0.0017	1	U	K.YIASSFLR.L
2572	820.92	1639.82	1639.82	1.54	0	87	1.2e-008	1	U	K.ANGAPISQGVDTANPTK.Q
2898	617.62	1849.83	1849.83	0.18	0	26	0.0068	1	U	R.NSFTCQVTHEGNTVEK.S
3177	1023.52	2045.03	2045.03	-0.34	0	44	0.00018	1	U	K.STPTLTVFPPSTEELQGNK.A

40. Q99P74 **Mass:** 24832 **Score:** 191 **Matches:** 4(4) **Sequences:** 4(4) **emPAI:** 0.94
Ras-related protein Rab-27B OS=Rattus norvegicus GN=Rab27b PE=2 SV=3

Query	Observed	Mr(expt)	Mr(calc)	ppm	Miss	Score	Expect	Rank	Unique	Peptide
828	515.30	1028.58	1028.59	-2.94	0	62	5.8e-006	1	U	K.LLALGDSGVGK.T
1640	639.36	1276.70	1276.69	5.06	0	62	3.4e-006	1	U	K.SVETLLDLIMK.R
2358	513.92	1538.73	1538.73	-1.20	1	37	0.0014	1	U	K.RVVYDTQGADGSSGK.A
3083	987.97	1973.93	1973.94	-4.19	0	31	0.004	1	U	K.YGIPYFETSAATGQNVEK.S

41. P35213 **Mass:** 28151 **Score:** 187 **Matches:** 4(4) **Sequences:** 3(3) **emPAI:** 0.79
14-3-3 protein beta/alpha OS=Rattus norvegicus GN=Ywhab PE=1 SV=3

Query	Observed	Mr(expt)	Mr(calc)	ppm	Miss	Score	Expect	Rank	Unique	Peptide
493	454.27	906.52	906.52	-0.94	0	44	0.00019	1		R.NLLSVAYK.N
1394	595.33	1188.65	1188.65	-3.18	0	67	1.7e-006	1		K.DSTLIMQLLR.D
1442	603.33	1204.65	1204.65	-1.59	0	(66)	1.9e-006	1		K.DSTLIMQLLR.D
2494	533.58	1597.73	1597.73	-2.48	0	77	1e-007	1	U	K.AVTEQGHELSNEER.N

42. P32551 **Mass:** 48423 **Score:** 185 **Matches:** 4(4) **Sequences:** 4(4) **emPAI:** 0.41
Cytochrome b-c1 complex subunit 2, mitochondrial OS=Rattus norvegicus GN=Uqcrc2 PE=1 SV=2

Query	Observed	Mr(expt)	Mr(calc)	ppm	Miss	Score	Expect	Rank	Unique	Peptide
59	372.23	742.44	742.43	3.26	0	42	0.0011	1	U	R.GGLGLAGAK.A
67	374.22	746.42	746.43	-9.22	0	53	4.6e-005	1	U	K.LSVTATR.E
229	417.75	833.49	833.49	0.39	0	58	9.2e-006	1	U	R.LASTLTTK.G
1470	609.84	1217.66	1217.64	14.9	0	33	0.0045	1	U	K.EVAEQFLNIR.G

43. P49242 **Mass:** 30154 **Score:** 177 **Matches:** 5(5) **Sequences:** 5(5) **emPAI:** 0.98
40S ribosomal protein S3a OS=Rattus norvegicus GN=Rps3a PE=1 SV=2

Query	Observed	Mr(expt)	Mr(calc)	ppm	Miss	Score	Expect	Rank	Unique	Peptide
555	468.24	934.47	934.47	2.69	0	35	0.0027	1	U	K.APAMFNIR.N
665	486.25	970.49	970.49	-2.84	0	34	0.0027	1	U	R.LFCVGFTK.K
744	501.77	1001.53	1001.54	-13.27	0	42	0.00077	1	U	K.LITEDVQGK.N
1741	439.88	1316.61	1316.60	3.73	0	34	0.0016	1	U	K.LMELHGEGGSSGK.T
2487	532.24	1593.71	1593.70	3.12	0	32	0.0021	1	U	K.NCLTNFHGMDLTR.D

44. Q5BJY9 **Mass:** 47732 **Score:** 170 **Matches:** 4(4) **Sequences:** 4(4) **emPAI:** 0.41
Keratin, type I cytoskeletal 18 OS=Rattus norvegicus GN=Krt18 PE=1 SV=3

Query	Observed	Mr(expt)	Mr(calc)	ppm	Miss	Score	Expect	Rank	Unique	Peptide
43	367.70	733.38	733.38	4.81	0	32	0.0061	1	U	K.IMADIR.A
162	404.20	806.39	806.39	-0.81	0	43	0.00034	1		R.LAADDFR.V
860	521.30	1040.59	1040.60	-4.84	0	60	4.3e-006	1		R.IVLQIDNAR.L
1587	419.22	1254.63	1254.63	0.88	1	35	0.0021	1	U	R.VKYETELAMR.Q

45. P23965 **Mass:** 32348 **Score:** 170 **Matches:** 4(4) **Sequences:** 4(4) **emPAI:** 0.67
Enoyl-CoA delta isomerase 1, mitochondrial OS=Rattus norvegicus GN=Eci1 PE=1 SV=1

Query	Observed	Mr(expt)	Mr(calc)	ppm	Miss	Score	Expect	Rank	Unique	Peptide
309	423.24	844.46	844.47	-4.86	0	36	0.0035	1	U	K.ATADNLIK.Q
708	494.78	987.54	987.54	-0.91	0	23	0.049	1	U	K.SLHVYLEK.L
1385	396.86	1187.57	1187.57	3.93	0	43	0.00034	1	U	K.DNYVNTIGHR.A
2759	583.97	1748.90	1748.89	0.86	0	68	7.5e-007	1	U	K.VGLVDEVVPEDQVHSK.A

46. P04904 **Mass:** 25360 **Score:** 166 **Matches:** 4(3) **Sequences:** 4(3) **emPAI:** 0.63
Glutathione S-transferase alpha-3 OS=Rattus norvegicus GN=Gsta3 PE=1 SV=3

Query	Observed	Mr(expt)	Mr(calc)	ppm	Miss	Score	Expect	Rank	Unique	Peptide
764	503.79	1005.57	1005.59	-14.62	0	54	1.8e-005	1	U	R.AILNYIATK.Y
926	354.20	1059.58	1059.58	-3.91	1	21	0.068	1	U	K.KFLQPGSQR.K
1550	623.30	1244.59	1244.59	0.09	0	52	4e-005	1	U	K.SHGQDYLVGNR.L
1921	462.58	1384.71	1384.73	-8.83	1	40	0.00076	1	U	M.PGKPVLHYFDGR.G

47. P01946 **Mass:** 15490 **Score:** 164 **Matches:** 4(4) **Sequences:** 4(4) **emPAI:** 1.86
Hemoglobin subunit alpha-1/2 OS=Rattus norvegicus GN=Hba1 PE=1 SV=3

Query	Observed	Mr(expt)	Mr(calc)	ppm	Miss	Score	Expect	Rank	Unique	Peptide
192	409.72	817.43	817.43	-0.27	0	34	0.004	1	U	R.VDPVNFK.F
1062	544.31	1086.61	1086.62	-5.27	1	21	0.061	1	U	K.LRVDPVNFK.F
1575	626.86	1251.70	1251.71	-6.05	0	73	1.8e-007	1	U	K.FLASVSTVLTSK.Y
2443	524.92	1571.73	1571.73	0.12	0	35	0.0014	1	U	K.IGGHGGEYGEEALQR.M

48. Q6IG03 **Mass:** 60977 **Score:** 162 **Matches:** 3(3) **Sequences:** 3(3) **emPAI:** 0.23
Keratin, type II cytoskeletal 73 OS=Rattus norvegicus GN=Krt73 PE=1 SV=1

Query	Observed	Mr(expt)	Mr(calc)	ppm	Miss	Score	Expect	Rank	Unique	Peptide
209	414.22	826.42	826.42	-2.19	0	32	0.004	1		K.FASFIDK.V
753	503.24	1004.47	1004.46	11.6	0	44	0.00026	1		K.LLEGEECR.M
2159	738.40	1474.78	1474.78	-1.48	0	85	1.9e-008	1		R.FLEQQNQVLQTK.W

49. P04692 **Mass:** 32718 **Score:** 157 **Matches:** 4(4) **Sequences:** 4(4) **emPAI:** 0.66
Tropomyosin alpha-1 chain OS=Rattus norvegicus GN=Tpm1 PE=1 SV=3

Query	Observed	Mr(expt)	Mr(calc)	ppm	Miss	Score	Expect	Rank	Unique	Peptide
60	372.73	743.45	743.45	0.82	0	44	0.00032	1		R.LATALQK.L
1203	566.31	1130.60	1130.60	1.22	0	26	0.021	1	U	K.MEIQEIQLK.E
1382	593.83	1185.65	1185.66	-7.56	0	55	2.2e-005	1	U	K.LVIIESDLER.A
2714	576.63	1726.88	1726.88	-2.04	1	32	0.0029	1		R.IQLVEEELDRAQER.L

50. P62907 **Mass:** 24987 **Score:** 154 **Matches:** 5(5) **Sequences:** 4(4) **emPAI:** 1.28
60S ribosomal protein L10a OS=Rattus norvegicus GN=Rpl10a PE=1 SV=2

Query	Observed	Mr(expt)	Mr(calc)	ppm	Miss	Score	Expect	Rank	Unique	Peptide
179	406.25	810.49	810.50	-5.27	0	31	0.0011	1	U	R.ILGPGLNK.A
655	483.75	965.49	965.48	7.78	0	39	0.001	1	U	R.DTLYEAVR.E
2461	395.72	1578.84	1578.84	-3.87	1	41	0.00048	1	U	K.AVDIPHMDIEALKK.L
2489	399.72	1594.84	1594.84	2.89	1	(36)	0.0011	1	U	K.AVDIPHMDIEALKK.L
2528	539.61	1615.81	1615.80	1.44	0	44	0.00024	1	U	K.FPSLLTHNENMVAK.V

51. Q9Z270 **Mass:** 28051 **Score:** 153 **Matches:** 4(4) **Sequences:** 4(4) **emPAI:** 0.80
Vesicle-associated membrane protein-associated protein A OS=Rattus norvegicus GN=Vapa PE=1 SV=3

Query	Observed	Mr(expt)	Mr(calc)	ppm	Miss	Score	Expect	Rank	Unique	Peptide
147	400.23	798.45	798.46	-7.87	0	24	0.021	1	U	K.AVPLNASK.Q
1641	426.87	1277.58	1277.58	-0.56	1	31	0.004	1	U	K.EAKPDELMDSK.L
2509	535.29	1602.86	1602.86	0.29	0	46	0.00011	1	U	K.HEQILVLDPPSDLK.F
2578	549.28	1644.83	1644.82	4.90	1	52	5.1e-005	1	U	K.VAHSDKPGSTSAVSFR.D

52. P29411 **Mass:** 25479 **Score:** 147 **Matches:** 4(3) **Sequences:** 4(3) **emPAI:** 0.62
GTP:AMP phosphotransferase AK3, mitochondrial OS=Rattus norvegicus GN=Ak3 PE=2 SV=2

Query	Observed	Mr(expt)	Mr(calc)	ppm	Miss	Score	Expect	Rank	Unique	Peptide
730	499.27	996.53	996.54	-0.99	0	33	0.003	1	U	K.HLSSGDLLR.Q
748	502.27	1002.53	1002.52	15.3	0	20	0.095	1	U	R.AVIMGAPGSGK.G
1144	557.30	1112.58	1112.58	-5.25	0	53	2.6e-005	1	U	R.TLPQAEALDR.V
2680	853.93	1705.84	1705.82	7.98	0	41	0.00055	1	U	K.NLTQCSWLLDGFPR.T

53. P34058 **Mass:** 83571 **Score:** 145 **Matches:** 3(3) **Sequences:** 3(3) **emPAI:** 0.16
Heat shock protein HSP 90-beta OS=Rattus norvegicus GN=Hsp90ab1 PE=1 SV=4

Query	Observed	Mr(expt)	Mr(calc)	ppm	Miss	Score	Expect	Rank	Unique	Peptide
855	520.25	1038.49	1038.49	5.09	0	54	3e-005	1		R.YESLTDPSK.L
1544	621.86	1241.70	1241.70	-1.25	0	63	2.2e-006	1		K.ADLINNLGTIAK.S
1870	683.36	1364.70	1364.72	-15.57	0	28	0.014	1	U	R.TLTLVDTGIGMTK.A

54. P82995 **Mass:** 85161 **Score:** 144 **Matches:** 3(3) **Sequences:** 3(3) **emPAI:** 0.16

Heat shock protein HSP 90-alpha OS=Rattus norvegicus GN=Hsp90aa1 PE=1 SV=3

Query	Observed	Mr(expt)	Mr(calc)	ppm	Miss	Score	Expect	Rank	Unique	Peptide
855	520.25	1038.49	1038.49	5.09	0	54	3e-005	1		R.YESLTDPSK.L
1544	621.86	1241.70	1241.70	-1.25	0	63	2.2e-006	1		K.ADLINNLGTIAK.S
1870	683.36	1364.70	1364.72	-15.57	0	28	0.014	1	U	R.TLTIVDTGIGMTK.A

55. P47727 **Mass:** 30844 **Score:** 142 **Matches:** 3(3) **Sequences:** 3(3) **emPAI:** 0.49

Carbonyl reductase [NADPH] 1 OS=Rattus norvegicus GN=Cbr1 PE=1 SV=2

Query	Observed	Mr(expt)	Mr(calc)	ppm	Miss	Score	Expect	Rank	Unique	Peptide
226	**416.75**	**831.48**	**831.50**	**-15.33**	**0**	**36**	**0.0016**	**1**	**U**	**K.GIGFAIVR.D**
2112	**729.87**	**1457.72**	**1457.73**	**-4.95**	**0**	**62**	**4.3e-006**	**1**	**U**	**K.ILLNACCPGWVR.T**
2468	**528.27**	**1581.79**	**1581.79**	**-1.45**	**0**	**44**	**0.00024**	**1**	**U**	**R.FHQLDIDNPQSIR.A**

56. P63039 **Mass:** 61088 **Score:** 135 **Matches:** 2(2) **Sequences:** 2(2) **emPAI:** 0.15

60 kDa heat shock protein, mitochondrial OS=Rattus norvegicus GN=Hspd1 PE=1 SV=1

Query	Observed	Mr(expt)	Mr(calc)	ppm	Miss	Score	Expect	Rank	Unique	Peptide
327	**428.24**	**854.46**	**854.46**	**-0.48**	**0**	**45**	**0.00012**	**1**	**U**	**K.GANPVEIR.R**
3486	**854.09**	**2559.24**	**2559.24**	**0.76**	**0**	**89**	**5e-009**	**1**	**U**	**K.LVQDVANNTNEEAGDGTTTATVLAR.S**

57. P68511 **Mass:** 28365 **Score:** 132 **Matches:** 4(4) **Sequences:** 3(3) **emPAI:** 0.79

14-3-3 protein eta OS=Rattus norvegicus GN=Ywhah PE=1 SV=2

Query	Observed	Mr(expt)	Mr(calc)	ppm	Miss	Score	Expect	Rank	Unique	Peptide
199	**412.22**	**822.43**	**822.41**	**23.3**	**0**	**22**	**0.046**	**1**	**U**	**K.EAFEISK.E**
493	454.27	906.52	906.52	-0.94	0	44	0.00019	1		R.NLLSVAYK.N
1394	595.33	1188.65	1188.65	-3.18	0	67	1.7e-006	1		K.DSTLIMQLLR.D
1442	603.33	1204.65	1204.65	-1.59	0	(66)	1.9e-006	1		K.DSTLIMQLLR.D

58. P12001 **Mass:** 21702 **Score:** 120 **Matches:** 2(1) **Sequences:** 2(1) **emPAI:** 0.21

60S ribosomal protein L18 OS=Rattus norvegicus GN=Rpl18 PE=2 SV=2

Query	Observed	Mr(expt)	Mr(calc)	ppm	Miss	Score	Expect	Rank	Unique	Peptide
1809	**673.36**	**1344.71**	**1344.72**	**-10.40**	**0**	**20**	**0.071**	**1**	**U**	**K.TAVVVGTITDDVR.I**
2156	**737.91**	**1473.81**	**1473.81**	**-0.36**	**0**	**100**	**4.4e-010**	**1**	**U**	**K.ILTFDQLALESPK.G**

59. O70351 **Mass:** 27343 **Score:** 119 **Matches:** 2(2) **Sequences:** 2(2) **emPAI:** 0.35

3-hydroxyacyl-CoA dehydrogenase type-2 OS=Rattus norvegicus GN=Hsd17b10 PE=1 SV=3

Query	Observed	Mr(expt)	Mr(calc)	ppm	Miss	Score	Expect	Rank	Unique	Peptide
868	**522.31**	**1042.60**	**1042.60**	**-2.65**	**0**	**26**	**0.018**	**1**	**U**	**K.EVQAALTLAK.E**
3193	**689.74**	**2066.21**	**2066.20**	**1.17**	**0**	**93**	**5.4e-010**	**1**	**U**	**R.VVTIAPGLFATPLLTTLPDK.V**

60. P85834 **Mass:** 49890 **Score:** 118 **Matches:** 2(2) **Sequences:** 2(2) **emPAI:** 0.18

Elongation factor Tu, mitochondrial OS=Rattus norvegicus GN=Tufm PE=1 SV=1

Query	Observed	Mr(expt)	Mr(calc)	ppm	Miss	Score	Expect	Rank	Unique	Peptide
1376	**593.30**	**1184.59**	**1184.61**	**-21.53**	**0**	**51**	**5.8e-005**	**1**	**U**	**R.AEAGDNLGALVR.G**
2444	**786.94**	**1571.86**	**1571.86**	**0.55**	**0**	**68**	**8.6e-007**	**1**	**U**	**K.LLDAVDTYIPVPTR.D**

61. Q9Z1B2 **Mass:** 27067 **Score:** 117 **Matches:** 2(2) **Sequences:** 2(2) **emPAI:** 0.36

Glutathione S-transferase Mu 5 OS=Rattus norvegicus GN=Gstm5 PE=1 SV=3

Query	Observed	Mr(expt)	Mr(calc)	ppm	Miss	Score	Expect	Rank	Unique	Peptide
791	508.29	1014.57	1014.58	-7.77	0	71	5.8e-007	1		K.ITQSNAILR.Y
803	**510.78**	**1019.54**	**1019.54**	**-2.60**	**0**	**46**	**0.00023**	**1**	**U**	**K.IAAFLQSDR.C**

62. P16036 **Mass:** 39876 **Score:** 116 **Matches:** 2(2) **Sequences:** 2(2) **emPAI:** 0.23

Phosphate carrier protein, mitochondrial OS=Rattus norvegicus GN=Slc25a3 PE=1 SV=1

Query	Observed	Mr(expt)	Mr(calc)	ppm	Miss	Score	Expect	Rank	Unique	Peptide
845	**518.76**	**1035.51**	**1035.51**	**-1.38**	**0**	**44**	**0.0003**	**1**	**U**	**K.FGFYEVFK.A**
891	**523.78**	**1045.55**	**1045.55**	**-4.03**	**0**	**72**	**6.4e-007**	**1**	**U**	**K.GSTASQVLQR.L**

63. Q6AYQ8 **Mass:** 24750 **Score:** 116 **Matches:** 2(2) **Sequences:** 2(2) **emPAI:** 0.39

Acylpyruvase FAHD1, mitochondrial OS=Rattus norvegicus GN=Fahd1 PE=2 SV=1

Query	Observed	Mr(expt)	Mr(calc)	ppm	Miss	Score	Expect	Rank	Unique	Peptide
190	**409.22**	**816.43**	**816.43**	**-0.03**	**0**	**30**	**0.011**	**1**	**U**	**K.NIVCVGR.N**
2694	**856.98**	**1711.95**	**1711.96**	**-8.31**	**0**	**86**	**9.6e-009**	**1**	**U**	**K.IITLEEGDLILTGTPK.G**

64. P02770 **Mass:** 70682 **Score:** 111 **Matches:** 3(3) **Sequences:** 3(3) **emPAI:** 0.19

Serum albumin OS=Rattus norvegicus GN=Alb PE=1 SV=2

Query	Observed	Mr(expt)	Mr(calc)	ppm	Miss	Score	Expect	Rank	Unique	Peptide
459	**449.75**	**897.48**	**897.47**	**2.11**	**0**	**36**	**0.0017**	**1**	**U**	**R.LCVLHEK.T**
1254	**575.31**	**1148.60**	**1148.61**	**-2.68**	**0**	**30**	**0.0084**	**1**	**U**	**K.LVQEVTDFAK.T**
2758	**583.89**	**1748.65**	**1748.66**	**-0.42**	**0**	**45**	**3.2e-005**	**1**	**U**	**K.ECCHGDLLECADDR.A**

65. Q63610 **Mass:** 29217 **Score:** 102 **Matches:** 3(3) **Sequences:** 3(3) **emPAI:** 0.53

Tropomyosin alpha-3 chain OS=Rattus norvegicus GN=Tpm3 PE=1 SV=2

Query	Observed	Mr(expt)	Mr(calc)	ppm	Miss	Score	Expect	Rank	Unique	Peptide
60	372.73	743.45	743.45	0.82	0	44	0.00032	1		R.LATALQK.L
1203	566.31	1130.60	1130.60	1.22	0	26	0.021	1	U	K.MELQEIQLK.E
2714	576.63	1726.88	1726.88	-2.04	1	32	0.0029	1		R.IQLVEEELDRAQER.L

66. P09626 **Mass:** 115675 **Score:** 98 **Matches:** 2(2) **Sequences:** 2(2) **emPAI:** 0.07

Potassium-transporting ATPase alpha chain 1 OS=Rattus norvegicus GN=Atp4a PE=2 SV=3

Query	Observed	Mr(expt)	Mr(calc)	ppm	Miss	Score	Expect	Rank	Unique	Peptide
915	528.82	1055.63	1055.63	-3.43	0	56	1e-005	1	U	K.ASLAAELLLR.D
2368	514.90	1541.68	1541.68	0.53	0	42	0.00019	1	U	R.SPECTHESPLETR.N

---

67. P70580 **Mass:** 21699 **Score:** 96 **Matches:** 3(3) **Sequences:** 3(3) **emPAI:** 0.77

Membrane-associated progesterone receptor component 1 OS=Rattus norvegicus GN=Pgrmc1 PE=1 SV=3

Query	Observed	Mr(expt)	Mr(calc)	ppm	Miss	Score	Expect	Rank	Unique	Peptide
387	438.25	874.49	874.49	-4.86	0	28	0.014	1	U	R.ILMAINGK.V
813	512.77	1023.53	1023.51	22.5	0	42	0.00045	1	U	R.GLATFCLDK.E
1117	369.18	1104.52	1104.53	-7.39	1	26	0.016	1	U	R.RYDGVQDPR.I

---

68. O35760 **Mass:** 26721 **Score:** 93 **Matches:** 3(3) **Sequences:** 3(3) **emPAI:** 0.59

Isopentenyl-diphosphate Delta-isomerase 1 OS=Rattus norvegicus GN=Idi1 PE=2 SV=2

Query	Observed	Mr(expt)	Mr(calc)	ppm	Miss	Score	Expect	Rank	Unique	Peptide
851	519.30	1036.59	1036.60	-2.72	0	31	0.0026	1	U	K.IIADAFLFK.W
1588	419.53	1255.56	1255.56	-0.14	0	35	0.0013	1	U	K.NCHLNENIDK.G
1995	708.86	1415.71	1415.71	2.23	0	27	0.015	2	U	R.AFSVFLFNTENK.L

---

69. Q63507 **Mass:** 23438 **Score:** 91 **Matches:** 2(2) **Sequences:** 2(2) **emPAI:** 0.42

60S ribosomal protein L14 OS=Rattus norvegicus GN=Rpl14 PE=1 SV=3

Query	Observed	Mr(expt)	Mr(calc)	ppm	Miss	Score	Expect	Rank	Unique	Peptide
9	353.70	705.38	705.38	-3.51	0	34	0.0033	1	U	R.FVEVGR.V
213	414.75	827.48	827.49	-6.02	0	57	1.3e-005	1	U	K.AAIAAAAAAK.A

---

70. P21670 **Mass:** 29764 **Score:** 91 **Matches:** 2(2) **Sequences:** 2(2) **emPAI:** 0.32

Proteasome subunit alpha type-4 OS=Rattus norvegicus GN=Psma4 PE=1 SV=1

Query	Observed	Mr(expt)	Mr(calc)	ppm	Miss	Score	Expect	Rank	Unique	Peptide
766	504.26	1006.50	1006.51	-10.41	0	43	0.0005	1	U	R.TTIFSPEGR.L
1494	613.83	1225.64	1225.62	13.5	0	49	9.4e-005	1	U	K.LLDEVFFSEK.I

---

71. Q29TV8 **Mass:** 20641 **Score:** 88 **Matches:** 2(2) **Sequences:** 1(1) **emPAI:** 0.22

Gastrokine-2 OS=Rattus norvegicus GN=Gkn2 PE=2 SV=1

Query	Observed	Mr(expt)	Mr(calc)	ppm	Miss	Score	Expect	Rank	Unique	Peptide
2522	403.22	1608.86	1608.86	-2.26	1	(55)	1.3e-005	1	U	K.HIPLYEGEVATKPR.E
2523	537.30	1608.86	1608.86	0.95	1	88	6e-009	1	U	K.HIPLYEGEVATKPR.E

---

72. P08699 **Mass:** 27241 **Score:** 86 **Matches:** 1(1) **Sequences:** 1(1) **emPAI:** 0.16

Galectin-3 OS=Rattus norvegicus GN=Lgals3 PE=1 SV=4

Query	Observed	Mr(expt)	Mr(calc)	ppm	Miss	Score	Expect	Rank	Unique	Peptide
2631	420.23	1676.87	1676.87	0.03	0	86	1.4e-008	1	U	K.VAVNDVHLLQYNHR.M

---

73. Q66H80 **Mass:** 57619 **Score:** 83 **Matches:** 2(2) **Sequences:** 2(2) **emPAI:** 0.15

Coatomer subunit delta OS=Rattus norvegicus GN=Arcn1 PE=2 SV=1

Query	Observed	Mr(expt)	Mr(calc)	ppm	Miss	Score	Expect	Rank	Unique	Peptide
1513	412.23	1233.68	1233.68	-2.91	1	33	0.0024	1	U	K.VAPAPARPSGPSK.A
1995	708.86	1415.71	1415.73	-9.83	0	50	7.9e-005	1	U	K.NSNILEDLETLR.L

---

74. P34064 **Mass:** 26545 **Score:** 79 **Matches:** 2(2) **Sequences:** 2(2) **emPAI:** 0.36

Proteasome subunit alpha type-5 OS=Rattus norvegicus GN=Psma5 PE=2 SV=1

Query	Observed	Mr(expt)	Mr(calc)	ppm	Miss	Score	Expect	Rank	Unique	Peptide
111	387.26	772.50	772.51	-7.13	0	30	0.0012	1	U	K.SSLIILK.Q
935	532.26	1062.50	1062.51	-6.35	0	49	8.9e-005	1	U	R.GVNTFSPEGR.L

---

75. Q8K4G6 **Mass:** 29081 **Score:** 73 **Matches:** 1(1) **Sequences:** 1(1) **emPAI:** 0.15

O-acetyl-ADP-ribose deacetylase MACROD1 (Fragment) OS=Rattus norvegicus GN=Macrod1 PE=2 SV=2

Query	Observed	Mr(expt)	Mr(calc)	ppm	Miss	Score	Expect	Rank	Unique	Peptide
2676	569.29	1704.85	1704.85	-2.29	0	73	2.7e-007	1	U	R.SCYLSSLDLLLEHR.L

---

76. P18422 **Mass:** 28629 **Score:** 69 **Matches:** 2(2) **Sequences:** 2(2) **emPAI:** 0.33

Proteasome subunit alpha type-3 OS=Rattus norvegicus GN=Psma3 PE=1 SV=3

Query	Observed	Mr(expt)	Mr(calc)	ppm	Miss	Score	Expect	Rank	Unique	Peptide
61	373.21	744.41	744.41	-1.30	0	46	0.00072	1	U	R.SLADIAR.E
1466	609.32	1216.63	1216.64	-7.28	0	24	0.035	1	U	K.AVENSSTAIGIR.C

---

77. Q6URK4 **Mass:** 39856 **Score:** 69 **Matches:** 2(2) **Sequences:** 2(2) **emPAI:** 0.23

Heterogeneous nuclear ribonucleoprotein A3 OS=Rattus norvegicus GN=Hnrnpa3 PE=1 SV=1

Query	Observed	Mr(expt)	Mr(calc)	ppm	Miss	Score	Expect	Rank	Unique	Peptide
2532	405.95	1619.75	1619.75	0.27	1	38	0.0008	1	U	R.GEEGHDPKEPEQLR.K
2696	857.41	1712.80	1712.77	18.7	0	31	0.0043	1	U	R.GFAFVTFDDHDTVDK.I

---

78. P83732 **Mass:** 17882 **Score:** 69 **Matches:** 1(1) **Sequences:** 1(1) **emPAI:** 0.26

60S ribosomal protein L24 OS=Rattus norvegicus GN=Rpl24 PE=2 SV=1

Query	Observed	Mr(expt)	Mr(calc)	ppm	Miss	Score	Expect	Rank	Unique	Peptide
1639	639.34	1276.67	1276.67	0.43	0	69	1.8e-006	1	U	R.AITGASLADIMAK.R

---

79. Q5XI73 **Mass:** 23450 **Score:** 68 **Matches:** 2(2) **Sequences:** 2(2) **emPAI:** 0.42

Rho GDP-dissociation inhibitor 1 OS=Rattus norvegicus GN=Arhgdia PE=1 SV=1

Query	Observed	Mr(expt)	Mr(calc)	ppm	Miss	Score	Expect	Rank	Unique	Peptide
605	475.28	948.54	948.54	0.31	1	33	0.0025	1	U	K.YKEALLGR.V
2588	825.96	1649.91	1649.91	1.69	0	36	0.0009	1	U	R.VAVSADPNVPNVIVTR.L

80. P11348 **Mass:** 25764 **Score:** 66 **Matches:** 1(1) **Sequences:** 1(1) **emPAI:** 0.17
Dihydropteridine reductase OS=Rattus norvegicus GN=Qdpr PE=1 SV=1

Query	Observed	Mr(expt)	Mr(calc)	ppm	Miss	Score	Expect	Rank	Unique	Peptide
1879	684.86	1367.70	1367.70	1.89	0	66	1.6e-006	1	U	K.GAVHQLCQSLAGK.N

81. P22062 **Mass:** 24683 **Score:** 66 **Matches:** 2(2) **Sequences:** 2(2) **emPAI:** 0.40
Protein-L-isoaspartate(D-aspartate) O-methyltransferase OS=Rattus norvegicus GN=Pcmt1 PE=1 SV=2

Query	Observed	Mr(expt)	Mr(calc)	ppm	Miss	Score	Expect	Rank	Unique	Peptide
18	358.21	714.40	714.40	-8.12	0	39	0.0012	1	U	R.LVVGDGR.M
2172	370.19	1476.74	1476.74	0.23	0	27	0.013	1	U	K.SGGASHSELIHNLR.K

82. P19511 **Mass:** 28965 **Score:** 65 **Matches:** 2(2) **Sequences:** 2(2) **emPAI:** 0.33
ATP synthase F(0) complex subunit B1, mitochondrial OS=Rattus norvegicus GN=Atp5f1 PE=1 SV=1

Query	Observed	Mr(expt)	Mr(calc)	ppm	Miss	Score	Expect	Rank	Unique	Peptide
8	353.19	704.36	704.35	9.23	0	23	0.058	1	U	K.CIGDLK.M
630	479.26	956.50	956.50	-2.17	0	42	0.00033	1	U	K.QIQDAINR.E

83. P62630 **Mass:** 50424 **Score:** 63 **Matches:** 1(1) **Sequences:** 1(1) **emPAI:** 0.09
Elongation factor 1-alpha 1 OS=Rattus norvegicus GN=Eef1a1 PE=1 SV=1

Query	Observed	Mr(expt)	Mr(calc)	ppm	Miss	Score	Expect	Rank	Unique	Peptide
818	513.30	1024.59	1024.60	-11.30	0	63	1.7e-006	1	U	K.IGGIGTVPVGR.V

Proteins matching the same set of peptides:
P62632 **Mass:** 50764 **Score:** 63 **Matches:** 1(1) **Sequences:** 1(1)
Elongation factor 1-alpha 2 OS=Rattus norvegicus GN=Eef1a2 PE=2 SV=1

84. P61314 **Mass:** 24245 **Score:** 61 **Matches:** 2(1) **Sequences:** 2(1) **emPAI:** 0.19
60S ribosomal protein L15 OS=Rattus norvegicus GN=Rpl15 PE=1 SV=2

Query	Observed	Mr(expt)	Mr(calc)	ppm	Miss	Score	Expect	Rank	Unique	Peptide
796	509.77	1017.53	1017.51	23.8	0	21	0.083	1	U	R.SLQSVAEER.A
2678	427.23	1704.91	1704.91	0.09	1	39	0.0005	1	U	K.GATYGKPVHHGVNQLK.F

85. P60901 **Mass:** 27838 **Score:** 57 **Matches:** 2(2) **Sequences:** 2(2) **emPAI:** 0.34
Proteasome subunit alpha type-6 OS=Rattus norvegicus GN=Psma6 PE=1 SV=1

Query	Observed	Mr(expt)	Mr(calc)	ppm	Miss	Score	Expect	Rank	Unique	Peptide
1469	406.88	1217.63	1217.64	-13.47	1	28	0.014	1	U	R.GKDCAVIVTQK.K
1747	660.36	1978.06	1978.04	11.1	1	29	0.013	1	U	R.ILTEAEIDAHLVALAERD.-

86. Q4KM65 **Mass:** 26281 **Score:** 56 **Matches:** 2(1) **Sequences:** 2(1) **emPAI:** 0.17
Cleavage and polyadenylation specificity factor subunit 5 OS=Rattus norvegicus GN=Nudt21 PE=2 SV=1

Query	Observed	Mr(expt)	Mr(calc)	ppm	Miss	Score	Expect	Rank	Unique	Peptide
1155	559.32	1116.63	1116.65	-24.08	0	36	0.0017	1	U	K.LFLVQLQEK.A
2751	873.45	1744.89	1744.90	-5.76	0	21	0.053	1	U	R.TINLYPLTNYTFGTK.E

87. Q00715 **Mass:** 13982 **Score:** 55 **Matches:** 2(2) **Sequences:** 2(2) **emPAI:** 0.79
Histone H2B type 1 OS=Rattus norvegicus PE=1 SV=2

Query	Observed	Mr(expt)	Mr(calc)	ppm	Miss	Score	Expect	Rank	Unique	Peptide
621	477.30	952.59	952.60	-5.94	0	34	0.00052	1	U	R.LLLPGELAK.H
1311	390.20	1167.58	1167.59	-6.07	0	21	0.051	1	U	K.QVHPDTGISSK.A

Proteins matching the same set of peptides:
Q00729 **Mass:** 14273 **Score:** 55 **Matches:** 2(2) **Sequences:** 2(2)
Histone H2B type 1-A OS=Rattus norvegicus GN=Hist1h2ba PE=1 SV=2

88. Q6MG61 **Mass:** 27306 **Score:** 52 **Matches:** 1(1) **Sequences:** 1(1) **emPAI:** 0.16
Chloride intracellular channel protein 1 OS=Rattus norvegicus GN=Clic1 PE=1 SV=1

Query	Observed	Mr(expt)	Mr(calc)	ppm	Miss	Score	Expect	Rank	Unique	Peptide
1650	641.34	1280.66	1280.66	-1.50	0	52	9.4e-005	1	U	K.GVTFNVTTVDTK.R

89. Q6PDV7 **Mass:** 25044 **Score:** 52 **Matches:** 2(2) **Sequences:** 2(2) **emPAI:** 0.39
60S ribosomal protein L10 OS=Rattus norvegicus GN=Rpl10 PE=1 SV=3

Query	Observed	Mr(expt)	Mr(calc)	ppm	Miss	Score	Expect	Rank	Unique	Peptide
24	360.71	719.40	719.40	6.85	0	28	0.0098	1	U	R.IFDLGR.K
695	490.71	979.41	979.42	-7.31	0	24	0.0078	1	U	K.MLSCAGADR.L

90. Q68FT1 **Mass:** 35295 **Score:** 52 **Matches:** 1(1) **Sequences:** 1(1) **emPAI:** 0.12
Ubiquinone biosynthesis protein COQ9, mitochondrial OS=Rattus norvegicus GN=Coq9 PE=2 SV=2

Query	Observed	Mr(expt)	Mr(calc)	ppm	Miss	Score	Expect	Rank	Unique	Peptide
1532	413.55	1237.63	1237.63	-1.76	0	52	4.3e-005	1	U	R.LNHVLEEEQK.L

91. P52555 **Mass:** 28614 **Score:** 51 **Matches:** 2(2) **Sequences:** 2(2) **emPAI:** 0.33
Endoplasmic reticulum resident protein 29 OS=Rattus norvegicus GN=Erp29 PE=1 SV=2

Query	Observed	Mr(expt)	Mr(calc)	ppm	Miss	Score	Expect	Rank	Unique	Peptide
841	517.80	1033.59	1033.59	-3.66	0	28	0.0065	1	U	R.SLNILTAFR.K

1749	660.83	1319.65	1319.66	-2.01	0	22	0.045	1	U	K.ESYPVFYLFR.D

---

92. Q68G31 **Mass:** 31952 **Score:** 50 **Matches:** 1(1) **Sequences:** 1(1) **emPAI:** 0.14
Phenazine biosynthesis-like domain-containing protein OS=Rattus norvegicus GN=Pbld PE=2 SV=1

Query	Observed	Mr(expt)	Mr(calc)	ppm	Miss	Score	Expect	Rank	Unique	Peptide
1918	462.25	1383.72	1383.71	5.02	1	50	5.5e-005	1	U	R.GGELDISLRPDGR.V

---

93. P30713 **Mass:** 27593 **Score:** 50 **Matches:** 2(2) **Sequences:** 2(2) **emPAI:** 0.35
Glutathione S-transferase theta-2 OS=Rattus norvegicus GN=Gstt2 PE=1 SV=3

Query	Observed	Mr(expt)	Mr(calc)	ppm	Miss	Score	Expect	Rank	Unique	Peptide
175	406.24	810.46	810.46	-3.64	0	27	0.005	1	U	R.AVYIFAK.K
580	472.77	943.53	943.52	3.19	0	23	0.059	1	U	K.NGIPFQLR.T

---

94. P62243 **Mass:** 24475 **Score:** 49 **Matches:** 1(1) **Sequences:** 1(1) **emPAI:** 0.18
40S ribosomal protein S8 OS=Rattus norvegicus GN=Rps8 PE=1 SV=2

Query	Observed	Mr(expt)	Mr(calc)	ppm	Miss	Score	Expect	Rank	Unique	Peptide
476	452.26	902.50	902.50	-1.86	0	49	0.0001	1	U	K.LLACIASR.P

---

95. Q5I0K8 **Mass:** 28350 **Score:** 49 **Matches:** 1(1) **Sequences:** 1(1) **emPAI:** 0.16
28S ribosomal protein S7, mitochondrial OS=Rattus norvegicus GN=Mrps7 PE=2 SV=2

Query	Observed	Mr(expt)	Mr(calc)	ppm	Miss	Score	Expect	Rank	Unique	Peptide
1630	638.31	1274.60	1274.61	-7.96	0	49	8.7e-005	1	U	R.AASAEEQATIER.N

---

96. Q7TP52 **Mass:** 28227 **Score:** 48 **Matches:** 1(1) **Sequences:** 1(1) **emPAI:** 0.16
Carboxymethylenebutenolidase homolog OS=Rattus norvegicus GN=Cmbl PE=2 SV=1

Query	Observed	Mr(expt)	Mr(calc)	ppm	Miss	Score	Expect	Rank	Unique	Peptide
153	401.23	800.44	800.44	2.02	0	48	0.00015	1	U	R.EVDAVLR.Y

---

97. Q9Z0V5 **Mass:** 31216 **Score:** 48 **Matches:** 1(1) **Sequences:** 1(1) **emPAI:** 0.14
Peroxiredoxin-4 OS=Rattus norvegicus GN=Prdx4 PE=2 SV=1

Query	Observed	Mr(expt)	Mr(calc)	ppm	Miss	Score	Expect	Rank	Unique	Peptide
1493	613.35	1224.68	1224.68	0.94	0	48	6.8e-005	1	U	R.QITLNDLPVGR.S

---

98. Q63716 **Mass:** 22323 **Score:** 48 **Matches:** 1(1) **Sequences:** 1(1) **emPAI:** 0.20
Peroxiredoxin-1 OS=Rattus norvegicus GN=Prdx1 PE=1 SV=1

Query	Observed	Mr(expt)	Mr(calc)	ppm	Miss	Score	Expect	Rank	Unique	Peptide
1493	613.35	1224.68	1224.68	0.94	0	48	6.8e-005	1	U	R.QITINDLPVGR.S

---

99. P62824 **Mass:** 26084 **Score:** 48 **Matches:** 1(1) **Sequences:** 1(1) **emPAI:** 0.17
Ras-related protein Rab-3C OS=Rattus norvegicus GN=Rab3c PE=1 SV=1

Query	Observed	Mr(expt)	Mr(calc)	ppm	Miss	Score	Expect	Rank	Unique	Peptide
1107	550.84	1099.66	1099.66	-2.12	0	48	5.9e-005	1	U	K.LLIIGNSSVGK.T

Proteins matching the same set of peptides:
Q63941 **Mass:** 24997 **Score:** 48 **Matches:** 1(1) **Sequences:** 1(1)
Ras-related protein Rab-3B OS=Rattus norvegicus GN=Rab3b PE=1 SV=2

---

100. P63012 **Mass:** 25182 **Score:** 48 **Matches:** 1(1) **Sequences:** 1(1) **emPAI:** 0.18
Ras-related protein Rab-3A OS=Rattus norvegicus GN=Rab3a PE=1 SV=1

Query	Observed	Mr(expt)	Mr(calc)	ppm	Miss	Score	Expect	Rank	Unique	Peptide
1107	550.84	1099.66	1099.66	-2.12	0	48	5.9e-005	1	U	K.ILIIGNSSVGK.T

---

101. Q63942 **Mass:** 24503 **Score:** 48 **Matches:** 1(1) **Sequences:** 1(1) **emPAI:** 0.18
GTP-binding protein Rab-3D OS=Rattus norvegicus GN=Rab3d PE=1 SV=2

Query	Observed	Mr(expt)	Mr(calc)	ppm	Miss	Score	Expect	Rank	Unique	Peptide
1107	550.84	1099.66	1099.66	-2.12	0	48	5.9e-005	1	U	K.LLLIGNSSVGK.T

---

102. P04256 **Mass:** 34362 **Score:** 48 **Matches:** 1(1) **Sequences:** 1(1) **emPAI:** 0.13
Heterogeneous nuclear ribonucleoprotein A1 OS=Rattus norvegicus GN=Hnrnpa1 PE=1 SV=3

Query	Observed	Mr(expt)	Mr(calc)	ppm	Miss	Score	Expect	Rank	Unique	Peptide
2041	360.19	1436.74	1436.74	1.19	1	48	0.00013	1	U	R.EDSQRPGAHLTVK.K

---

103. Q5RKI1 **Mass:** 46601 **Score:** 47 **Matches:** 1(1) **Sequences:** 1(1) **emPAI:** 0.09
Eukaryotic initiation factor 4A-II OS=Rattus norvegicus GN=Eif4a2 PE=1 SV=1

Query	Observed	Mr(expt)	Mr(calc)	ppm	Miss	Score	Expect	Rank	Unique	Peptide
1149	557.84	1113.67	1113.68	-1.12	0	47	6.4e-005	1	U	R.VLITTDLLAR.G

---

104. P13803 **Mass:** 35272 **Score:** 47 **Matches:** 1(1) **Sequences:** 1(1) **emPAI:** 0.12
Electron transfer flavoprotein subunit alpha, mitochondrial OS=Rattus norvegicus GN=Etfa PE=1 SV=4

Query	Observed	Mr(expt)	Mr(calc)	ppm	Miss	Score	Expect	Rank	Unique	Peptide
1985	705.90	1409.79	1409.81	-15.85	0	47	7.9e-005	1	U	K.LNVAPVSDIIEIK.S

---

105. Q63797 **Mass:** 28730 **Score:** 46 **Matches:** 1(1) **Sequences:** 1(1) **emPAI:** 0.15
Proteasome activator complex subunit 1 OS=Rattus norvegicus GN=Psme1 PE=2 SV=1

Query	Observed	Mr(expt)	Mr(calc)	ppm	Miss	Score	Expect	Rank	Unique	Peptide
2938	936.45	1870.89	1870.91	-7.77	0	46	0.00013	1	U	R.QLVHELDEAEYQEIR.L

106. P23640 **Mass:** 25337 **Score:** 45 **Matches:** 1(1) **Sequences:** 1(1) **emPAI:** 0.18
Ras-related protein Rab-27A OS=Rattus norvegicus GN=Rab27a PE=1 SV=1

Query	Observed	Mr(expt)	Mr(calc)	ppm	Miss	Score	Expect	Rank	Unique	Peptide
937	532.30	1062.58	1062.57	9.30	0	45	0.00018	1	U	K.FLALGDSGVGK.T

107. Q498R3 **Score:** 45 **Matches:** 1(1) **Sequences:** 1(1) **emPAI:** 0.05
DnaJ homolog subfamily C member 10 OS=Rattus norvegicus GN=Dnajc10 PE=2 SV=2

Query	Observed	Mr(expt)	Mr(calc)	ppm	Miss	Score	Expect	Rank	Unique	Peptide
153	401.23	800.44	800.44	2.02	0	45	0.00029	2	U	K.EVDGLLR.I

108. P48004 **Mass:** 28480 **Score:** 44 **Matches:** 1(1) **Sequences:** 1(1) **emPAI:** 0.16
Proteasome subunit alpha type-7 OS=Rattus norvegicus GN=Psma7 PE=1 SV=1

Query	Observed	Mr(expt)	Mr(calc)	ppm	Miss	Score	Expect	Rank	Unique	Peptide
1105	550.82	1099.62	1099.62	-1.25	0	44	0.00027	1	U	K.ALLEVVQSGGK.N

109. Q5XI72 **Mass:** 27364 **Score:** 42 **Matches:** 1(1) **Sequences:** 1(1) **emPAI:** 0.16
Eukaryotic translation initiation factor 4H OS=Rattus norvegicus GN=Eif4h PE=1 SV=1

Query	Observed	Mr(expt)	Mr(calc)	ppm	Miss	Score	Expect	Rank	Unique	Peptide
87	380.21	758.41	758.39	22.2	0	42	0.00071	1	U	R.VDIAEGR.K

110. P02773 **Mass:** 70166 **Score:** 41 **Matches:** 1(1) **Sequences:** 1(1) **emPAI:** 0.06
Alpha-fetoprotein OS=Rattus norvegicus GN=Afp PE=2 SV=1

Query	Observed	Mr(expt)	Mr(calc)	ppm	Miss	Score	Expect	Rank	Unique	Peptide
314	424.25	846.49	846.50	-6.77	1	41	0.0004	1	U	K.LSQKFPK.A

111. P17077 **Mass:** 21994 **Score:** 40 **Matches:** 1(1) **Sequences:** 1(1) **emPAI:** 0.21
60S ribosomal protein L9 OS=Rattus norvegicus GN=Rpl9 PE=1 SV=1

Query	Observed	Mr(expt)	Mr(calc)	ppm	Miss	Score	Expect	Rank	Unique	Peptide
379	437.20	872.39	872.39	-2.36	0	40	0.0003	1	U	K.GTVQQPDE.-

112. P38918 **Mass:** 37122 **Score:** 39 **Matches:** 1(1) **Sequences:** 1(1) **emPAI:** 0.12
Aflatoxin B1 aldehyde reductase member 3 OS=Rattus norvegicus GN=Akr7a3 PE=1 SV=2

Query	Observed	Mr(expt)	Mr(calc)	ppm	Miss	Score	Expect	Rank	Unique	Peptide
2491	798.93	1595.84	1595.85	-0.71	0	39	0.00068	1	U	R.FYAFNPLAGGLLTGR.Y

113. Q9JHW0 **Mass:** 30250 **Score:** 39 **Matches:** 1(1) **Sequences:** 1(1) **emPAI:** 0.15
Proteasome subunit beta type-7 OS=Rattus norvegicus GN=Psmb7 PE=1 SV=1

Query	Observed	Mr(expt)	Mr(calc)	ppm	Miss	Score	Expect	Rank	Unique	Peptide
524	460.26	918.50	918.50	0.50	0	39	0.0012	1	U	K.GTTAVLTEK.V

114. Q5XIF6 **Mass:** 50634 **Score:** 39 **Matches:** 1(1) **Sequences:** 1(1) **emPAI:** 0.09
Tubulin alpha-4A chain OS=Rattus norvegicus GN=Tuba4a PE=2 SV=1

Query	Observed	Mr(expt)	Mr(calc)	ppm	Miss	Score	Expect	Rank	Unique	Peptide
2773	586.33	1755.96	1755.96	1.25	0	39	0.00063	1	U	R.IHFPLATYAPVISAEK.A

Proteins matching the same set of peptides:
Q6AYZ1 **Mass:** 50590 **Score:** 39 **Matches:** 1(1) **Sequences:** 1(1)
Tubulin alpha-1C chain OS=Rattus norvegicus GN=Tuba1c PE=1 SV=1
Q6P9V9 **Mass:** 50804 **Score:** 39 **Matches:** 1(1) **Sequences:** 1(1)
Tubulin alpha-1B chain OS=Rattus norvegicus GN=Tuba1b PE=1 SV=1
Q68FR8 **Mass:** 50612 **Score:** 39 **Matches:** 1(1) **Sequences:** 1(1)
Tubulin alpha-3 chain OS=Rattus norvegicus GN=Tuba3a PE=2 SV=1
P68370 **Mass:** 50788 **Score:** 39 **Matches:** 1(1) **Sequences:** 1(1)
Tubulin alpha-1A chain OS=Rattus norvegicus GN=Tuba1a PE=1 SV=1

115. P62804 **Mass:** 11360 **Score:** 37 **Matches:** 1(1) **Sequences:** 1(1) **emPAI:** 0.43
Histone H4 OS=Rattus norvegicus GN=Hist1h4b PE=1 SV=2

Query	Observed	Mr(expt)	Mr(calc)	ppm	Miss	Score	Expect	Rank	Unique	Peptide
1213	378.85	1133.53	1133.54	-4.48	0	37	0.0013	1	U	R.DAVTYTEHAK.R

116. Q810U0 **Score:** 37 **Matches:** 1(1) **Sequences:** 1(1) **emPAI:** 0.13
Coiled-coil domain-containing protein 50 OS=Rattus norvegicus GN=Ccdc50 PE=2 SV=1

Query	Observed	Mr(expt)	Mr(calc)	ppm	Miss	Score	Expect	Rank	Unique	Peptide
223	416.23	830.45	830.45	-0.82	0	37	0.0024	2	U	K.LTIEAER.R

117. Q63798 **Mass:** 27068 **Score:** 36 **Matches:** 1(1) **Sequences:** 1(1) **emPAI:** 0.16
Proteasome activator complex subunit 2 OS=Rattus norvegicus GN=Psme2 PE=2 SV=3

Query	Observed	Mr(expt)	Mr(calc)	ppm	Miss	Score	Expect	Rank	Unique	Peptide
228	417.23	832.45	832.45	-1.89	0	36	0.0054	1	U	R.AMVLDLR.A

118. Q3KRE8 **Mass:** 50377 **Score:** 35 **Matches:** 1(1) **Sequences:** 1(1) **emPAI:** 0.09
Tubulin beta-2B chain OS=Rattus norvegicus GN=Tubb2b PE=1 SV=1

Query	Observed	Mr(expt)	Mr(calc)	ppm	Miss	Score	Expect	Rank	Unique	Peptide
979	359.85	1076.53	1076.53	7.54	1	35	0.0023	1	U	K.IREEYPDR.I

Proteins matching the same set of peptides:
Q6P9T8 **Mass:** 50225 **Score:** 35 **Matches:** 1(1) **Sequences:** 1(1)
Tubulin beta-4B chain OS=Rattus norvegicus GN=Tubb4b PE=1 SV=1
P69897 **Mass:** 50095 **Score:** 35 **Matches:** 1(1) **Sequences:** 1(1)
Tubulin beta-5 chain OS=Rattus norvegicus GN=Tubb5 PE=1 SV=1
P85108 **Mass:** 50274 **Score:** 35 **Matches:** 1(1) **Sequences:** 1(1)
Tubulin beta-2A chain OS=Rattus norvegicus GN=Tubb2a PE=1 SV=1

---

119. P17136 **Mass:** 21751 **Score:** 35 **Matches:** 1(1) **Sequences:** 1(1) **emPAI:** 0.21
Small nuclear ribonucleoprotein-associated protein B (Fragment) OS=Rattus norvegicus GN=Snrpb PE=2 SV=1

Query	Observed	Mr(expt)	Mr(calc)	ppm	Miss	Score	Expect	Rank	Unique	Peptide
208	413.25	824.48	824.48	0.75	0	35	0.0011	1	U	R.IFIGTFK.A

Proteins matching the same set of peptides:
P63164 **Mass:** 24769 **Score:** 35 **Matches:** 1(1) **Sequences:** 1(1)
Small nuclear ribonucleoprotein-associated protein N OS=Rattus norvegicus GN=Snrpn PE=2 SV=1

---

120. Q9QWN8 **Mass:** 272151 **Score:** 35 **Matches:** 1(1) **Sequences:** 1(1) **emPAI:** 0.02
Spectrin beta chain, non-erythrocytic 2 OS=Rattus norvegicus GN=Sptbn2 PE=1 SV=2

Query	Observed	Mr(expt)	Mr(calc)	ppm	Miss	Score	Expect	Rank	Unique	Peptide
223	416.23	830.45	830.45	-0.82	0	36	0.003	3	U	K.LTALEER.E

---

121. Q9Z0W7 **Mass:** 28843 **Score:** 34 **Matches:** 1(1) **Sequences:** 1(1) **emPAI:** 0.15
Chloride intracellular channel protein 4 OS=Rattus norvegicus GN=Clic4 PE=1 SV=3

Query	Observed	Mr(expt)	Mr(calc)	ppm	Miss	Score	Expect	Rank	Unique	Peptide
40	367.20	732.38	732.38	-6.88	0	34	0.0049	1	U	R.NFDIPK.G

---

122. O35952 **Mass:** 34544 **Score:** 34 **Matches:** 1(1) **Sequences:** 1(1) **emPAI:** 0.13
Hydroxyacylglutathione hydrolase, mitochondrial OS=Rattus norvegicus GN=Hagh PE=1 SV=2

Query	Observed	Mr(expt)	Mr(calc)	ppm	Miss	Score	Expect	Rank	Unique	Peptide
1716	436.89	1307.64	1307.65	-4.68	0	34	0.0032	1	U	R.HVEPGNTAVQEK.L

---

123. O35264 **Mass:** 25736 **Score:** 33 **Matches:** 1(1) **Sequences:** 1(1) **emPAI:** 0.17
Platelet-activating factor acetylhydrolase IB subunit beta OS=Rattus norvegicus GN=Pafah1b2 PE=1 SV=1

Query	Observed	Mr(expt)	Mr(calc)	ppm	Miss	Score	Expect	Rank	Unique	Peptide
723	497.34	992.67	992.67	-3.22	0	33	0.00047	1	U	K.IIVLGLLPR.G

---

124. Q5XI32 **Mass:** 30952 **Score:** 33 **Matches:** 1(1) **Sequences:** 1(1) **emPAI:** 0.14
F-actin-capping protein subunit beta OS=Rattus norvegicus GN=Capzb PE=1 SV=1

Query	Observed	Mr(expt)	Mr(calc)	ppm	Miss	Score	Expect	Rank	Unique	Peptide
1321	586.30	1170.58	1170.59	-12.44	0	33	0.0092	1	U	R.STLNEIYFGK.T

---

125. B0BN44 **Mass:** 43139 **Score:** 32 **Matches:** 1(1) **Sequences:** 1(1) **emPAI:** 0.10
Tumor protein p53-inducible protein 13 OS=Rattus norvegicus GN=Tp53i13 PE=2 SV=1

Query	Observed	Mr(expt)	Mr(calc)	ppm	Miss	Score	Expect	Rank	Unique	Peptide
577	472.28	942.55	942.53	24.3	0	32	0.0034	1	U	M.VPPPPPPSR.L

---

126. Q4KLH6 **Mass:** 161131 **Score:** 32 **Matches:** 1(1) **Sequences:** 1(1) **emPAI:** 0.03
Centrosomal protein of 162 kDa OS=Rattus norvegicus GN=Cep162 PE=1 SV=2

Query	Observed	Mr(expt)	Mr(calc)	ppm	Miss	Score	Expect	Rank	Unique	Peptide
1238	571.86	1141.71	1141.71	-1.19	1	32	0.00093	1	U	R.KIATQEVLLK.H

---

127. Q5U316 **Mass:** 23296 **Score:** 31 **Matches:** 1(1) **Sequences:** 1(1) **emPAI:** 0.19
Ras-related protein Rab-35 OS=Rattus norvegicus GN=Rab35 PE=1 SV=1

Query	Observed	Mr(expt)	Mr(calc)	ppm	Miss	Score	Expect	Rank	Unique	Peptide
963	536.31	1070.61	1070.63	-20.88	0	31	0.0058	1	U	K.LLIIGDSGVGK.S

---

128. Q6NYB7 **Mass:** 22891 **Score:** 31 **Matches:** 1(1) **Sequences:** 1(1) **emPAI:** 0.20
Ras-related protein Rab-1A OS=Rattus norvegicus GN=Rab1A PE=1 SV=3

Query	Observed	Mr(expt)	Mr(calc)	ppm	Miss	Score	Expect	Rank	Unique	Peptide
963	536.31	1070.61	1070.63	-20.88	0	31	0.0058	1	U	K.LLLIGDSGVGK.S

Proteins matching the same set of peptides:
P10536 **Mass:** 22320 **Score:** 31 **Matches:** 1(1) **Sequences:** 1(1)
Ras-related protein Rab-1B OS=Rattus norvegicus GN=Rab1b PE=1 SV=1
P35280 **Mass:** 23824 **Score:** 31 **Matches:** 1(1) **Sequences:** 1(1)
Ras-related protein Rab-8A OS=Rattus norvegicus GN=Rab8a PE=1 SV=2
P35281 **Mass:** 23072 **Score:** 31 **Matches:** 1(1) **Sequences:** 1(1)
Ras-related protein Rab-10 OS=Rattus norvegicus GN=Rab10 PE=1 SV=1
P35286 **Mass:** 23058 **Score:** 31 **Matches:** 1(1) **Sequences:** 1(1)
Ras-related protein Rab-13 OS=Rattus norvegicus GN=Rab13 PE=1 SV=2
P35289 **Mass:** 24610 **Score:** 31 **Matches:** 1(1) **Sequences:** 1(1)
Ras-related protein Rab-15 OS=Rattus norvegicus GN=Rab15 PE=2 SV=1
P70550 **Mass:** 23759 **Score:** 31 **Matches:** 1(1) **Sequences:** 1(1)
Ras-related protein Rab-8B OS=Rattus norvegicus GN=Rab8b PE=1 SV=1

---

129. P20294 **Score:** 30 **Matches:** 1(1) **Sequences:** 1(1) **emPAI:** 0.20
Ciliary neurotrophic factor OS=Rattus norvegicus GN=Cntf PE=1 SV=1

Query	Observed	Mr(expt)	Mr(calc)	ppm	Miss	Score	Expect	Rank	Unique	Peptide
61	373.21	744.41	744.43	-21.80	0	30	0.027	2	U	R.SIWLAR.K

130. Q5PPI1 **Mass:** 25653 **Score:** 30 **Matches:** 1(1) **Sequences:** 1(1) **emPAI:** 0.17
Serine/arginine-rich splicing factor 9 OS=Rattus norvegicus GN=Srsf9 PE=1 SV=1

Query	Observed	Mr(expt)	Mr(calc)	ppm	Miss	Score	Expect	Rank	Unique	Peptide
1352	393.52	1177.53	1177.54	-4.33	0	30	0.0047	1	U	R.SHEGETSYIR.V

131. Q9Z269 **Score:** 29 **Matches:** 1(1) **Sequences:** 1(1) **emPAI:** 0.16
Vesicle-associated membrane protein-associated protein B OS=Rattus norvegicus GN=Vapb PE=1 SV=3

Query	Observed	Mr(expt)	Mr(calc)	ppm	Miss	Score	Expect	Rank	Unique	Peptide
1641	426.87	1277.58	1277.58	-0.56	1	29	0.0052	2	U	K.EAKPEDLMDSK.L

132. O35263 **Mass:** 25961 **Score:** 29 **Matches:** 1(1) **Sequences:** 1(1) **emPAI:** 0.17
Platelet-activating factor acetylhydrolase IB subunit gamma OS=Rattus norvegicus GN=Pafah1b3 PE=2 SV=1

Query	Observed	Mr(expt)	Mr(calc)	ppm	Miss	Score	Expect	Rank	Unique	Peptide
654	483.33	964.64	964.64	-6.38	0	29	0.0011	1	U	R.VVVLGLLPR.G

133. Q711G3 **Mass:** 28386 **Score:** 28 **Matches:** 2(2) **Sequences:** 1(1) **emPAI:** 0.16
Isoamyl acetate-hydrolyzing esterase 1 homolog OS=Rattus norvegicus GN=Iah1 PE=2 SV=2

Query	Observed	Mr(expt)	Mr(calc)	ppm	Miss	Score	Expect	Rank	Unique	Peptide
189	409.22	816.42	816.43	-4.15	0	28	0.014	1	U	K.ACLQVAR.D 188

134. P20759 **Mass:** 36493 **Score:** 28 **Matches:** 1(1) **Sequences:** 1(1) **emPAI:** 0.12
Ig gamma-1 chain C region OS=Rattus norvegicus PE=1 SV=1

Query	Observed	Mr(expt)	Mr(calc)	ppm	Miss	Score	Expect	Rank	Unique	Peptide
1106	550.83	1099.64	1099.65	-4.67	0	28	0.008	1	U	K.DVLTITLTPK.V

Proteins matching the same set of peptides:
P20760 **Mass:** 35677 **Score:** 28 **Matches:** 1(1) **Sequences:** 1(1)
Ig gamma-2A chain C region OS=Rattus norvegicus GN=Igg-2a PE=1 SV=1

135. Q6AYK6 **Mass:** 26639 **Score:** 28 **Matches:** 1(1) **Sequences:** 1(1) **emPAI:** 0.17
Calcyclin-binding protein OS=Rattus norvegicus GN=Cacybp PE=1 SV=1

Query	Observed	Mr(expt)	Mr(calc)	ppm	Miss	Score	Expect	Rank	Unique	Peptide
1064	363.87	1088.58	1088.58	-5.85	1	28	0.013	1	U	R.LRDTLTNEK.S

136. Q923M1 **Mass:** 26120 **Score:** 28 **Matches:** 1(1) **Sequences:** 1(1) **emPAI:** 0.17
Mitochondrial peptide methionine sulfoxide reductase OS=Rattus norvegicus GN=Msra PE=1 SV=1

Query	Observed	Mr(expt)	Mr(calc)	ppm	Miss	Score	Expect	Rank	Unique	Peptide
1234	571.31	1140.61	1140.61	-7.24	0	28	0.01	1	U	K.VISAEEALPGR.T

137. P07896 **Score:** 28 **Matches:** 1(1) **Sequences:** 1(1) **emPAI:** 0.05
Peroxisomal bifunctional enzyme OS=Rattus norvegicus GN=Ehhadh PE=1 SV=2

Query	Observed	Mr(expt)	Mr(calc)	ppm	Miss	Score	Expect	Rank	Unique	Peptide
753	503.24	1004.47	1004.50	-24.59	0	28	0.011	2	U	R.ILEEGMAAR.P

138. O35244 **Mass:** 24860 **Score:** 27 **Matches:** 1(1) **Sequences:** 1(1) **emPAI:** 0.18
Peroxiredoxin-6 OS=Rattus norvegicus GN=Prdx6 PE=1 SV=3

Query	Observed	Mr(expt)	Mr(calc)	ppm	Miss	Score	Expect	Rank	Unique	Peptide
1950	698.33	1394.65	1394.65	0.48	0	27	0.0092	1	U	R.DFTPVCTTELGR.A

139. Q9JKS6 **Mass:** 554201 **Score:** 27 **Matches:** 1(1) **Sequences:** 1(1) **emPAI:** 0.01
Protein piccolo OS=Rattus norvegicus GN=Pclo PE=1 SV=1

Query	Observed	Mr(expt)	Mr(calc)	ppm	Miss	Score	Expect	Rank	Unique	Peptide
788	507.81	1013.61	1013.61	-1.94	1	27	0.0096	1	U	R.VDAKVEIIK.H

140. Q4V8Q1 **Mass:** 83985 **Score:** 27 **Matches:** 1(1) **Sequences:** 1(1) **emPAI:** 0.05
Calpain 11 OS=Rattus norvegicus GN=Capn11 PE=2 SV=2

Query	Observed	Mr(expt)	Mr(calc)	ppm	Miss	Score	Expect	Rank	Unique	Peptide
368	435.74	869.47	869.45	21.0	0	27	0.01	1	U	R.IQNPWGR.I

141. P24268 **Mass:** 45165 **Score:** 27 **Matches:** 1(1) **Sequences:** 1(1) **emPAI:** 0.10
Cathepsin D OS=Rattus norvegicus GN=Ctsd PE=1 SV=1

Query	Observed	Mr(expt)	Mr(calc)	ppm	Miss	Score	Expect	Rank	Unique	Peptide
1115	552.84	1103.66	1103.66	-3.18	0	27	0.0044	1	U	K.VSSLPIITFK.L

142. Q3KR99 **Mass:** 69640 **Score:** 26 **Matches:** 1(1) **Sequences:** 1(1) **emPAI:** 0.06
Protein Spindly OS=Rattus norvegicus GN=Spdl1 PE=2 SV=1

Query	Observed	Mr(expt)	Mr(calc)	ppm	Miss	Score	Expect	Rank	Unique	Peptide
36	365.73	729.44	729.44	6.54	1	26	0.017	1	U	K.LDKLNK.E

143. P16086 **Mass:** 285261 **Score:** 26 **Matches:** 1(1) **Sequences:** 1(1) **emPAI:** 0.01
Spectrin alpha chain, non-erythrocytic 1 OS=Rattus norvegicus GN=Sptan1 PE=1 SV=2

Query	Observed	Mr(expt)	Mr(calc)	ppm	Miss	Score	Expect	Rank	Unique	Peptide
36	365.73	729.44	729.44	6.54	1	26	0.017	1	U	R.LKDINK.V

144. P68101 **Mass:** 36371 **Score:** 26 **Matches:** 1(1) **Sequences:** 1(1) **emPAI:** 0.12

Eukaryotic translation initiation factor 2 subunit 1 OS=Rattus norvegicus GN=Eif2s1 PE=1 SV=2

Query	Observed	Mr(expt)	Mr(calc)	ppm	Miss	Score	Expect	Rank	Unique	Peptide
36	365.73	729.44	729.45	-8.86	1	26	0.017	1	U	R.IRSINK.L

---

145. Q6AY09 **Mass:** 49547 **Score:** 26 **Matches:** 1(1) **Sequences:** 1(1) **emPAI:** 0.09
Heterogeneous nuclear ribonucleoprotein H2 OS=Rattus norvegicus GN=Hnrnph2 PE=1 SV=1

Query	Observed	Mr(expt)	Mr(calc)	ppm	Miss	Score	Expect	Rank	Unique	Peptide
1182	562.26	1683.76	1683.76	-2.22	0	26	0.024	1	U	K.HTGPNSPDTANDGFVR.L

Proteins matching the same set of peptides:
Q8VHV7 **Mass:** 49442 **Score:** 26 **Matches:** 1(1) **Sequences:** 1(1)
Heterogeneous nuclear ribonucleoprotein H OS=Rattus norvegicus GN=Hnrnph1 PE=1 SV=2

---

146. P97532 **Mass:** 33205 **Score:** 26 **Matches:** 1(1) **Sequences:** 1(1) **emPAI:** 0.13
3-mercaptopyruvate sulfurtransferase OS=Rattus norvegicus GN=Mpst PE=1 SV=3

Query	Observed	Mr(expt)	Mr(calc)	ppm	Miss	Score	Expect	Rank	Unique	Peptide
920	530.26	1058.51	1058.51	-0.21	0	26	0.018	1	U	R.FQGTQPEPR.D

---

147. Q5RJR8 **Mass:** 35304 **Score:** 26 **Matches:** 1(1) **Sequences:** 1(1) **emPAI:** 0.12
Leucine-rich repeat-containing protein 59 OS=Rattus norvegicus GN=Lrrc59 PE=1 SV=1

Query	Observed	Mr(expt)	Mr(calc)	ppm	Miss	Score	Expect	Rank	Unique	Peptide
2817	596.65	1786.92	1786.93	-3.71	0	26	0.016	1	U	K.LSTLPSDFCGLTHLVK.L

---

148. P27605 **Mass:** 24690 **Score:** 26 **Matches:** 1(1) **Sequences:** 1(1) **emPAI:** 0.18
Hypoxanthine-guanine phosphoribosyltransferase OS=Rattus norvegicus GN=Hprt1 PE=1 SV=1

Query	Observed	Mr(expt)	Mr(calc)	ppm	Miss	Score	Expect	Rank	Unique	Peptide
1634	638.34	1274.66	1274.67	-10.16	0	26	0.019	1	U	K.VIGGDDLSTLTGK.N

---

149. P11232 **Mass:** 12008 **Score:** 26 **Matches:** 1(1) **Sequences:** 1(1) **emPAI:** 0.40
Thioredoxin OS=Rattus norvegicus GN=Txn PE=1 SV=2

Query	Observed	Mr(expt)	Mr(calc)	ppm	Miss	Score	Expect	Rank	Unique	Peptide
495	454.73	907.44	907.44	-3.23	0	26	0.022	1	U	K.VGEFSGANK.E

---

150. Q505J8 **Mass:** 57798 **Score:** 25 **Matches:** 1(1) **Sequences:** 1(1) **emPAI:** 0.07
Phenylalanine--tRNA ligase alpha subunit OS=Rattus norvegicus GN=Farsa PE=1 SV=1

Query	Observed	Mr(expt)	Mr(calc)	ppm	Miss	Score	Expect	Rank	Unique	Peptide
1613	634.35	1266.69	1266.69	0.44	0	25	0.016	1	U	M.ADNPVLEQLLR.R

---

151. Q32PZ3 **Mass:** 104373 **Score:** 25 **Matches:** 1(1) **Sequences:** 1(1) **emPAI:** 0.04
Protein unc-45 homolog A OS=Rattus norvegicus GN=Unc45a PE=2 SV=1

Query	Observed	Mr(expt)	Mr(calc)	ppm	Miss	Score	Expect	Rank	Unique	Peptide
9	353.70	705.38	705.37	7.68	0	25	0.029	2	U	K.MVELAK.Y

---

152. Q5I0E7 **Mass:** 27182 **Score:** 25 **Matches:** 1(1) **Sequences:** 1(1) **emPAI:** 0.16
Transmembrane emp24 domain-containing protein 9 OS=Rattus norvegicus GN=Tmed9 PE=1 SV=1

Query	Observed	Mr(expt)	Mr(calc)	ppm	Miss	Score	Expect	Rank	Unique	Peptide
1803	671.37	1340.73	1340.73	1.34	0	25	0.016	1	U	R.QLVEQVEQIQK.E

---

153. Q64595 **Mass:** 87811 **Score:** 24 **Matches:** 1(1) **Sequences:** 1(1) **emPAI:** 0.05
cGMP-dependent protein kinase 2 OS=Rattus norvegicus GN=Prkg2 PE=1 SV=1

Query	Observed	Mr(expt)	Mr(calc)	ppm	Miss	Score	Expect	Rank	Unique	Peptide
46	367.73	733.45	733.45	-1.05	0	24	0.0063	1	U	R.LGIIYR.D

---

154. Q4G069 **Mass:** 35663 **Score:** 24 **Matches:** 1(1) **Sequences:** 1(1) **emPAI:** 0.12
Regulator of microtubule dynamics protein 1 OS=Rattus norvegicus GN=Rmdn1 PE=2 SV=1

Query	Observed	Mr(expt)	Mr(calc)	ppm	Miss	Score	Expect	Rank	Unique	Peptide
1720	656.36	1310.71	1310.71	-0.94	0	24	0.02	1	U	K.VLLYEALEYAK.R

---

155. P21575 **Mass:** 97576 **Score:** 24 **Matches:** 1(1) **Sequences:** 1(1) **emPAI:** 0.04
Dynamin-1 OS=Rattus norvegicus GN=Dnm1 PE=1 SV=2

Query	Observed	Mr(expt)	Mr(calc)	ppm	Miss	Score	Expect	Rank	Unique	Peptide
184	407.76	813.50	813.50	10.5	1	24	0.016	1	U	K.LKEPSIK.C

---

156. Q3KR73 **Mass:** 93548 **Score:** 24 **Matches:** 1(1) **Sequences:** 1(1) **emPAI:** 0.05
KAT8 regulatory NSL complex subunit 3 OS=Rattus norvegicus GN=Kansl3 PE=2 SV=1

Query	Observed	Mr(expt)	Mr(calc)	ppm	Miss	Score	Expect	Rank	Unique	Peptide
1394	595.33	1188.65	1188.65	3.04	0	24	0.03	2	U	K.SSQIGTSQLLR.R

---

157. O54939 **Mass:** 34657 **Score:** 24 **Matches:** 1(1) **Sequences:** 1(1) **emPAI:** 0.13
Testosterone 17-beta-dehydrogenase 3 OS=Rattus norvegicus GN=Hsd17b3 PE=2 SV=1

Query	Observed	Mr(expt)	Mr(calc)	ppm	Miss	Score	Expect	Rank	Unique	Peptide
1463	608.33	1214.64	1214.65	-6.59	0	24	0.031	1	U	K.LQVISEEIER.T

---

158. O88761 **Mass:** 106707 **Score:** 24 **Matches:** 1(1) **Sequences:** 1(1) **emPAI:** 0.04
26S proteasome non-ATPase regulatory subunit 1 OS=Rattus norvegicus GN=Psmd1 PE=2 SV=1

Query	Observed	Mr(expt)	Mr(calc)	ppm	Miss	Score	Expect	Rank	Unique	Peptide
1331	588.32	1174.62	1174.60	16.8	0	24	0.034	1	U	R.NNNTDLMILK.N

159. Q6AXQ7 **Score:** 23 **Matches:** 1(1) **Sequences:** 1(1) **emPAI:** 0.05
IQ and AAA domain-containing protein 1-like OS=Rattus norvegicus GN=Iqca1p1 PE=2 SV=2

Query	Observed	Mr(expt)	Mr(calc)	ppm	Miss	Score	Expect	Rank	Unique	Peptide
85	379.75	757.48	757.49	-3.62	0	23	0.021	2	U	R.ILFIPR.P

160. P41739 **Score:** 23 **Matches:** 1(1) **Sequences:** 1(1) **emPAI:** 0.05
Aryl hydrocarbon receptor nuclear translocator OS=Rattus norvegicus GN=Arnt PE=1 SV=2

Query	Observed	Mr(expt)	Mr(calc)	ppm	Miss	Score	Expect	Rank	Unique	Peptide
1793	670.36	1338.71	1338.72	-7.40	1	23	0.026	2	U	K.GQVLSVMFRFR.A

161. P08689 **Score:** 23 **Matches:** 1(1) **Sequences:** 1(1) **emPAI:** 0.10
Sex hormone-binding globulin OS=Rattus norvegicus GN=Shbg PE=1 SV=1

Query	Observed	Mr(expt)	Mr(calc)	ppm	Miss	Score	Expect	Rank	Unique	Peptide
62	373.23	744.44	744.43	15.0	0	23	0.054	2	U	R.LASLWR.L

162. Q5EBA1 **Mass:** 87620 **Score:** 23 **Matches:** 1(1) **Sequences:** 1(1) **emPAI:** 0.05
ATP-dependent RNA helicase SUPV3L1, mitochondrial OS=Rattus norvegicus GN=Supv3l1 PE=2 SV=1

Query	Observed	Mr(expt)	Mr(calc)	ppm	Miss	Score	Expect	Rank	Unique	Peptide
728	**498.80**	**995.59**	**995.61**	**-12.46**	**0**	**23**	**0.012**	**1**	**U**	**R.IIFYSLIK.P**

163. Q9Z0T0 **Mass:** 27959 **Score:** 23 **Matches:** 1(1) **Sequences:** 1(1) **emPAI:** 0.16
Thiopurine S-methyltransferase OS=Rattus norvegicus GN=Tpmt PE=2 SV=1

Query	Observed	Mr(expt)	Mr(calc)	ppm	Miss	Score	Expect	Rank	Unique	Peptide
658	**484.25**	**966.49**	**966.50**	**-7.12**	**0**	**23**	**0.035**	**1**	**U**	**R.VFFPLCGK.A**

164. F1M649 **Score:** 22 **Matches:** 1(0) **Sequences:** 1(0)
NLR family CARD domain-containing protein 4 OS=Rattus norvegicus GN=Nlrc4 PE=3 SV=2

Query	Observed	Mr(expt)	Mr(calc)	ppm	Miss	Score	Expect	Rank	Unique	Peptide
61	373.21	744.41	744.41	-1.30	0	22	0.16	3	U	K.SLAEGLR.N

165. Q5QE80 **Mass:** 148595 **Score:** 22 **Matches:** 1(1) **Sequences:** 1(1) **emPAI:** 0.03
Aldehyde oxidase 3 OS=Rattus norvegicus GN=Aox3 PE=1 SV=1

Query	Observed	Mr(expt)	Mr(calc)	ppm	Miss	Score	Expect	Rank	Unique	Peptide
245	**421.76**	**841.50**	**841.51**	**-12.40**	**0**	**22**	**0.03**	**1**	**U**	**R.GIAIIPMK.F**

166. D3ZBP4 **Score:** 22 **Matches:** 1(1) **Sequences:** 1(1) **emPAI:** 0.04
Protein-methionine sulfoxide oxidase MICAL1 OS=Rattus norvegicus GN=Mical1 PE=3 SV=1

Query	Observed	Mr(expt)	Mr(calc)	ppm	Miss	Score	Expect	Rank	Unique	Peptide
3	350.72	699.43	699.43	1.61	0	22	0.053	2	U	K.LEVALR.K

Proteins matching the same set of peptides:
Q9JMD2 **Score:** 22 **Matches:** 1(1) **Sequences:** 1(1)
Scm-like with four MBT domains protein 1 OS=Rattus norvegicus GN=Sfmbt1 PE=2 SV=2

167. Q00566 **Score:** 22 **Matches:** 1(1) **Sequences:** 1(1) **emPAI:** 0.08
Methyl-CpG-binding protein 2 OS=Rattus norvegicus GN=Mecp2 PE=1 SV=1

Query	Observed	Mr(expt)	Mr(calc)	ppm	Miss	Score	Expect	Rank	Unique	Peptide
226	416.75	831.48	831.46	24.4	0	22	0.043	2	U	M.VAGMLGLR.K

168. Q5U2Q4 **Mass:** 37088 **Score:** 22 **Matches:** 1(1) **Sequences:** 1(1) **emPAI:** 0.12
Mono [ADP-ribose] polymerase PARP16 OS=Rattus norvegicus GN=Parp16 PE=2 SV=1

Query	Observed	Mr(expt)	Mr(calc)	ppm	Miss	Score	Expect	Rank	Unique	Peptide
1872	**683.89**	**1365.76**	**1365.77**	**-3.79**	**1**	**22**	**0.021**	**1**	**U**	**K.YLLVYSQKQPK.R**

169. F1MA98 **Mass:** 267486 **Score:** 22 **Matches:** 1(0) **Sequences:** 1(0)
Nucleoprotein TPR OS=Rattus norvegicus GN=Tpr PE=1 SV=1

Query	Observed	Mr(expt)	Mr(calc)	ppm	Miss	Score	Expect	Rank	Unique	Peptide
740	**501.27**	**1000.53**	**1000.52**	**9.67**	**0**	**22**	**0.059**	**1**	**U**	**K.LQNEQLEK.L**

170. Q9ERD9 **Mass:** 46087 **Score:** 21 **Matches:** 1(0) **Sequences:** 1(0)
Indoleamine 2,3-dioxygenase 1 OS=Rattus norvegicus GN=Ido1 PE=2 SV=1

Query	Observed	Mr(expt)	Mr(calc)	ppm	Miss	Score	Expect	Rank	Unique	Peptide
1607	**633.32**	**1264.64**	**1264.62**	**15.3**	**0**	**21**	**0.069**	**1**	**U**	**M.PHSQISPAEGSR.R**

171. Q05030 **Score:** 21 **Matches:** 1(0) **Sequences:** 1(0)
Platelet-derived growth factor receptor beta OS=Rattus norvegicus GN=Pdgfrb PE=2 SV=2

Query	Observed	Mr(expt)	Mr(calc)	ppm	Miss	Score	Expect	Rank	Unique	Peptide
115	390.21	778.41	778.42	-13.10	0	21	0.076	2	U	R.LLGEGYK.K

172. Q63517 **Score:** 21 **Matches:** 1(0) **Sequences:** 1(0)
Centromere protein I OS=Rattus norvegicus GN=Cenpi PE=2 SV=1

Query	Observed	Mr(expt)	Mr(calc)	ppm	Miss	Score	Expect	Rank	Unique	Peptide
36	365.73	729.44	729.44	6.51	1	21	0.06	4	U	K.KDLVQK.A

173. Q8VIL3 **Score:** 21 **Matches:** 1(0) **Sequences:** 1(0)

ZW10 interactor OS=Rattus norvegicus GN=Zwint PE=1 SV=1

Query	Observed	Mr(expt)	Mr(calc)	ppm	Miss	Score	Expect	Rank	Unique	Peptide
1635	638.82	1275.63	1275.61	12.3	1	21	0.068	2	U	-.MADAEKNAVAEK.N

---

174. Q498M5 **Score:** 20 **Matches:** 1(1) **Sequences:** 1(1) **emPAI:** 0.05

Putative E3 ubiquitin-protein ligase SH3RF2 OS=Rattus norvegicus GN=Sh3rf2 PE=2 SV=1

Query	Observed	Mr(expt)	Mr(calc)	ppm	Miss	Score	Expect	Rank	Unique	Peptide
1107	550.84	1099.66	1099.63	20.8	1	20	0.034	4	U	R.ILTLQDNRK.A

---

175. P13383 **Mass:** 77158 **Score:** 20 **Matches:** 1(1) **Sequences:** 1(1) **emPAI:** 0.06

Nucleolin OS=Rattus norvegicus GN=Ncl PE=1 SV=3

Query	Observed	Mr(expt)	Mr(calc)	ppm	Miss	Score	Expect	Rank	Unique	Peptide
466	**450.27**	**898.52**	**898.52**	**-0.66**	**1**	**20**	**0.047**	**1**	**U**	**K.GAVTPAKGAK.N**

Mascot: http://www.matrixscience.com/

Mascot Search Results

```
User                   : proteomics.masas
Email                  : proteomics.masas@cnb.csic.es
Search title           : Susana Bravo_Rodentia_20150603
Database               : _UKBsp_all sprot_20150210 (547599 sequences; 195014757 residues)
Taxonomy               : Rattus (7932 sequences)
Timestamp              : 3 Jun 2015 at 09:22:24 GMT
Enzyme                 : Trypsin/P
Fixed modifications    : Carbamidomethyl (C)
Variable modifications : Oxidation (M)
Mass values            : Monoisotopic
Protein Mass           : Unrestricted
Peptide Mass Tolerance : ± 25 ppm
Fragment Mass Tolerance: ± 0.05 Da
Max Missed Cleavages   : 1
Instrument type        : ESI-QUAD-TOF
Number of queries      : 3402
Protein hits           : P10111 Peptidyl-prolyl cis-trans isomerase A OS=Rattus norvegicus GN=Ppia PE=1 SV=2
                         Q64119 Myosin light polypeptide 6 OS=Rattus norvegicus GN=Myl6 PE=1 SV=3
                         Q9R063 Peroxiredoxin-5, mitochondrial OS=Rattus norvegicus GN=Prdx5 PE=1 SV=1
                         P17074 40S ribosomal protein S19 OS=Rattus norvegicus GN=Rps19 PE=2 SV=3
                         P19804 Nucleoside diphosphate kinase B OS=Rattus norvegicus GN=Nme2 PE=1 SV=1
                         P10888 Cytochrome c oxidase subunit 4 isoform 1, mitochondrial OS=Rattus norvegicus GN=Cox4i1 PE=1 SV=1
                         Q6IFW6 Keratin, type I cytoskeletal 10 OS=Rattus norvegicus GN=Krt10 PE=3 SV=1
                         P04055 Phospholipase A2 OS=Rattus norvegicus GN=Pla2g1b PE=1 SV=1
                         P13832 Myosin regulatory light chain RLC-A OS=Rattus norvegicus GN=Rlc-a PE=2 SV=2
                         Q00715 Histone H2B type 1 OS=Rattus norvegicus PE=1 SV=2
                         P62804 Histone H4 OS=Rattus norvegicus GN=Hist1h4b PE=1 SV=2
                         Q7M0E3 Destrin OS=Rattus norvegicus GN=Dstn PE=1 SV=3
                         Q6IMF3 Keratin, type II cytoskeletal 1 OS=Rattus norvegicus GN=Krt1 PE=2 SV=1
                         P62853 40S ribosomal protein S25 OS=Rattus norvegicus GN=Rps25 PE=2 SV=1
                         Q6P6Q2 Keratin, type II cytoskeletal 5 OS=Rattus norvegicus GN=Krt5 PE=1 SV=1
                         P62832 60S ribosomal protein L23 OS=Rattus norvegicus GN=Rpl23 PE=2 SV=1
                         P02091 Hemoglobin subunit beta-1 OS=Rattus norvegicus GN=Hbb PE=1 SV=3
                         P01946 Hemoglobin subunit alpha-1/2 OS=Rattus norvegicus GN=Hba1 PE=1 SV=3
                         P62902 60S ribosomal protein L31 OS=Rattus norvegicus GN=Rpl31 PE=2 SV=1
                         Q6IFU8 Keratin, type I cytoskeletal 17 OS=Rattus norvegicus GN=Krt17 PE=2 SV=1
                         P13471 40S ribosomal protein S14 OS=Rattus norvegicus GN=Rps14 PE=2 SV=3
                         P60711 Actin, cytoplasmic 1 OS=Rattus norvegicus GN=Actb PE=1 SV=1
                         Q4FZU2 Keratin, type II cytoskeletal 6A OS=Rattus norvegicus GN=Krt6a PE=1 SV=1
                         P62246 40S ribosomal protein S15a OS=Rattus norvegicus GN=Rps15a PE=1 SV=2
                         P07632 Superoxide dismutase [Cu-Zn] OS=Rattus norvegicus GN=Sod1 PE=1 SV=2
                         P0C169 Histone H2A type 1-C OS=Rattus norvegicus PE=1 SV=2
                         P62271 40S ribosomal protein S18 OS=Rattus norvegicus GN=Rps18 PE=1 SV=3
                         P62250 40S ribosomal protein S16 OS=Rattus norvegicus GN=Rps16 PE=1 SV=2
                         P84817 Mitochondrial fission 1 protein OS=Rattus norvegicus GN=Fis1 PE=1 SV=1
                         Q6IG02 Keratin, type II cytoskeletal 2 epidermal OS=Rattus norvegicus GN=Krt2 PE=3 SV=1
                         Q10758 Keratin, type II cytoskeletal 8 OS=Rattus norvegicus GN=Krt8 PE=1 SV=3
                         P02770 Serum albumin OS=Rattus norvegicus GN=Alb PE=1 SV=2
                         P16409 Myosin light chain 3 OS=Rattus norvegicus GN=Myl3 PE=2 SV=2
                         P10719 ATP synthase subunit beta, mitochondrial OS=Rattus norvegicus GN=Atp5b PE=1 SV=2
                         P62161 Calmodulin OS=Rattus norvegicus GN=Calm1 PE=1 SV=2
                         Q3T1J1 Eukaryotic translation initiation factor 5A-1 OS=Rattus norvegicus GN=Eif5a PE=1 SV=3
                         Q6IG03 Keratin, type II cytoskeletal 73 OS=Rattus norvegicus GN=Krt73 PE=1 SV=1
                         Q6LED0 Histone H3.1 OS=Rattus norvegicus PE=1 SV=3
                         Q27W02 Protein mago nashi homolog OS=Rattus norvegicus GN=Magoh PE=2 SV=1
                         P35434 ATP synthase subunit delta, mitochondrial OS=Rattus norvegicus GN=Atp5d PE=1 SV=2
                         Q9EQX9 Ubiquitin-conjugating enzyme E2 N OS=Rattus norvegicus GN=Ube2n PE=1 SV=1
                         P63039 60 kDa heat shock protein, mitochondrial OS=Rattus norvegicus GN=Hspd1 PE=1 SV=1
                         P00173 Cytochrome b5 OS=Rattus norvegicus GN=Cyb5a PE=1 SV=2
                         Q09073 ADP/ATP translocase 2 OS=Rattus norvegicus GN=Slc25a5 PE=1 SV=3
                         P62856 40S ribosomal protein S26 OS=Rattus norvegicus GN=Rps26 PE=3 SV=3
                         Q5XFX0 Transgelin-2 OS=Rattus norvegicus GN=Tagln2 PE=2 SV=1
                         P62890 60S ribosomal protein L30 OS=Rattus norvegicus GN=Rpl30 PE=3 SV=2
                         Q7M767 Ubiquitin-conjugating enzyme E2 variant 2 OS=Rattus norvegicus GN=Ube2v2 PE=1 SV=3
                         Q5BJY9 Keratin, type I cytoskeletal 18 OS=Rattus norvegicus GN=Krt18 PE=1 SV=3
                         P47198 60S ribosomal protein L22 OS=Rattus norvegicus GN=Rpl22 PE=2 SV=2
                         P62268 40S ribosomal protein S23 OS=Rattus norvegicus GN=Rps23 PE=1 SV=3
                         P15865 Histone H1.4 OS=Rattus norvegicus GN=Hist1h1e PE=1 SV=3
                         P62963 Profilin-1 OS=Rattus norvegicus GN=Pfn1 PE=1 SV=2
                         P00406 Cytochrome c oxidase subunit 2 OS=Rattus norvegicus GN=Mtco2 PE=2 SV=2
                         Q4QQV3 Protein FAM162A OS=Rattus norvegicus GN=Fam162a PE=2 SV=1
                         P60868 40S ribosomal protein S20 OS=Rattus norvegicus GN=Rps20 PE=3 SV=1
                         P04644 40S ribosomal protein S17 OS=Rattus norvegicus GN=Rps17 PE=1 SV=3
                         P61354 60S ribosomal protein L27 OS=Rattus norvegicus GN=Rpl27 PE=2 SV=2
                         Q3KRE8 Tubulin beta-2B chain OS=Rattus norvegicus GN=Tubb2b PE=1 SV=1
                         Q6P9T8 Tubulin beta-4B chain OS=Rattus norvegicus GN=Tubb4b PE=1 SV=1
                         Q05962 ADP/ATP translocase 1 OS=Rattus norvegicus GN=Slc25a4 PE=1 SV=3
                         P63324 40S ribosomal protein S12 OS=Rattus norvegicus GN=Rps12 PE=1 SV=2
                         Q4KLF8 Actin-related protein 2/3 complex subunit 5 OS=Rattus norvegicus GN=Arpc5 PE=1 SV=3
                         B0BNA5 Coactosin-like protein OS=Rattus norvegicus GN=Cotl1 PE=1 SV=1
                         P62278 40S ribosomal protein S13 OS=Rattus norvegicus GN=Rps13 PE=1 SV=2
                         P62982 Ubiquitin-40S ribosomal protein S27a OS=Rattus norvegicus GN=Rps27a PE=1 SV=2
                         P11250 60S ribosomal protein L34 OS=Rattus norvegicus GN=Rpl34 PE=1 SV=3
                         B0K025 Oligosaccharyltransferase complex subunit OSTC OS=Rattus norvegicus GN=Ostc PE=2 SV=1
                         Q63495 Advanced glycosylation end product-specific receptor OS=Rattus norvegicus GN=Ager PE=1 SV=1
                         P37397 Calponin-3 OS=Rattus norvegicus GN=Cnn3 PE=1 SV=1
                         P02773 Alpha-fetoprotein OS=Rattus norvegicus GN=Afp PE=2 SV=1
                         P35213 14-3-3 protein beta/alpha OS=Rattus norvegicus GN=Ywhab PE=1 SV=3
                         P18598 Potassium-transporting ATPase subunit beta OS=Rattus norvegicus GN=Atp4b PE=2 SV=3
                         Q8K1Q4 Leucine zipper putative tumor suppressor 3 OS=Rattus norvegicus GN=Lzts3 PE=1 SV=2
                         Q4KLH6 Centrosomal protein of 162 kDa OS=Rattus norvegicus GN=Cep162 PE=1 SV=2
                         P15999 ATP synthase subunit alpha, mitochondrial OS=Rattus norvegicus GN=Atp5a1 PE=1 SV=2
                         P39032 60S ribosomal protein L36 OS=Rattus norvegicus GN=Rpl36 PE=1 SV=2
                         Q29TV8 Gastrokine-2 OS=Rattus norvegicus GN=Gkn2 PE=2 SV=1
                         Q6AYZ1 Tubulin alpha-1C chain OS=Rattus norvegicus GN=Tuba1c PE=1 SV=1
                         P17078 60S ribosomal protein L35 OS=Rattus norvegicus GN=Rpl35 PE=1 SV=3
                         O70454 Protein BUD31 homolog OS=Rattus norvegicus GN=Bud31 PE=2 SV=2
                         P13383 Nucleolin OS=Rattus norvegicus GN=Ncl PE=1 SV=3
                         P00762 Anionic trypsin-1 OS=Rattus norvegicus GN=Prss1 PE=1 SV=1
                         P83883 60S ribosomal protein L36a OS=Rattus norvegicus GN=Rpl36a PE=1 SV=2
                         O88761 26S proteasome non-ATPase regulatory subunit 1 OS=Rattus norvegicus GN=Psmd1 PE=2 SV=1
                         P52632 Signal transducer and activator of transcription 5B OS=Rattus norvegicus GN=Stat5b PE=2 SV=1
```

P08426 Cationic trypsin-3 OS=Rattus norvegicus GN=Try3 PE=2 SV=1
D4AE41 RNA binding motif protein, X-linked-like-1 OS=Rattus norvegicus GN=Rbmxl1 PE=3 SV=1
P26769 Adenylate cyclase type 2 OS=Rattus norvegicus GN=Adcy2 PE=1 SV=1
O88778 Protein bassoon OS=Rattus norvegicus GN=Bsn PE=1 SV=3
P0C5H9 Mesencephalic astrocyte-derived neurotrophic factor OS=Rattus norvegicus GN=Manf PE=1 SV=1
Q5M7V8 Thyroid hormone receptor-associated protein 3 OS=Rattus norvegicus GN=Thrap3 PE=1 SV=1
Q9EPY0 Caspase recruitment domain-containing protein 9 OS=Rattus norvegicus GN=Card9 PE=1 SV=1
P47819 Glial fibrillary acidic protein OS=Rattus norvegicus GN=Gfap PE=1 SV=2
Q9Z137 Galactosylgalactosylxylosylprotein 3-beta-glucuronosyltransferase 2 OS=Rattus norvegicus GN=B3gat2 PE=
P29117 Peptidyl-prolyl cis-trans isomerase F, mitochondrial OS=Rattus norvegicus GN=Ppif PE=1 SV=2
Q9JLA3 UDP-glucose:glycoprotein glucosyltransferase 1 OS=Rattus norvegicus GN=Uggt1 PE=1 SV=2
Q05030 Platelet-derived growth factor receptor beta OS=Rattus norvegicus GN=Pdgfrb PE=2 SV=2
Q9JKS6 Protein piccolo OS=Rattus norvegicus GN=Pclo PE=1 SV=1
P86411 Ral GTPase-activating protein subunit alpha-2 OS=Rattus norvegicus GN=Ralgapa2 PE=1 SV=1
P67779 Prohibitin OS=Rattus norvegicus GN=Phb PE=1 SV=1
P61078 Ubiquitin-conjugating enzyme E2 D3 OS=Rattus norvegicus GN=Ube2d3 PE=2 SV=1
Q9QX74 SH3 and multiple ankyrin repeat domains protein 2 OS=Rattus norvegicus GN=Shank2 PE=1 SV=2
Q6QLM7 Kinesin heavy chain isoform 5A OS=Rattus norvegicus GN=Kif5a PE=1 SV=1
P36372 Antigen peptide transporter 2 OS=Rattus norvegicus GN=Tap2 PE=2 SV=1
P62870 Transcription elongation factor B polypeptide 2 OS=Rattus norvegicus GN=Tceb2 PE=1 SV=1
O54939 Testosterone 17-beta-dehydrogenase 3 OS=Rattus norvegicus GN=Hsd17b3 PE=2 SV=1
Q80T22 Sodium-dependent glucose transporter 1 OS=Rattus norvegicus GN=Naglt1 PE=2 SV=1
Q07066 Peroxisomal membrane protein 2 OS=Rattus norvegicus GN=Pxmp2 PE=1 SV=2
P0C0R5 Phosphoinositide 3-kinase regulatory subunit 4 OS=Rattus norvegicus GN=Pik3r4 PE=3 SV=2
Q6AXQ7 IQ and AAA domain-containing protein 1-like OS=Rattus norvegicus GN=Iqca1p1 PE=2 SV=2
P22791 Hydroxymethylglutaryl-CoA synthase, mitochondrial OS=Rattus norvegicus GN=Hmgcs2 PE=2 SV=1

Select Summary Report

Format As [Select Summary (protein hits)] Help

Significance threshold p< [0.05] Max. number of hits [AUTO]

Standard scoring ◉ MudPIT scoring ○ Ions score or expect cut-off [20] Show sub-sets [0]

Show pop-ups ◉ Suppress pop-ups ○ Require bold red ☐

Preferred taxonomy [All entries]

Re-Search ◉ All queries ○ Unassigned ○ Below homology threshold ○ Below identity threshold

1. P10111 **Mass:** 18091 **Score:** 658 **Matches:** 12(12) **Sequences:** 9(9) **emPAI:** 8.57

Peptidyl-prolyl cis-trans isomerase A OS=Rattus norvegicus GN=Ppia PE=1 SV=2

Query	Observed	Mr(expt)	Mr(calc)	ppm	Miss	Score	Expect	Rank	Unique	Peptide
821	503.76	1005.50	1005.48	24.1	0	32	0.0068	1	U	K.ITISDCGQL.-
1235	564.77	1127.53	1127.53	-3.41	0	59	6e-006	1	U	R.VCFELFADK.V
1260	567.79	1133.57	1133.58	-1.61	1	68	1.4e-006	1	U	K.KITISDCGQL.-
1323	577.79	1153.56	1153.57	-2.65	0	53	3e-005	1	U	K.FEDENFILK.H
1717	642.28	1282.55	1282.55	-2.55	0	59	3e-006	1	U	K.EGMSIVEAMER.F
2133	484.92	1451.75	1451.75	-1.57	1	74	2.2e-007	1	U	R.VCFELFADVPK.T
2430	799.87	1597.73	1597.74	-4.22	0	(65)	1.8e-006	1	U	R.IIPGFMCQGGDFTR.H
2463	807.87	1613.73	1613.73	-2.40	0	70	4.3e-007	1	U	R.IIPGFMCQGGDFTR.H
2464	538.92	1613.74	1613.73	5.16	0	(31)	0.0038	1	U	R.IIPGFMCQGGDFTR.H
2773	916.46	1830.90	1830.90	-1.94	1	102	3.7e-010	1	U	K.SIYGEKFEDENFILK.H
2774	611.31	1830.90	1830.90	-1.77	1	(38)	0.00093	1	U	K.SIYGEKFEDENFILK.H
3327	936.44	2806.31	2806.32	-2.81	0	141	2.7e-014	1	U	K.HTGPGILSMANAGPNTNGSQFFICTAK.T

2. Q64119 **Mass:** 17135 **Score:** 548 **Matches:** 10(10) **Sequences:** 8(8) **emPAI:** 7.57

Myosin light polypeptide 6 OS=Rattus norvegicus GN=Myl6 PE=1 SV=3

Query	Observed	Mr(expt)	Mr(calc)	ppm	Miss	Score	Expect	Rank	Unique	Peptide
759	498.30	994.58	994.58	-3.12	0	55	8.7e-006	1	U	R.HVLVTLGEK.M
878	513.26	1024.50	1024.50	-0.92	0	48	9.6e-005	1		K.EAFQLFDR.T
1869	671.31	1340.61	1340.62	-5.79	0	(75)	1.4e-007	1	U	K.ILYSQCGDVMR.A
1905	677.87	1353.72	1353.73	-3.06	0	70	5.1e-007	1	U	R.ALGQNPTNAEVLK.V
1911	453.21	1356.61	1356.62	-4.03	0	(30)	0.0037	1	U	K.ILYSQCGDVMR.A
1912	679.31	1356.62	1356.62	-0.90	0	78	5.6e-008	1	U	K.ILYSQCGDVMR.A
2337	772.85	1543.68	1543.68	-1.10	0	78	5.3e-008	1	U	K.DQGTYEDYVEGLR.V
2627	580.28	1737.83	1737.84	-3.29	1	83	3.2e-008	1	U	R.VFDKEGNGTVMGAEIR.H
2701	596.28	1785.81	1785.82	-2.49	1	84	1.3e-008	1	U	K.NKDQGTYEDYVEGLR.V
2848	952.50	1902.98	1902.99	-5.03	0	51	3.9e-005	1	U	K.VLDFEHFLPMLQTVAK.N

3. Q9R063 **Mass:** 22507 **Score:** 515 **Matches:** 9(9) **Sequences:** 7(7) **emPAI:** 3.29

Peroxiredoxin-5, mitochondrial OS=Rattus norvegicus GN=Prdx5 PE=1 SV=1

Query	Observed	Mr(expt)	Mr(calc)	ppm	Miss	Score	Expect	Rank	Unique	Peptide
246	420.22	838.42	838.43	-2.20	0	(44)	0.00027	1	U	R.FSMVIDK.G
355	428.22	854.42	854.42	1.75	0	56	3.8e-005	1	U	R.FSMVIDK.G
579	467.27	932.53	932.53	-0.69	0	49	6e-005	1	U	K.VNLAELFK.D
996	531.32	1060.62	1060.63	-4.57	1	66	6.2e-007	1	U	K.KVNLAELFK.D
1811	658.86	1315.71	1315.71	-1.81	0	87	1.3e-008	1	U	K.VQLLADPTGAFGK.E 1810
2172	489.94	1466.79	1466.79	-0.78	0	95	1.4e-009	1	U	K.THLPGFVEQAGALK.A
2418	797.41	1592.80	1592.80	-3.00	0	73	7e-007	1	U	K.GVLFGVPGAFTPGCSK.T
2831	944.49	1886.96	1886.96	-2.60	1	88	9.7e-009	1	U	K.VGDTIPSVEVFEGEPGKK.V

4. P17074 **Mass:** 16076 **Score:** 383 **Matches:** 8(8) **Sequences:** 8(8) **emPAI:** 6.58

40S ribosomal protein S19 OS=Rattus norvegicus GN=Rps19 PE=2 SV=3

Query	Observed	Mr(expt)	Mr(calc)	ppm	Miss	Score	Expect	Rank	Unique	Peptide
38	367.23	732.45	732.45	-1.80	0	43	0.0001	1	U	R.ALAAFLK.K
597	471.77	941.53	941.53	-2.37	0	73	3e-007	1	U	R.IAGQVAAANK.K
689	485.80	969.59	969.59	-0.34	0	60	3.2e-006	1	U	R.VLQALEGLK.M
1042	536.79	1071.57	1071.56	7.59	0	39	0.0011	1	U	K.VPEWVDTVK.L
1230	563.85	1125.69	1125.69	-1.33	1	48	3e-005	1	U	R.RVLQALEGLK.M

1258	567.78	1133.55	1133.55	-0.66	0	47	0.00016	1	U	K.DVNQQEFVR.A
1802	657.37	1312.73	1312.74	-4.16	1	38	0.00077	1	U	K.LKVPEWVDTVK.L
2583	852.39	1702.77	1702.76	6.69	0	37	0.00079	1	U	K.ELAPYDENWFYTR.A

5. P19804 **Mass:** 17386 **Score:** 383 **Matches:** 8(8) **Sequences:** 7(7) **emPAI:** 4.16
Nucleoside diphosphate kinase B OS=Rattus norvegicus GN=Nme2 PE=1 SV=1

Query	Observed	Mr(expt)	Mr(calc)	ppm	Miss	Score	Expect	Rank	Unique	Peptide
221	414.76	827.51	827.51	0.59	0	44	0.0002	1	U	R.GLVGEIIK.R
728	492.80	983.59	983.61	-19.17	1	23	0.017	1	U	R.GLVGEIIKR.F
965	526.25	1050.49	1050.49	-2.74	0	49	7.2e-005	1	U	R.GDFCIQVGR.N
1382	588.32	1174.63	1174.65	-13.69	1	36	0.0018	1	U	K.DRPFFPGLVK.Y
1880	672.87	1343.73	1343.76	-20.14	1	(44)	0.00026	1	U	R.TFIAIKPDGVQR.G
1882	448.92	1343.74	1343.76	-11.58	1	56	1.4e-005	1	U	R.TFIAIKPDGVQR.G
2213	495.91	1484.71	1484.71	-2.58	0	110	4.8e-011	1	U	R.NIIHGSDSVESAEK.E
2734	601.31	1800.91	1800.90	0.69	1	65	2.2e-006	1	U	R.VMLGETNPADSKPGTIR.G

6. P10888 **Mass:** 19559 **Score:** 368 **Matches:** 8(8) **Sequences:** 8(8) **emPAI:** 4.34
Cytochrome c oxidase subunit 4 isoform 1, mitochondrial OS=Rattus norvegicus GN=Cox4i1 PE=1 SV=1

Query	Observed	Mr(expt)	Mr(calc)	ppm	Miss	Score	Expect	Rank	Unique	Peptide
27	363.17	724.32	724.32	-0.77	0	25	0.0098	1	U	K.WDYNK.N
544	461.22	920.43	920.44	-0.91	0	25	0.027	1	U	K.ADWSSLSR.D
989	530.79	1059.57	1059.57	-2.91	0	50	9.6e-005	1	U	K.VNPIQGFSAK.W
1709	428.20	1281.58	1281.58	-1.52	1	37	0.00085	1	U	K.WDYNKNEWK.K
2043	707.83	1413.64	1413.64	-0.69	0	41	0.00027	1	U	K.SEDYALPSYVDR.R
2179	737.34	1472.66	1472.66	-2.49	0	79	3.8e-008	1	U	R.IQFNESFAEMNK.G
2355	517.93	1550.75	1550.75	1.22	0	65	2e-006	1	U	K.SYVYGPIPHTFDR.D
2382	524.25	1569.74	1569.74	-0.43	1	47	0.00011	1	U	K.SEDYALPSYVDRR.D

7. Q6IFW6 **Mass:** 56699 **Score:** 357 **Matches:** 6(6) **Sequences:** 6(6) **emPAI:** 0.55
Keratin, type I cytoskeletal 10 OS=Rattus norvegicus GN=Krt10 PE=3 SV=1

Query	Observed	Mr(expt)	Mr(calc)	ppm	Miss	Score	Expect	Rank	Unique	Peptide
166	404.21	806.40	806.39	8.69	0	22	0.047	1		R.LAADDFR.L
1164	555.25	1108.49	1108.48	7.27	0	38	0.00063	1	U	K.DAEAWFNEK.S
1362	583.29	1164.57	1164.58	-5.44	0	65	2.3e-006	1	U	R.LENEIQTYR.S
1478	601.32	1200.62	1200.61	5.55	0	93	3.8e-009	1	U	R.QSVEADINGLR.R
1914	453.24	1356.71	1356.71	-0.73	1	55	2.2e-005	1	U	R.QSVEADINGLRR.V
1992	695.84	1389.67	1389.67	-3.04	0	84	3.3e-008	1	U	K.QSLEASLAETEGR.Y

8. P04055 **Mass:** 17211 **Score:** 355 **Matches:** 6(6) **Sequences:** 6(6) **emPAI:** 3.15
Phospholipase A2 OS=Rattus norvegicus GN=Pla2g1b PE=1 SV=1

Query	Observed	Mr(expt)	Mr(calc)	ppm	Miss	Score	Expect	Rank	Unique	Peptide
165	403.72	805.42	805.42	-0.99	0	30	0.007	1	U	R.AVWQFR.N
1181	558.28	1114.55	1114.54	1.25	0	59	7.7e-006	1	U	K.CTIPGSDPLR.E
1601	620.77	1239.52	1239.52	-3.64	0	75	4.6e-008	1	U	K.CSGNVITCSDK.N
2582	852.31	1702.61	1702.61	0.25	0	62	6.6e-007	1	U	K.NNDCESFICNCDR.Q
2626	869.92	1737.82	1737.82	-2.28	0	86	1.3e-008	1	U	K.FLIDNPYTNTYSYK.C
3270	861.36	2581.05	2581.05	-0.74	0	43	4.8e-005	1	U	R.EYNNYGCYCGLGGSGTPVDDLDR.C

9. P13832 **Mass:** 19940 **Score:** 348 **Matches:** 9(8) **Sequences:** 7(6) **emPAI:** 4.15
Myosin regulatory light chain RLC-A OS=Rattus norvegicus GN=Rlc-a PE=2 SV=2

Query	Observed	Mr(expt)	Mr(calc)	ppm	Miss	Score	Expect	Rank	Unique	Peptide
904	518.26	1034.50	1034.51	-3.26	0	(53)	4e-005	1	U	R.ELLTTMGDR.F
966	526.26	1050.50	1050.50	-1.30	0	61	5e-006	1	U	R.ELLTTMGDR.F
1557	614.81	1227.61	1227.61	-0.68	0	21	0.063	1	U	K.LNGTDPEDVIR.N
1582	619.28	1236.55	1236.56	-6.59	0	(26)	0.01	1	U	K.EAFNMIDQNR.D
1642	627.28	1252.55	1252.55	1.23	0	57	4.5e-006	1	U	K.EAFNMIDQNR.D
1656	630.80	1259.59	1259.59	-4.55	0	43	0.00024	1	U	K.GNFNYIEFTR.I
2046	472.55	1414.62	1414.63	-0.34	0	26	0.0092	1	U	R.FTDEEVDELYR.E
3010	514.23	2052.91	2052.91	-2.17	1	24	0.0097	1	U	R.DGFIDKEDLHDMLASMGK.N
3035	1053.99	2105.96	2105.97	-3.82	0	116	1e-011	1	U	R.ATSNVFAMFDQSQIQEFK.E

10. Q00715 **Mass:** 13982 **Score:** 284 **Matches:** 6(6) **Sequences:** 6(6) **emPAI:** 4.71
Histone H2B type 1 OS=Rattus norvegicus PE=1 SV=2

Query	Observed	Mr(expt)	Mr(calc)	ppm	Miss	Score	Expect	Rank	Unique	Peptide
185	408.73	815.45	815.45	-2.33	0	34	0.0048	1	U	R.EIQTAVR.L
652	477.30	952.60	952.60	-0.59	0	33	0.0006	1	U	R.LLLPGELAK.H
1266	569.28	1136.54	1136.54	-0.06	0	43	0.00073	1	U	K.ESYSVYVYK.V
1368	390.20	1167.59	1167.59	-0.88	0	74	2.6e-007	1	U	K.QVHPDTGISSK.A
1666	422.55	1264.63	1264.63	-0.80	1	21	0.057	1	U	R.KESYSVYVYK.V
2682	888.41	1774.81	1774.80	2.90	0	79	5.4e-008	1	U	K.AMGIMNSFVNDIFER.I

11. P62804 **Mass:** 11360 **Score:** 264 **Matches:** 6(6) **Sequences:** 6(6) **emPAI:** 7.51
Histone H4 OS=Rattus norvegicus GN=Hist1h4b PE=1 SV=2

Query	Observed	Mr(expt)	Mr(calc)	ppm	Miss	Score	Expect	Rank	Unique	Peptide
440	444.74	887.47	887.47	-5.15	0	31	0.014	1	U	R.DNIQGITK.P
744	495.29	988.57	988.57	-1.45	0	37	0.0012	1	U	K.VFLENVIR.D
1257	378.85	1133.54	1133.54	1.79	0	52	4.2e-005	1	U	R.DAVTYTEHAK.R
1413	590.81	1179.61	1179.61	-5.54	0	38	0.001	1	U	R.ISGLIYEETR.G
1833	442.59	1324.74	1324.75	-4.64	1	42	0.00018	1	U	R.DNIQGITKPAIR.R
1835	663.85	1325.69	1325.69	-2.83	0	64	2.2e-006	1	U	K.TVTAMDVVYALK.R

12. Q7M0E3 **Mass:** 18807 **Score:** 260 **Matches:** 5(5) **Sequences:** 5(5) **emPAI:** 1.97
Destrin OS=Rattus norvegicus GN=Dstn PE=1 SV=3

Query	Observed	Mr(expt)	Mr(calc)	ppm	Miss	Score	Expect	Rank	Unique	Peptide
577	467.24	932.46	932.46	-3.12	0	38	0.0017	1	U	K.CIVVEEGK.E
1220	562.29	1122.57	1122.57	-5.04	0	53	0.00011	1	U	K.AVIFCLSADK.K
1636	626.34	1250.67	1250.67	-1.53	1	54	2.7e-005	1	U	K.AVIFCLSADKK.C
1782	654.31	1306.61	1306.61	1.27	0	60	5.5e-006	1	U	R.YALYDASFETK.E
2331	514.90	1541.68	1541.69	-1.67	0	57	6.8e-006	1	U	K.HEYQANGPEDLNR.T

13. Q6IMF3 **Mass:** 65190 **Score:** 255 **Matches:** 4(4) **Sequences:** 4(4) **emPAI:** 0.29
Keratin, type II cytoskeletal 1 OS=Rattus norvegicus GN=Krt1 PE=2 SV=1

Query	Observed	Mr(expt)	Mr(calc)	ppm	Miss	Score	Expect	Rank	Unique	Peptide
514	455.71	909.41	909.41	-2.30	0	29	0.0058	1		K.YEDEINK.R
2003	465.25	1392.72	1392.72	-5.12	1	80	8.5e-008	1	U	R.TNAENEFVTIKK.D
2184	738.38	1474.74	1474.74	-1.80	0	74	2.7e-007	1	U	K.WELLQQVDTSTR.T
2185	738.39	1474.77	1474.78	-4.24	0	73	3.1e-007	1		R.FLEQQNQVLQTK.W

14. P62853 **Mass:** 13791 **Score:** 254 **Matches:** 5(5) **Sequences:** 5(5) **emPAI:** 3.37
40S ribosomal protein S25 OS=Rattus norvegicus GN=Rps25 PE=2 SV=1

Query	Observed	Mr(expt)	Mr(calc)	ppm	Miss	Score	Expect	Rank	Unique	Peptide
346	425.74	849.47	849.47	-3.55	0	48	8.2e-005	1	U	R.AQVIYTR.N
565	465.69	929.37	929.37	-3.64	0	44	4.2e-005	1	U	K.GGDAPAAGEDA.-
695	486.79	971.56	971.57	-0.27	0	68	9.9e-007	1	U	R.AALQELLSK.G
1082	542.82	1083.63	1083.63	-2.80	0	60	5.1e-006	1	U	K.LITPAVVSER.L
1816	440.25	1317.73	1317.73	-0.58	1	35	0.0015	1	U	R.DKLNNLVLFDK.A

15. Q6P6Q2 **Mass:** 61959 **Score:** 246 **Matches:** 6(5) **Sequences:** 6(5) **emPAI:** 0.40
Keratin, type II cytoskeletal 5 OS=Rattus norvegicus GN=Krt5 PE=1 SV=1

Query	Observed	Mr(expt)	Mr(calc)	ppm	Miss	Score	Expect	Rank	Unique	Peptide
35	366.70	731.38	731.38	-2.08	0	22	0.12	1		R.LDSELR.N
514	455.71	909.41	909.41	-2.30	0	29	0.0058	1		K.YEDEINK.R
792	503.25	1004.48	1004.46	16.9	0	25	0.025	1		K.LLEGEECR.L
1456	597.79	1193.56	1193.57	-4.02	0	37	0.0013	1	U	K.YEELQQTAGR.H
1481	602.32	1202.62	1202.63	-4.26	0	51	5.9e-005	1	U	K.WTLLQEQGTK.T
1765	651.86	1301.70	1301.71	-2.99	0	82	4e-008	1		R.SLDLDSIIAEVK.A

16. P62832 **Mass:** 14970 **Score:** 240 **Matches:** 4(4) **Sequences:** 4(4) **emPAI:** 1.96
60S ribosomal protein L23 OS=Rattus norvegicus GN=Rpl23 PE=2 SV=1

Query	Observed	Mr(expt)	Mr(calc)	ppm	Miss	Score	Expect	Rank	Unique	Peptide
158	402.22	802.42	802.42	2.13	0	25	0.041	1	U	R.IASNAGSIA.-
640	475.29	948.57	948.56	1.16	0	34	0.0015	1	U	K.NLYIISVK.G
2225	746.38	1490.74	1490.75	-1.82	0	78	1.1e-007	1	U	R.LPAAGVGDMVMATVK.K
2926	985.52	1969.02	1969.03	-3.40	0	103	2.4e-010	1	U	R.ISLGLPVGAVINCADNTGAK.N

17. P02091 **Mass:** 16083 **Score:** 226 **Matches:** 4(4) **Sequences:** 4(4) **emPAI:** 1.75
Hemoglobin subunit beta-1 OS=Rattus norvegicus GN=Hbb PE=1 SV=3

Query	Observed	Mr(expt)	Mr(calc)	ppm	Miss	Score	Expect	Rank	Unique	Peptide
1104	545.80	1089.58	1089.58	-4.09	0	41	0.00072	1	U	K.VINAFNDGLK.H
1219	374.89	1121.66	1121.66	-0.09	0	82	1.4e-008	1	U	K.VVAGVASALAHK.Y
1695	425.58	1273.72	1273.72	-1.79	0	44	0.00017	1	U	R.LLVVYPWTQR.Y
1757	649.82	1297.63	1297.63	-0.13	0	58	8.8e-006	1	U	K.VNPDDVGGEALGR.L

18. P01946 **Mass:** 15490 **Score:** 212 **Matches:** 4(4) **Sequences:** 4(4) **emPAI:** 1.85
Hemoglobin subunit alpha-1/2 OS=Rattus norvegicus GN=Hba1 PE=1 SV=3

Query	Observed	Mr(expt)	Mr(calc)	ppm	Miss	Score	Expect	Rank	Unique	Peptide
191	409.72	817.43	817.43	-0.76	0	37	0.0022	1	U	R.VDPVNFK.F
1096	544.31	1086.61	1086.62	-10.17	1	38	0.0015	1	U	K.LRVDPVNFK.F
1641	626.86	1251.70	1251.71	-2.34	0	79	4.2e-008	1	U	K.FLASVSTVLTSK.Y
2620	868.43	1734.85	1734.86	-4.61	0	58	1e-005	1	U	K.TYFSHIDVSPGSAQVK.A

19. P62902 **Mass:** 14454 **Score:** 204 **Matches:** 4(4) **Sequences:** 4(4) **emPAI:** 2.09
60S ribosomal protein L31 OS=Rattus norvegicus GN=Rpl31 PE=2 SV=1

Query	Observed	Mr(expt)	Mr(calc)	ppm	Miss	Score	Expect	Rank	Unique	Peptide
540	460.71	919.40	919.41	-3.45	0	43	0.00021	1	U	K.EMGTPDVR.I
738	494.77	987.53	987.53	-2.50	0	44	0.00039	1	U	R.SAINEVVTR.E
1295	573.28	1144.54	1144.54	0.07	0	45	0.00016	1	U	K.NLQTVNVDEN.-
2501	822.96	1643.91	1643.92	-5.06	0	72	3e-007	1	U	K.LYTLVTYVPVTTFK.N

20. Q6IFU8 **Mass:** 48378 **Score:** 184 **Matches:** 5(5) **Sequences:** 5(5) **emPAI:** 0.53
Keratin, type I cytoskeletal 17 OS=Rattus norvegicus GN=Krt17 PE=2 SV=1

Query	Observed	Mr(expt)	Mr(calc)	ppm	Miss	Score	Expect	Rank	Unique	Peptide
166	404.21	806.40	806.39	8.69	0	22	0.047	1		R.LAADDFR.T
171	405.22	808.43	808.43	0.20	0	39	0.0006	1		R.LASYLDK.V
891	515.29	1028.57	1028.59	-18.13	0	42	0.00052	1	U	R.VLDELTLAR.A
1021	532.81	1063.60	1063.60	-5.42	1	35	0.0014	1	U	R.LASYLDKVR.A
1541	408.23	1221.65	1221.64	14.9	1	46	0.00015	1	U	R.TKFETEQALR.M

Proteins matching the same set of peptides:
Q63279 **Mass:** 44609 **Score:** 184 **Matches:** 5(5) **Sequences:** 5(5)
Keratin, type I cytoskeletal 19 OS=Rattus norvegicus GN=Krt19 PE=1 SV=2

21. P13471 **Mass:** 16420 **Score:** 178 **Matches:** 3(3) **Sequences:** 3(3) **emPAI:** 1.11
40S ribosomal protein S14 OS=Rattus norvegicus GN=Rps14 PE=2 SV=3

Query	Observed	Mr(expt)	Mr(calc)	ppm	Miss	Score	Expect	Rank	Unique	Peptide
975	527.78	1053.55	1053.56	-3.11	0	71	5e-007	1	U	K.TPGPGAQSALR.A
1118	547.83	1093.64	1093.65	-6.39	0	40	0.00016	1	U	K.ELGITALHIK.L
2087	715.36	1428.70	1428.71	-5.16	0	67	1.2e-006	1	U	R.IEDVTPIPSDSTR.R

22. P60711 **Mass:** 42052 **Score:** 178 **Matches:** 5(5) **Sequences:** 5(5) **emPAI:** 0.63
Actin, cytoplasmic 1 OS=Rattus norvegicus GN=Actb PE=1 SV=1

Query	Observed	Mr(expt)	Mr(calc)	ppm	Miss	Score	Expect	Rank	Unique	Peptide
109	389.18	776.35	776.35	-0.27	0	41	0.00046	1	U	K.CDVDIR.K
139	398.24	794.47	794.47	0.62	0	42	0.00021	1	U	K.IIAPPER.K
609	473.28	944.54	944.54	-1.89	0	51	4.8e-005	1	U	R.AVFPSIVGR.P
1251	566.77	1131.52	1131.52	-1.82	0	21	0.035	1	U	R.GYSFTTTAER.E
1469	599.76	1197.51	1197.51	-3.76	0	23	0.0088	1	U	K.DSYVGDEAQSK.R

Proteins matching the same set of peptides:
P63259 **Mass:** 42108 **Score:** 178 **Matches:** 5(5) **Sequences:** 5(5)
Actin, cytoplasmic 2 OS=Rattus norvegicus GN=Actg1 PE=1 SV=1

23. Q4FZU2 **Mass:** 59555 **Score:** 177 **Matches:** 3(2) **Sequences:** 3(2) **emPAI:** 0.15
Keratin, type II cytoskeletal 6A OS=Rattus norvegicus GN=Krt6a PE=1 SV=1

Query	Observed	Mr(expt)	Mr(calc)	ppm	Miss	Score	Expect	Rank	Unique	Peptide
35	366.70	731.38	731.38	-2.08	0	22	0.12	1		R.LDSELR.N
1398	590.30	1178.59	1178.59	-1.18	0	72	4.7e-007	1	U	K.YEELQITAGR.H
1765	651.86	1301.70	1301.71	-2.99	0	82	4e-008	1		R.SLDLDSIIAEVK.A

24. P62246 **Mass:** 14944 **Score:** 176 **Matches:** 4(4) **Sequences:** 4(4) **emPAI:** 1.96
40S ribosomal protein S15a OS=Rattus norvegicus GN=Rps15a PE=1 SV=2

Query	Observed	Mr(expt)	Mr(calc)	ppm	Miss	Score	Expect	Rank	Unique	Peptide
63	375.21	748.41	748.41	0.70	0	33	0.0038	1	U	R.FDVQLK.D
131	394.71	787.40	787.40	-0.65	0	41	0.00084	1	U	K.CGVISPR.F
400	436.27	870.53	870.53	2.83	0	46	0.00011	1	U	K.IVVNLTGR.L
747	495.77	989.52	989.52	-5.05	0	55	3.2e-005	1	U	R.MNVLADALK.S

25. P07632 **Mass:** 16073 **Score:** 174 **Matches:** 4(4) **Sequences:** 4(4) **emPAI:** 1.75
Superoxide dismutase [Cu-Zn] OS=Rattus norvegicus GN=Sod1 PE=1 SV=2

Query	Observed	Mr(expt)	Mr(calc)	ppm	Miss	Score	Expect	Rank	Unique	Peptide
774	501.28	1000.54	1000.54	6.51	0	28	0.017	1	U	R.LACGVIGIAQ.-
1269	379.87	1136.59	1136.59	-2.81	0	41	0.00067	1	U	R.HVGDLGNVAAGK.D
1377	587.79	1173.56	1173.56	-1.23	0	62	3.5e-006	1	U	K.DGVANVSIEDR.V
2264	504.26	1509.76	1509.76	-1.40	0	43	0.00033	1	U	K.GDGPVQGVIHFEQK.A

26. P0C169 **Mass:** 14097 **Score:** 174 **Matches:** 3(3) **Sequences:** 3(3) **emPAI:** 1.37
Histone H2A type 1-C OS=Rattus norvegicus PE=1 SV=2

Query	Observed	Mr(expt)	Mr(calc)	ppm	Miss	Score	Expect	Rank	Unique	Peptide
347	425.76	849.50	849.52	-21.33	0	34	0.0012	1	U	R.HLQLAIR.N
605	472.77	943.52	943.52	-0.77	0	67	2.2e-006	1	U	R.AGLQFPVGR.V
2877	966.08	1930.15	1930.16	-5.42	0	73	4.6e-008	1	U	R.VTIAQGGVLPNIQAVLLPK.K

Proteins matching the same set of peptides:
P0C170 **Mass:** 14111 **Score:** 174 **Matches:** 3(3) **Sequences:** 3(3)
Histone H2A type 1-E OS=Rattus norvegicus PE=1 SV=2
P0CC09 **Mass:** 14087 **Score:** 174 **Matches:** 3(3) **Sequences:** 3(3)
Histone H2A type 2-A OS=Rattus norvegicus GN=Hist2h2aa3 PE=1 SV=1
Q4FZT6 **Mass:** 14113 **Score:** 174 **Matches:** 3(3) **Sequences:** 3(3)
Histone H2A type 3 OS=Rattus norvegicus PE=2 SV=3
A9UMV8 **Mass:** 14037 **Score:** 174 **Matches:** 3(3) **Sequences:** 3(3)
Histone H2A.J OS=Rattus norvegicus GN=H2afj PE=2 SV=1
Q00728 **Mass:** 14275 **Score:** 174 **Matches:** 3(3) **Sequences:** 3(3)
Histone H2A type 4 OS=Rattus norvegicus PE=2 SV=2
P02262 **Mass:** 14069 **Score:** 174 **Matches:** 3(3) **Sequences:** 3(3)
Histone H2A type 1 OS=Rattus norvegicus PE=1 SV=2
Q64598 **Mass:** 14167 **Score:** 174 **Matches:** 3(3) **Sequences:** 3(3)
Histone H2A type 1-F OS=Rattus norvegicus PE=3 SV=3

27. P62271 **Mass:** 17708 **Score:** 171 **Matches:** 6(6) **Sequences:** 5(5) **emPAI:** 2.16
40S ribosomal protein S18 OS=Rattus norvegicus GN=Rps18 PE=1 SV=3

Query	Observed	Mr(expt)	Mr(calc)	ppm	Miss	Score	Expect	Rank	Unique	Peptide
105	388.21	774.41	774.41	-8.03	0	34	0.0054	1	U	K.ADIDLTK.R
493	452.26	902.50	902.51	-6.11	1	34	0.0035	1	U	R.KADIDLTK.R
1094	544.29	1086.57	1086.59	-15.30	0	36	0.0021	1	U	R.VITIMQNPR.Q
1625	624.29	1246.57	1246.57	1.96	0	26	0.011	1	U	R.AGELTEDEVER.V
1825	661.35	1320.68	1320.67	9.87	0	40	0.00061	1	U	K.YSQVLANGLDNK.L 1823

28. P62250 **Mass:** 16549 **Score:** 160 **Matches:** 3(3) **Sequences:** 3(3) **emPAI:** 1.10
40S ribosomal protein S16 OS=Rattus norvegicus GN=Rps16 PE=1 SV=2

Query	Observed	Mr(expt)	Mr(calc)	ppm	Miss	Score	Expect	Rank	Unique	Peptide
110	389.22	776.42	776.42	2.55	0	34	0.0066	1	U	R.FAGVDIR.V
1433	594.33	1186.64	1186.65	-5.80	0	82	4.5e-008	1	U	K.GPLQSVQVFGR.K
2025	703.39	1404.76	1404.76	-3.24	1	43	0.00052	1	U	K.EIKDILIQYDR.T

29. P84817 **Mass:** 17041 **Score:** 159 **Matches:** 3(3) **Sequences:** 3(3) **emPAI:** 1.05
Mitochondrial fission 1 protein OS=Rattus norvegicus GN=Fis1 PE=1 SV=1

Query	Observed	Mr(expt)	Mr(calc)	ppm	Miss	Score	Expect	Rank	Unique	Peptide
1543	612.38	1222.76	1222.75	1.44	0	37	0.00019	1	U	R.GIVLLEELLPK.G
1936	456.23	1365.68	1365.69	-4.62	1	66	2.1e-006	1	U	R.KFQSEQAAGSVSK.S
2111	720.87	1439.73	1439.74	-2.79	0	56	1.6e-005	1	U	R.GLLQTEPQNNQAK.E

30. Q6IG02 **Mass:** 69484 **Score:** 149 **Matches:** 4(4) **Sequences:** 4(4) **emPAI:** 0.27
Keratin, type II cytoskeletal 2 epidermal OS=Rattus norvegicus GN=Krt2 PE=3 SV=1

Query	Observed	Mr(expt)	Mr(calc)	ppm	Miss	Score	Expect	Rank	Unique	Peptide
514	455.71	909.41	909.41	-2.30	0	29	0.0058	1		K.YEDEINK.R
792	503.25	1004.48	1004.46	16.9	0	25	0.025	1		K.LLEGEECR.M
1325	578.27	1154.53	1154.53	1.79	0	41	0.00091	1	U	R.DYQELMNVK.L
1648	627.81	1253.60	1253.60	-2.13	0	54	2.2e-005	1	U	R.GFSSGSAVVSGGSR.R

31. Q10758 **Mass:** 53985 **Score:** 142 **Matches:** 4(3) **Sequences:** 4(3) **emPAI:** 0.26
Keratin, type II cytoskeletal 8 OS=Rattus norvegicus GN=Krt8 PE=1 SV=3

Query	Observed	Mr(expt)	Mr(calc)	ppm	Miss	Score	Expect	Rank	Unique	Peptide
514	455.71	909.41	909.41	-2.30	0	29	0.0058	1		K.YEDEINK.R
769	500.79	999.56	999.56	-3.00	0	52	5.4e-005	1	U	R.LQAEIDALK.G
847	508.27	1014.52	1014.53	-11.53	0	21	0.065	1	U	R.QLEALGQEK.L
987	354.19	1059.55	1059.56	-4.39	1	40	0.00096	1	U	R.KLLEGEESR.L

32. P02770 **Mass:** 70682 **Score:** 141 **Matches:** 3(3) **Sequences:** 3(3) **emPAI:** 0.19
Serum albumin OS=Rattus norvegicus GN=Alb PE=1 SV=2

Query	Observed	Mr(expt)	Mr(calc)	ppm	Miss	Score	Expect	Rank	Unique	Peptide
475	449.74	897.47	897.47	-0.05	0	35	0.0021	1	U	R.LCVLHEK.T
1303	575.31	1148.60	1148.61	-6.00	0	32	0.0047	1	U	K.LVQEVTDFAK.T
2649	583.89	1748.65	1748.66	-2.29	0	73	4.6e-008	1	U	K.ECCHGDLLECADDR.A

33. P16409 **Mass:** 22256 **Score:** 131 **Matches:** 2(2) **Sequences:** 2(2) **emPAI:** 0.44
Myosin light chain 3 OS=Rattus norvegicus GN=Myl3 PE=2 SV=2

Query	Observed	Mr(expt)	Mr(calc)	ppm	Miss	Score	Expect	Rank	Unique	Peptide
878	513.26	1024.50	1024.50	-0.92	0	48	9.6e-005	1		K.EAFQLFDR.T
2627	580.28	1737.83	1737.84	-3.29	1	83	3.2e-008	1	U	R.VFDKEGNGTVMGAELR.H

34. P10719 **Mass:** 56318 **Score:** 130 **Matches:** 4(4) **Sequences:** 4(4) **emPAI:** 0.34
ATP synthase subunit beta, mitochondrial OS=Rattus norvegicus GN=Atp5b PE=1 SV=2

Query	Observed	Mr(expt)	Mr(calc)	ppm	Miss	Score	Expect	Rank	Unique	Peptide
117	390.22	778.42	778.42	-5.01	0	41	0.00073	1	U	K.ILQDYK.S
1097	544.82	1087.62	1087.63	-2.98	0	23	0.024	1	U	K.VVDLLAPYAK.G
2100	718.38	1434.75	1434.75	4.88	0	35	0.002	1	U	R.FTQAGSEVSALLGR.I
2950	994.52	1987.02	1987.03	-0.67	0	30	0.0077	1	U	R.AIAELGIYPAVDPLDSTSR.I

35. P62161 **Mass:** 16827 **Score:** 122 **Matches:** 2(2) **Sequences:** 2(2) **emPAI:** 0.62
Calmodulin OS=Rattus norvegicus GN=Calm1 PE=1 SV=2

Query	Observed	Mr(expt)	Mr(calc)	ppm	Miss	Score	Expect	Rank	Unique	Peptide
1113	547.24	1092.47	1092.46	7.70	0	58	3.5e-006	1	U	K.DTDSEEEIR.E
2788	615.63	1843.88	1843.88	-1.47	1	64	2.3e-006	1	U	K.EAFSLFDKDGDGTITTK.E

36. Q3T1J1 **Mass:** 17049 **Score:** 121 **Matches:** 3(3) **Sequences:** 3(3) **emPAI:** 1.05
Eukaryotic translation initiation factor 5A-1 OS=Rattus norvegicus GN=Eif5a PE=1 SV=3

Query	Observed	Mr(expt)	Mr(calc)	ppm	Miss	Score	Expect	Rank	Unique	Peptide
108	388.74	775.46	775.46	-1.27	0	45	0.00012	1	U	K.NGFVVLK.G
219	414.73	827.44	827.44	0.84	0	26	0.014	1	U	R.LPEGDLGK.E
1761	649.88	1297.74	1297.74	-2.91	0	50	3.4e-005	1	U	K.VHLVGIDIFTGK.K

37. Q6IG03 **Mass:** 60977 **Score:** 120 **Matches:** 3(2) **Sequences:** 3(2) **emPAI:** 0.15
Keratin, type II cytoskeletal 73 OS=Rattus norvegicus GN=Krt73 PE=1 SV=1

Query	Observed	Mr(expt)	Mr(calc)	ppm	Miss	Score	Expect	Rank	Unique	Peptide
35	366.70	731.38	731.38	-2.08	0	22	0.12	1		R.LDSELR.G
792	503.25	1004.48	1004.46	16.9	0	25	0.025	1		K.LLEGEECR.M
2185	738.39	1474.77	1474.78	-4.24	0	73	3.1e-007	1		R.FLEQQNQVLQTK.W

38. Q6LED0 **Mass:** 15509 **Score:** 116 **Matches:** 3(3) **Sequences:** 3(3) **emPAI:** 1.20
Histone H3.1 OS=Rattus norvegicus PE=1 SV=3

Query	Observed	Mr(expt)	Mr(calc)	ppm	Miss	Score	Expect	Rank	Unique	Peptide
14	358.21	714.40	714.40	-0.27	0	26	0.02	1	U	K.DIQLAR.R
229	416.25	830.49	830.49	0.03	0	52	4.1e-005	1	U	K.STELLIR.K
896	516.80	1031.58	1031.59	-3.44	1	38	0.00081	1	U	R.YRPGTVALR.E

Proteins matching the same set of peptides:
P84245 **Mass:** 15376 **Score:** 116 **Matches:** 3(3) **Sequences:** 3(3)
Histone H3.3 OS=Rattus norvegicus GN=H3f3b PE=1 SV=2

39. Q27W02 **Mass:** 17210 **Score:** 111 **Matches:** 2(2) **Sequences:** 2(2) **emPAI:** 0.61
Protein mago nashi homolog OS=Rattus norvegicus GN=Magoh PE=2 SV=1

Query	Observed	Mr(expt)	Mr(calc)	ppm	Miss	Score	Expect	Rank	Unique	Peptide
1375	587.32	1172.63	1172.64	-5.39	0	61	1.4e-005	1	U	K.IGSLIDVNQSK.D
1724	644.35	1286.69	1286.69	-4.70	0	51	6.3e-005	1	U	R.VFYYLVQDLK.C

40. P35434 **Mass:** 17584 **Score:** 110 **Matches:** 2(2) **Sequences:** 2(2) **emPAI:** 0.59
ATP synthase subunit delta, mitochondrial OS=Rattus norvegicus GN=Atp5d PE=1 SV=2

Query	Observed	Mr(expt)	Mr(calc)	ppm	Miss	Score	Expect	Rank	Unique	Peptide
31	365.22	728.42	728.42	-2.19	0	43	0.00052	1	U	R.AEIQIR.I
1959	688.32	1374.63	1374.64	-3.86	0	67	7.6e-007	1	U	K.AQSELSGAADEAAR.A

41. Q9EQX9 **Mass:** 17170 **Score:** 106 **Matches:** 3(3) **Sequences:** 3(3) **emPAI:** 1.04
Ubiquitin-conjugating enzyme E2 N OS=Rattus norvegicus GN=Ube2n PE=1 SV=1

Query	Observed	Mr(expt)	Mr(calc)	ppm	Miss	Score	Expect	Rank	Unique	Peptide
405	437.75	873.49	873.50	-13.32	0	35	0.0039	1	U	R.ICLDILK.D
730	493.25	984.49	984.50	-14.36	0	26	0.013	1	U	K.IYHPNVDK.L
906	518.82	1035.63	1035.63	-1.36	0	46	5e-005	1	U	R.LLAEPVPGIK.A

42. P63039 **Mass:** 61088 **Score:** 98 **Matches:** 2(2) **Sequences:** 2(2) **emPAI:** 0.15
60 kDa heat shock protein, mitochondrial OS=Rattus norvegicus GN=Hspd1 PE=1 SV=1

Query	Observed	Mr(expt)	Mr(calc)	ppm	Miss	Score	Expect	Rank	Unique	Peptide
672	480.76	959.50	959.50	-3.79	0	56	2.5e-005	1	U	R.VTDALNATR.A
1570	617.30	1232.59	1232.59	-2.74	0	42	0.00039	1	U	K.VGGTSDVEVNEK.K

43. P00173 **Mass:** 15346 **Score:** 96 **Matches:** 1(1) **Sequences:** 1(1) **emPAI:** 0.30
Cytochrome b5 OS=Rattus norvegicus GN=Cyb5a PE=1 SV=2

Query	Observed	Mr(expt)	Mr(calc)	ppm	Miss	Score	Expect	Rank	Unique	Peptide
3101	735.98	2204.93	2204.92	2.03	0	96	3.2e-010	1	U	R.EQAGGDATENFEDVGHSTDAR.E

44. Q09073 **Mass:** 33108 **Score:** 92 **Matches:** 2(2) **Sequences:** 2(2) **emPAI:** 0.28
ADP/ATP translocase 2 OS=Rattus norvegicus GN=Slc25a5 PE=1 SV=3

Query	Observed	Mr(expt)	Mr(calc)	ppm	Miss	Score	Expect	Rank	Unique	Peptide
1264	568.84	1135.67	1135.67	-2.41	0	29	0.0022	1		K.LLLQVQHASK.Q
1533	610.34	1218.66	1218.66	-3.28	0	63	2.9e-006	1	U	K.DFLAGGVAAAISK.T

45. P62856 **Mass:** 13292 **Score:** 91 **Matches:** 1(1) **Sequences:** 1(1) **emPAI:** 0.36
40S ribosomal protein S26 OS=Rattus norvegicus GN=Rps26 PE=3 SV=3

Query	Observed	Mr(expt)	Mr(calc)	ppm	Miss	Score	Expect	Rank	Unique	Peptide
2515	827.42	1652.82	1652.83	-2.96	0	91	5.3e-009	1	U	R.DISEASVFDAYVLPK.L

46. Q5XFX0 **Mass:** 22550 **Score:** 90 **Matches:** 2(2) **Sequences:** 2(2) **emPAI:** 0.44
Transgelin-2 OS=Rattus norvegicus GN=Tagln2 PE=2 SV=1

Query	Observed	Mr(expt)	Mr(calc)	ppm	Miss	Score	Expect	Rank	Unique	Peptide
835	506.76	1011.51	1011.51	1.86	0	22	0.032	1	U	K.IQASTMAFK.Q
2569	847.91	1693.81	1693.81	-1.84	0	67	1e-006	1	U	K.QMEQISQFLQAAER.Y

47. P62890 **Mass:** 12947 **Score:** 89 **Matches:** 2(2) **Sequences:** 2(2) **emPAI:** 0.87
60S ribosomal protein L30 OS=Rattus norvegicus GN=Rpl30 PE=3 SV=2

Query	Observed	Mr(expt)	Mr(calc)	ppm	Miss	Score	Expect	Rank	Unique	Peptide
500	453.24	904.47	904.46	5.85	0	24	0.041	1	U	K.SLESINSR.L
1899	677.38	1352.74	1352.76	-14.71	0	66	2.4e-006	1	U	K.LVILANNCPALR.K

48. Q7M767 **Mass:** 16399 **Score:** 85 **Matches:** 2(1) **Sequences:** 2(1) **emPAI:** 0.28
Ubiquitin-conjugating enzyme E2 variant 2 OS=Rattus norvegicus GN=Ube2v2 PE=1 SV=3

Query	Observed	Mr(expt)	Mr(calc)	ppm	Miss	Score	Expect	Rank	Unique	Peptide
847	508.27	1014.52	1014.51	9.29	0	20	0.077	2	U	K.YPEAPPSVR.F
1431	594.31	1186.60	1186.61	-5.82	0	65	2.5e-006	1	U	R.LLEELEEGQK.G

49. Q5BJY9 **Mass:** 47732 **Score:** 85 **Matches:** 3(3) **Sequences:** 3(3) **emPAI:** 0.30
Keratin, type I cytoskeletal 18 OS=Rattus norvegicus GN=Krt18 PE=1 SV=3

Query	Observed	Mr(expt)	Mr(calc)	ppm	Miss	Score	Expect	Rank	Unique	Peptide
166	404.21	806.40	806.39	8.69	0	22	0.047	1		R.LAADDFR.V
171	405.22	808.43	808.43	0.20	0	39	0.0006	1		R.LASYLDK.V
1341	580.81	1159.60	1159.61	-5.14	0	25	0.026	1	U	R.DAETTLLELR.R

50. P47198 **Mass:** 14837 **Score:** 85 **Matches:** 2(2) **Sequences:** 2(2) **emPAI:** 0.73
60S ribosomal protein L22 OS=Rattus norvegicus GN=Rpl22 PE=2 SV=2

Query	Observed	Mr(expt)	Mr(calc)	ppm	Miss	Score	Expect	Rank	Unique	Peptide
140	398.69	795.37	795.38	-2.29	0	38	0.00076	1	U	K.ESYELR.Y
1608	621.84	1241.67	1241.67	-2.56	0	47	0.00013	1	U	K.AGNLGGGVVTIER.S

51. P62268 **Mass:** 15969 **Score:** 82 **Matches:** 2(2) **Sequences:** 2(2) **emPAI:** 0.67
40S ribosomal protein S23 OS=Rattus norvegicus GN=Rps23 PE=1 SV=3

Query	Observed	Mr(expt)	Mr(calc)	ppm	Miss	Score	Expect	Rank	Unique	Peptide
979	352.84	1055.50	1055.51	-10.07	0	55	2e-005	1	U	K.ANPFGGASHAK.G
1443	595.86	1189.70	1189.71	-4.89	0	27	0.0037	1	U	K.VANVSLLALYK.G

52. P15865 **Mass:** 21974 **Score:** 81 **Matches:** 1(1) **Sequences:** 1(1) **emPAI:** 0.21
Histone H1.4 OS=Rattus norvegicus GN=Hist1h1e PE=1 SV=3

Query	Observed	Mr(expt)	Mr(calc)	ppm	Miss	Score	Expect	Rank	Unique	Peptide
1474	599.84	1197.66	1197.66	1.77	0	81	4.2e-008	1	U	K.ASGPPVSELITK.A

53. P62963 **Mass:** 15119 **Score:** 77 **Matches:** 1(1) **Sequences:** 1(1) **emPAI:** 0.31
Profilin-1 OS=Rattus norvegicus GN=Pfn1 PE=1 SV=2

Query	Observed	Mr(expt)	Mr(calc)	ppm	Miss	Score	Expect	Rank	Unique	Peptide
2138	727.88	1453.75	1453.76	-1.40	0	77	1.4e-007	1	U	R.SSFFVNGLTLGGQK.C

54. P00406 **Mass:** 26096 **Score:** 74 **Matches:** 2(2) **Sequences:** 2(2) **emPAI:** 0.37
Cytochrome c oxidase subunit 2 OS=Rattus norvegicus GN=Mtco2 PE=2 SV=2

Query	Observed	Mr(expt)	Mr(calc)	ppm	Miss	Score	Expect	Rank	Unique	Peptide
30	365.20	728.38	728.38	-3.09	0	34	0.0023	1	U	K.TDAIPGR.L
1417	591.85	1181.68	1181.68	-3.03	0	41	0.00026	1	U	R.VVLPMELPIR.M

55. Q4QQV3 **Mass:** 18044 **Score:** 71 **Matches:** 1(1) **Sequences:** 1(1) **emPAI:** 0.25
Protein FAM162A OS=Rattus norvegicus GN=Fam162a PE=2 SV=1

Query	Observed	Mr(expt)	Mr(calc)	ppm	Miss	Score	Expect	Rank	Unique	Peptide
2139	485.59	1453.76	1453.76	-0.80	1	71	4.7e-007	1	U	K.RHESLTSLNLER.K

56. P60868 **Mass:** 13478 **Score:** 59 **Matches:** 1(1) **Sequences:** 1(1) **emPAI:** 0.35
40S ribosomal protein S20 OS=Rattus norvegicus GN=Rps20 PE=3 SV=1

Query	Observed	Mr(expt)	Mr(calc)	ppm	Miss	Score	Expect	Rank	Unique	Peptide
1896	450.93	1349.75	1349.76	-0.82	0	59	3.7e-006	1	U	R.LIDLHSPSEIVK.Q

57. P04644 **Mass:** 15557 **Score:** 58 **Matches:** 1(1) **Sequences:** 1(1) **emPAI:** 0.30
40S ribosomal protein S17 OS=Rattus norvegicus GN=Rps17 PE=1 SV=3

Query	Observed	Mr(expt)	Mr(calc)	ppm	Miss	Score	Expect	Rank	Unique	Peptide
1654	629.84	1257.66	1257.66	-1.96	0	58	1.2e-005	1	U	R.VCEEIAIIPSK.N

58. P61354 **Mass:** 15788 **Score:** 56 **Matches:** 1(1) **Sequences:** 1(1) **emPAI:** 0.29
60S ribosomal protein L27 OS=Rattus norvegicus GN=Rpl27 PE=2 SV=2

Query	Observed	Mr(expt)	Mr(calc)	ppm	Miss	Score	Expect	Rank	Unique	Peptide
213	413.78	825.54	825.54	-1.04	0	56	4.1e-006	1	U	K.VVLVLAGR.Y

59. Q3KRE8 **Mass:** 50377 **Score:** 55 **Matches:** 2(2) **Sequences:** 2(2) **emPAI:** 0.18
Tubulin beta-2B chain OS=Rattus norvegicus GN=Tubb2b PE=1 SV=1

Query	Observed	Mr(expt)	Mr(calc)	ppm	Miss	Score	Expect	Rank	Unique	Peptide
1053	359.85	1076.52	1076.53	-1.86	1	34	0.0032	1		K.IREEYPDR.I
2762	608.31	1821.91	1821.92	-4.79	0	22	0.04	1	U	R.EIVHIQAGQCGNQIGAK.F

Proteins matching the same set of peptides:
P69897 **Mass:** 50095 **Score:** 55 **Matches:** 2(2) **Sequences:** 2(2)
Tubulin beta-5 chain OS=Rattus norvegicus GN=Tubb5 PE=1 SV=1
P85108 **Mass:** 50274 **Score:** 55 **Matches:** 2(2) **Sequences:** 2(2)
Tubulin beta-2A chain OS=Rattus norvegicus GN=Tubb2a PE=1 SV=1

60. Q6P9T8 **Mass:** 50225 **Score:** 55 **Matches:** 2(2) **Sequences:** 2(2) **emPAI:** 0.18
Tubulin beta-4B chain OS=Rattus norvegicus GN=Tubb4b PE=1 SV=1

Query	Observed	Mr(expt)	Mr(calc)	ppm	Miss	Score	Expect	Rank	Unique	Peptide
1053	359.85	1076.52	1076.53	-1.86	1	34	0.0032	1		K.IREEYPDR.I
2762	608.31	1821.91	1821.92	-4.79	0	22	0.04	1	U	R.EIVHLQAGQCGNQIGAK.F

61. Q05962 **Mass:** 33196 **Score:** 51 **Matches:** 2(2) **Sequences:** 2(2) **emPAI:** 0.28
ADP/ATP translocase 1 OS=Rattus norvegicus GN=Slc25a4 PE=1 SV=3

Query	Observed	Mr(expt)	Mr(calc)	ppm	Miss	Score	Expect	Rank	Unique	Peptide
1264	568.84	1135.67	1135.67	-2.41	0	29	0.0022	1		K.LLLQVQHASK.Q
1533	610.34	1218.66	1218.66	-3.28	0	23	0.034	2	U	K.DFLAGGIAAAVSK.T

62. P63324 **Mass:** 14858 **Score:** 50 **Matches:** 1(1) **Sequences:** 1(1) **emPAI:** 0.31
40S ribosomal protein S12 OS=Rattus norvegicus GN=Rps12 PE=1 SV=2

Query	Observed	Mr(expt)	Mr(calc)	ppm	Miss	Score	Expect	Rank	Unique	Peptide
1027	356.21	1065.59	1065.59	0.31	0	50	4.8e-005	1	U	K.TALIHDGLAR.G

63. Q4KLF8 **Mass:** 16367 **Score:** 46 **Matches:** 1(1) **Sequences:** 1(1) **emPAI:** 0.28
Actin-related protein 2/3 complex subunit 5 OS=Rattus norvegicus GN=Arpc5 PE=1 SV=3

Query	Observed	Mr(expt)	Mr(calc)	ppm	Miss	Score	Expect	Rank	Unique	Peptide
1035	535.81	1069.61	1069.62	-10.98	0	46	8.3e-005	1	U	K.ALAAGGVGSIVR.V

64. B0BNA5 **Mass:** 16036 **Score:** 46 **Matches:** 1(1) **Sequences:** 1(1) **emPAI:** 0.29
Coactosin-like protein OS=Rattus norvegicus GN=Cotl1 PE=1 SV=1

Query	Observed	Mr(expt)	Mr(calc)	ppm	Miss	Score	Expect	Rank	Unique	Peptide
960	525.76	1049.50	1049.50	0.47	0	46	0.00019	1	U	K.ELEEDFIR.S

65. P62278 **Mass:** 17212 **Score:** 45 **Matches:** 1(1) **Sequences:** 1(1) **emPAI:** 0.27
40S ribosomal protein S13 OS=Rattus norvegicus GN=Rps13 PE=1 SV=2

Query	Observed	Mr(expt)	Mr(calc)	ppm	Miss	Score	Expect	Rank	Unique	Peptide
1647	627.38	1252.75	1252.75	-3.33	0	45	3.1e-005	1	U	K.GLTPSQIGVILR.D

66. P62982 **Mass:** 18282 **Score:** 44 **Matches:** 1(1) **Sequences:** 1(1) **emPAI:** 0.25
Ubiquitin-40S ribosomal protein S27a OS=Rattus norvegicus GN=Rps27a PE=1 SV=2

Query	Observed	Mr(expt)	Mr(calc)	ppm	Miss	Score	Expect	Rank	Unique	Peptide
1030	534.31	1066.61	1066.61	-0.48	0	44	0.00011	1	U	K.ESTLHLVLR.L

Proteins matching the same set of peptides:
P62986 **Mass:** 15004 **Score:** 44 **Matches:** 1(1) **Sequences:** 1(1)
Ubiquitin-60S ribosomal protein L40 OS=Rattus norvegicus GN=Uba52 PE=1 SV=2
P0CG51 **Mass:** 34348 **Score:** 43 **Matches:** 1(1) **Sequences:** 1(1)
Polyubiquitin-B OS=Rattus norvegicus GN=Ubb PE=1 SV=1
Q63429 **Mass:** 91145 **Score:** 42 **Matches:** 1(1) **Sequences:** 1(1)
Polyubiquitin-C OS=Rattus norvegicus GN=Ubc PE=1 SV=1

67. P11250 **Mass:** 13613 **Score:** 44 **Matches:** 1(1) **Sequences:** 1(1) **emPAI:** 0.35
60S ribosomal protein L34 OS=Rattus norvegicus GN=Rpl34 PE=1 SV=3

Query	Observed	Mr(expt)	Mr(calc)	ppm	Miss	Score	Expect	Rank	Unique	Peptide
763	499.25	996.48	996.49	-4.46	0	44	0.00028	1	U	R.LSYNTASNK.T

68. B0K025 **Mass:** 16918 **Score:** 43 **Matches:** 1(1) **Sequences:** 1(1) **emPAI:** 0.27
Oligosaccharyltransferase complex subunit OSTC OS=Rattus norvegicus GN=Ostc PE=2 SV=1

Query	Observed	Mr(expt)	Mr(calc)	ppm	Miss	Score	Expect	Rank	Unique	Peptide
2085	714.90	1427.78	1427.78	-3.45	0	43	0.00026	1	U	R.VPFLVLECPNLK.L

69. Q63495 **Mass:** 42979 **Score:** 40 **Matches:** 1(1) **Sequences:** 1(1) **emPAI:** 0.10
Advanced glycosylation end product-specific receptor OS=Rattus norvegicus GN=Ager PE=1 SV=1

Query	Observed	Mr(expt)	Mr(calc)	ppm	Miss	Score	Expect	Rank	Unique	Peptide
2292	763.88	1525.75	1525.75	-0.81	0	40	0.00071	1	U	K.VLSPQGDPWDSVAR.I

70. P37397 **Mass:** 36583 **Score:** 39 **Matches:** 1(1) **Sequences:** 1(1) **emPAI:** 0.12
Calponin-3 OS=Rattus norvegicus GN=Cnn3 PE=1 SV=1

Query	Observed	Mr(expt)	Mr(calc)	ppm	Miss	Score	Expect	Rank	Unique	Peptide
1725	644.35	1286.69	1286.69	-2.29	0	39	0.00095	1	U	K.DGIILCELINK.L

71. P02773 **Mass:** 70166 **Score:** 39 **Matches:** 1(1) **Sequences:** 1(1) **emPAI:** 0.06
Alpha-fetoprotein OS=Rattus norvegicus GN=Afp PE=2 SV=1

Query	Observed	Mr(expt)	Mr(calc)	ppm	Miss	Score	Expect	Rank	Unique	Peptide
337	424.25	846.49	846.50	-8.81	1	39	0.00066	1	U	K.LSQKFPK.A

72. P35213 **Mass:** 28151 **Score:** 38 **Matches:** 1(1) **Sequences:** 1(1) **emPAI:** 0.16
14-3-3 protein beta/alpha OS=Rattus norvegicus GN=Ywhab PE=1 SV=3

Query	Observed	Mr(expt)	Mr(calc)	ppm	Miss	Score	Expect	Rank	Unique	Peptide
507	454.27	906.52	906.52	0.89	0	38	0.00081	1	U	R.NLLSVAYK.N

Proteins matching the same set of peptides:
P61983 **Mass:** 28456 **Score:** 38 **Matches:** 1(1) **Sequences:** 1(1)
14-3-3 protein gamma OS=Rattus norvegicus GN=Ywhag PE=1 SV=2
P62260 **Mass:** 29326 **Score:** 38 **Matches:** 1(1) **Sequences:** 1(1)
14-3-3 protein epsilon OS=Rattus norvegicus GN=Ywhae PE=1 SV=1
P63102 **Mass:** 27925 **Score:** 38 **Matches:** 1(1) **Sequences:** 1(1)
14-3-3 protein zeta/delta OS=Rattus norvegicus GN=Ywhaz PE=1 SV=1
P68255 **Mass:** 28046 **Score:** 38 **Matches:** 1(1) **Sequences:** 1(1)
14-3-3 protein theta OS=Rattus norvegicus GN=Ywhaq PE=1 SV=1
P68511 **Mass:** 28365 **Score:** 38 **Matches:** 1(1) **Sequences:** 1(1)
14-3-3 protein eta OS=Rattus norvegicus GN=Ywhah PE=1 SV=2

73. P18598 **Mass:** 34181 **Score:** 37 **Matches:** 1(1) **Sequences:** 1(1) **emPAI:** 0.13
Potassium-transporting ATPase subunit beta OS=Rattus norvegicus GN=Atp4b PE=2 SV=3

Query	Observed	Mr(expt)	Mr(calc)	ppm	Miss	Score	Expect	Rank	Unique	Peptide
1046	537.80	1073.59	1073.59	-1.74	0	37	0.0015	1	U	K.NTQVLIVCK.I

74. Q8K1Q4 **Score:** 34 **Matches:** 1(1) **Sequences:** 1(1) **emPAI:** 0.05
Leucine zipper putative tumor suppressor 3 OS=Rattus norvegicus GN=Lzts3 PE=1 SV=2

Query	Observed	Mr(expt)	Mr(calc)	ppm	Miss	Score	Expect	Rank	Unique	Peptide
769	500.79	999.56	999.53	22.2	0	34	0.0028	2	U	R.LQAELAAER.R

75. Q4KLH6 **Mass:** 161131 **Score:** 34 **Matches:** 1(1) **Sequences:** 1(1) **emPAI:** 0.03
Centrosomal protein of 162 kDa OS=Rattus norvegicus GN=Cep162 PE=1 SV=2

Query	Observed	Mr(expt)	Mr(calc)	ppm	Miss	Score	Expect	Rank	Unique	Peptide
1287	571.86	1141.71	1141.71	-1.68	1	34	0.00056	1	U	R.KIATQEVLLK.H

76. P15999 **Mass:** 59831 **Score:** 34 **Matches:** 1(1) **Sequences:** 1(1) **emPAI:** 0.07
ATP synthase subunit alpha, mitochondrial OS=Rattus norvegicus GN=Atp5a1 PE=1 SV=2

Query	Observed	Mr(expt)	Mr(calc)	ppm	Miss	Score	Expect	Rank	Unique	Peptide
1372	586.32	1170.63	1170.62	5.09	0	34	0.0027	1	U	R.VVDALGNAIDGK.G

77. P39032 **Mass:** 12317 **Score:** 33 **Matches:** 1(1) **Sequences:** 1(1) **emPAI:** 0.39
60S ribosomal protein L36 OS=Rattus norvegicus GN=Rpl36 PE=1 SV=2

Query	Observed	Mr(expt)	Mr(calc)	ppm	Miss	Score	Expect	Rank	Unique	Peptide
1630	624.81	1247.61	1247.62	-4.65	0	33	0.0046	1	U	R.EELSNVLAAMR.K

78. Q29TV8 **Mass:** 20641 **Score:** 31 **Matches:** 1(1) **Sequences:** 1(1) **emPAI:** 0.22
Gastrokine-2 OS=Rattus norvegicus GN=Gkn2 PE=2 SV=1

Query	Observed	Mr(expt)	Mr(calc)	ppm	Miss	Score	Expect	Rank	Unique	Peptide
2455	537.29	1608.83	1608.86	-17.40	1	31	0.0042	1	U	K.HIPLYEGEVATKPR.E

79. Q6AYZ1 **Mass:** 50590 **Score:** 30 **Matches:** 1(1) **Sequences:** 1(1) **emPAI:** 0.09
Tubulin alpha-1C chain OS=Rattus norvegicus GN=Tuba1c PE=1 SV=1

Query	Observed	Mr(expt)	Mr(calc)	ppm	Miss	Score	Expect	Rank	Unique	Peptide
848	508.29	1014.57	1014.57	-1.53	0	30	0.0085	1	U	K.DVNAAIATIK.T

Proteins matching the same set of peptides:
Q6P9V9 **Mass:** 50804 **Score:** 30 **Matches:** 1(1) **Sequences:** 1(1)
Tubulin alpha-1B chain OS=Rattus norvegicus GN=Tuba1b PE=1 SV=1
Q68FR8 **Mass:** 50612 **Score:** 30 **Matches:** 1(1) **Sequences:** 1(1)
Tubulin alpha-3 chain OS=Rattus norvegicus GN=Tuba3a PE=2 SV=1
P68370 **Mass:** 50788 **Score:** 30 **Matches:** 1(1) **Sequences:** 1(1)
Tubulin alpha-1A chain OS=Rattus norvegicus GN=Tuba1a PE=1 SV=1

80. P17078 **Mass:** 14544 **Score:** 28 **Matches:** 1(1) **Sequences:** 1(1) **emPAI:** 0.32
60S ribosomal protein L35 OS=Rattus norvegicus GN=Rpl35 PE=1 SV=3

Query	Observed	Mr(expt)	Mr(calc)	ppm	Miss	Score	Expect	Rank	Unique	Peptide
1293	572.34	1142.66	1142.67	-5.68	0	28	0.0059	1	U	R.VLTVINQTQK.E

81. O70454 **Mass:** 17559 **Score:** 28 **Matches:** 1(1) **Sequences:** 1(1) **emPAI:** 0.26
Protein BUD31 homolog OS=Rattus norvegicus GN=Bud31 PE=2 SV=2

Query	Observed	Mr(expt)	Mr(calc)	ppm	Miss	Score	Expect	Rank	Unique	Peptide
1771	435.86	1304.55	1304.54	3.42	0	28	0.0026	1	U	R.IIECTHCGCR.G

82. P13383 **Mass:** 77158 **Score:** 28 **Matches:** 1(1) **Sequences:** 1(1) **emPAI:** 0.06
Nucleolin OS=Rattus norvegicus GN=Ncl PE=1 SV=3

Query	Observed	Mr(expt)	Mr(calc)	ppm	Miss	Score	Expect	Rank	Unique	Peptide
483	450.27	898.52	898.52	-0.80	1	28	0.0085	1	U	K.GAVTPAKGAK.N

83. P00762 **Mass:** 26627 **Score:** 27 **Matches:** 1(1) **Sequences:** 1(1) **emPAI:** 0.17
Anionic trypsin-1 OS=Rattus norvegicus GN=Prss1 PE=1 SV=1

Query	Observed	Mr(expt)	Mr(calc)	ppm	Miss	Score	Expect	Rank	Unique	Peptide
3103	1106.05	2210.09	2210.10	-4.13	0	27	0.01	1	U	R.LGEHNINVLEGDEQFINAAK.I

84. P83883 **Mass:** 12718 **Score:** 27 **Matches:** 1(1) **Sequences:** 1(1) **emPAI:** 0.37
60S ribosomal protein L36a OS=Rattus norvegicus GN=Rpl36a PE=1 SV=2

Query	Observed	Mr(expt)	Mr(calc)	ppm	Miss	Score	Expect	Rank	Unique	Peptide
194	410.24	818.46	818.47	-1.70	1	27	0.013	1	U	R.KGQVIQF.-

85. O88761 **Mass:** 106707 **Score:** 27 **Matches:** 2(2) **Sequences:** 1(1) **emPAI:** 0.08
26S proteasome non-ATPase regulatory subunit 1 OS=Rattus norvegicus GN=Psmd1 PE=2 SV=1

Query	Observed	Mr(expt)	Mr(calc)	ppm	Miss	Score	Expect	Rank	Unique	Peptide
1381	588.32	1174.63	1174.60	22.1	0	27	0.017	1	U	R.NNNTDLMILK.N
1448	596.32	1190.62	1190.60	17.1	0	(23)	0.049	1	U	R.NNNTDLMILK.N

86. P52632 **Mass:** 90680 **Score:** 27 **Matches:** 1(1) **Sequences:** 1(1) **emPAI:** 0.05
Signal transducer and activator of transcription 5B OS=Rattus norvegicus GN=Stat5b PE=2 SV=1

Query	Observed	Mr(expt)	Mr(calc)	ppm	Miss	Score	Expect	Rank	Unique	Peptide
55	372.24	742.46	742.47	-9.91	0	27	0.019	1	U	K.TLQLLR.K

Proteins matching the same set of peptides:
Q62771 **Mass:** 91290 **Score:** 27 **Matches:** 1(1) **Sequences:** 1(1)
Signal transducer and activator of transcription 5A OS=Rattus norvegicus GN=Stat5a PE=1 SV=1

87. P08426 **Mass:** 26936 **Score:** 26 **Matches:** 1(1) **Sequences:** 1(1) **emPAI:** 0.16
Cationic trypsin-3 OS=Rattus norvegicus GN=Try3 PE=2 SV=1

Query	Observed	Mr(expt)	Mr(calc)	ppm	Miss	Score	Expect	Rank	Unique	Peptide
369	429.75	857.49	857.50	-2.43	0	26	0.025	1	U	R.VSTVSLPR.S

88. D4AE41 **Mass:** 42281 **Score:** 26 **Matches:** 1(1) **Sequences:** 1(1) **emPAI:** 0.10
RNA binding motif protein, X-linked-like-1 OS=Rattus norvegicus GN=Rbmxl1 PE=3 SV=1

Query	Observed	Mr(expt)	Mr(calc)	ppm	Miss	Score	Expect	Rank	Unique	Peptide
2098	718.37	1434.72	1434.74	-9.34	0	27	0.014	1	U	K.LFIGGLNTETNEK.A

Proteins matching the same set of peptides:
Q4V898 **Mass:** 42232 **Score:** 26 **Matches:** 1(1) **Sequences:** 1(1)
RNA-binding motif protein, X chromosome OS=Rattus norvegicus GN=Rbmx PE=1 SV=1
P84586 **Mass:** 42178 **Score:** 26 **Matches:** 1(1) **Sequences:** 1(1)
RNA-binding motif protein, X chromosome retrogene-like OS=Rattus norvegicus GN=Rbmxrtl PE=3 SV=1

89. P26769 **Score:** 26 **Matches:** 1(1) **Sequences:** 1(1) **emPAI:** 0.03
Adenylate cyclase type 2 OS=Rattus norvegicus GN=Adcy2 PE=1 SV=1

Query	Observed	Mr(expt)	Mr(calc)	ppm	Miss	Score	Expect	Rank	Unique	Peptide

31	365.22	728.42	728.42	-2.19	0	26	0.026	2	U	K.AEIIQR.L

90. Q88778 **Score:** 26 **Matches:** 1(1) **Sequences:** 1(1) **emPAI:** 0.01
Protein bassoon OS=Rattus norvegicus GN=Bsn PE=1 SV=3

Query	Observed	Mr(expt)	Mr(calc)	ppm	Miss	Score	Expect	Rank	Unique	Peptide
31	365.22	728.42	728.42	-2.19	0	26	0.026	2	U	K.AELLQR.Q

91. P0C5H9 **Mass:** 20831 **Score:** 26 **Matches:** 1(1) **Sequences:** 1(1) **emPAI:** 0.22
Mesencephalic astrocyte-derived neurotrophic factor OS=Rattus norvegicus GN=Manf PE=1 SV=1

Query	Observed	Mr(expt)	Mr(calc)	ppm	Miss	Score	Expect	Rank	Unique	Peptide
2565	**846.43**	**1690.85**	**1690.87**	**-11.53**	**0**	**26**	**0.019**	**1**	**U**	**R.DVTFSPATIEEELIK.F**

92. Q5M7V8 **Mass:** 108302 **Score:** 25 **Matches:** 1(1) **Sequences:** 1(1) **emPAI:** 0.04
Thyroid hormone receptor-associated protein 3 OS=Rattus norvegicus GN=Thrap3 PE=1 SV=1

Query	Observed	Mr(expt)	Mr(calc)	ppm	Miss	Score	Expect	Rank	Unique	Peptide
40	**367.70**	**733.39**	**733.40**	**-3.67**	**1**	**25**	**0.023**	**1**	**U**	**R.KSSTSPK.W**

93. Q9EPY0 **Score:** 25 **Matches:** 1(1) **Sequences:** 1(1) **emPAI:** 0.07
Caspase recruitment domain-containing protein 9 OS=Rattus norvegicus GN=Card9 PE=1 SV=1

Query	Observed	Mr(expt)	Mr(calc)	ppm	Miss	Score	Expect	Rank	Unique	Peptide
14	358.21	714.40	714.40	-0.27	0	25	0.027	2	U	R.DLLQAR.V

94. P47819 **Mass:** 49984 **Score:** 25 **Matches:** 1(1) **Sequences:** 1(1) **emPAI:** 0.09
Glial fibrillary acidic protein OS=Rattus norvegicus GN=Gfap PE=1 SV=2

Query	Observed	Mr(expt)	Mr(calc)	ppm	Miss	Score	Expect	Rank	Unique	Peptide
78	**380.21**	**758.41**	**758.43**	**-21.55**	**1**	**25**	**0.047**	**1**	**U**	**R.VDLERK.V**

95. Q9Z137 **Mass:** 37368 **Score:** 25 **Matches:** 1(1) **Sequences:** 1(1) **emPAI:** 0.12
Galactosylgalactosylxylosylprotein 3-beta-glucuronosyltransferase 2 OS=Rattus norvegicus GN=B3gat2 PE=1 SV=1

Query	Observed	Mr(expt)	Mr(calc)	ppm	Miss	Score	Expect	Rank	Unique	Peptide
203	**412.75**	**823.49**	**823.48**	**10.8**	**1**	**25**	**0.006**	**1**	**U**	**K.RPWLPR.A**

96. P29117 **Mass:** 22138 **Score:** 24 **Matches:** 1(1) **Sequences:** 1(1) **emPAI:** 0.20
Peptidyl-prolyl cis-trans isomerase F, mitochondrial OS=Rattus norvegicus GN=Ppif PE=1 SV=2

Query	Observed	Mr(expt)	Mr(calc)	ppm	Miss	Score	Expect	Rank	Unique	Peptide
4	**350.73**	**699.45**	**699.45**	**-1.80**	**0**	**24**	**0.015**	**1**	**U**	**R.VVLELK.A**

97. Q9JLA3 **Score:** 24 **Matches:** 1(1) **Sequences:** 1(1) **emPAI:** 0.02
UDP-glucose:glycoprotein glucosyltransferase 1 OS=Rattus norvegicus GN=Uggt1 PE=1 SV=2

Query	Observed	Mr(expt)	Mr(calc)	ppm	Miss	Score	Expect	Rank	Unique	Peptide
117	390.22	778.42	778.44	-24.61	0	24	0.033	2	U	R.IIWGYK.I

98. Q05030 **Score:** 24 **Matches:** 1(1) **Sequences:** 1(1) **emPAI:** 0.03
Platelet-derived growth factor receptor beta OS=Rattus norvegicus GN=Pdgfrb PE=2 SV=2

Query	Observed	Mr(expt)	Mr(calc)	ppm	Miss	Score	Expect	Rank	Unique	Peptide
117	390.22	778.42	778.42	-5.01	0	24	0.033	2	U	R.LLGEGYK.K

99. Q9JKS6 **Mass:** 554201 **Score:** 24 **Matches:** 2(2) **Sequences:** 1(1) **emPAI:** 0.01
Protein piccolo OS=Rattus norvegicus GN=Pclo PE=1 SV=1

Query	Observed	Mr(expt)	Mr(calc)	ppm	Miss	Score	Expect	Rank	Unique	Peptide
844	**507.81**	**1013.61**	**1013.61**	**-1.37**	**1**	**24**	**0.018**	**1**	**U**	**R.VDAKVEIIK.H** 843

100. P86411 **Mass:** 212595 **Score:** 24 **Matches:** 1(1) **Sequences:** 1(1) **emPAI:** 0.02
Ral GTPase-activating protein subunit alpha-2 OS=Rattus norvegicus GN=Ralgapa2 PE=1 SV=1

Query	Observed	Mr(expt)	Mr(calc)	ppm	Miss	Score	Expect	Rank	Unique	Peptide
888	**515.26**	**1028.50**	**1028.52**	**-13.67**	**0**	**24**	**0.027**	**1**	**U**	**R.MFASWLFK.A**

101. P67779 **Mass:** 29859 **Score:** 23 **Matches:** 1(1) **Sequences:** 1(1) **emPAI:** 0.15
Prohibitin OS=Rattus norvegicus GN=Phb PE=1 SV=1

Query	Observed	Mr(expt)	Mr(calc)	ppm	Miss	Score	Expect	Rank	Unique	Peptide
1517	**607.38**	**1212.74**	**1212.73**	**5.18**	**0**	**23**	**0.0061**	**1**	**U**	**R.VLPSITTEILK.S**

102. P61078 **Mass:** 16904 **Score:** 23 **Matches:** 1(1) **Sequences:** 1(1) **emPAI:** 0.27
Ubiquitin-conjugating enzyme E2 D3 OS=Rattus norvegicus GN=Ube2d3 PE=2 SV=1

Query	Observed	Mr(expt)	Mr(calc)	ppm	Miss	Score	Expect	Rank	Unique	Peptide
1526	**609.33**	**1216.65**	**1216.65**	**-0.05**	**0**	**23**	**0.036**	**1**	**U**	**R.SQWSPALTISK.V**

Proteins matching the same set of peptides:
P62839 **Mass:** 16953 **Score:** 23 **Matches:** 1(1) **Sequences:** 1(1)
Ubiquitin-conjugating enzyme E2 D2 OS=Rattus norvegicus GN=Ube2d2 PE=1 SV=1
P70711 **Mass:** 16819 **Score:** 23 **Matches:** 1(1) **Sequences:** 1(1)
Ubiquitin-conjugating enzyme E2 D2B OS=Rattus norvegicus GN=Ube2d2b PE=1 SV=1

103. Q9QX74 **Score:** 23 **Matches:** 1(1) **Sequences:** 1(1) **emPAI:** 0.03
SH3 and multiple ankyrin repeat domains protein 2 OS=Rattus norvegicus GN=Shank2 PE=1 SV=2

Query	Observed	Mr(expt)	Mr(calc)	ppm	Miss	Score	Expect	Rank	Unique	Peptide
747	495.77	989.52	989.53	-16.38	1	23	0.051	2	U	R.MNIERALK.Q

104. Q6QLM7 **Mass:** 117642 **Score:** 23 **Matches:** 1(1) **Sequences:** 1(1) **emPAI:** 0.04
Kinesin heavy chain isoform 5A OS=Rattus norvegicus GN=Kif5a PE=1 SV=1

Query	Observed	Mr(expt)	Mr(calc)	ppm	Miss	Score	Expect	Rank	Unique	Peptide
18	359.71	717.40	717.39	18.9	0	23	0.048	1	U	K.TIDELK.D

105. P36372 **Score:** 23 **Matches:** 1(1) **Sequences:** 1(1) **emPAI:** 0.05
Antigen peptide transporter 2 OS=Rattus norvegicus GN=Tap2 PE=2 SV=1

Query	Observed	Mr(expt)	Mr(calc)	ppm	Miss	Score	Expect	Rank	Unique	Peptide
605	472.77	943.52	943.51	9.74	1	23	0.053	2	U	R.QLWWRR.D

106. P62870 **Mass:** 13219 **Score:** 22 **Matches:** 1(0) **Sequences:** 1(0)
Transcription elongation factor B polypeptide 2 OS=Rattus norvegicus GN=Tceb2 PE=1 SV=1

Query	Observed	Mr(expt)	Mr(calc)	ppm	Miss	Score	Expect	Rank	Unique	Peptide
914	520.27	1038.52	1038.52	0.34	0	22	0.11	1	U	K.ESSTVFELK.R

107. O54939 **Mass:** 34657 **Score:** 22 **Matches:** 1(1) **Sequences:** 1(1) **emPAI:** 0.13
Testosterone 17-beta-dehydrogenase 3 OS=Rattus norvegicus GN=Hsd17b3 PE=2 SV=1

Query	Observed	Mr(expt)	Mr(calc)	ppm	Miss	Score	Expect	Rank	Unique	Peptide
1521	608.33	1214.64	1214.65	-6.26	0	22	0.055	1	U	K.LQVISEEIER.T

108. Q80T22 **Mass:** 52141 **Score:** 21 **Matches:** 1(1) **Sequences:** 1(1) **emPAI:** 0.08
Sodium-dependent glucose transporter 1 OS=Rattus norvegicus GN=Naglt1 PE=2 SV=1

Query	Observed	Mr(expt)	Mr(calc)	ppm	Miss	Score	Expect	Rank	Unique	Peptide
422	442.76	883.51	883.51	-1.47	0	21	0.026	1	U	K.VATLPLDR.K

109. Q07066 **Mass:** 22562 **Score:** 21 **Matches:** 1(0) **Sequences:** 1(0)
Peroxisomal membrane protein 2 OS=Rattus norvegicus GN=Pxmp2 PE=1 SV=2

Query	Observed	Mr(expt)	Mr(calc)	ppm	Miss	Score	Expect	Rank	Unique	Peptide
701	487.28	972.55	972.56	-13.19	0	21	0.069	1	U	R.SLEVSGLLR.Y

110. P0C0R5 **Score:** 21 **Matches:** 1(0) **Sequences:** 1(0)
Phosphoinositide 3-kinase regulatory subunit 4 OS=Rattus norvegicus GN=Pik3r4 PE=3 SV=2

Query	Observed	Mr(expt)	Mr(calc)	ppm	Miss	Score	Expect	Rank	Unique	Peptide
475	449.74	897.47	897.45	23.5	0	21	0.061	2	U	R.LCVFFGR.Q

111. Q6AXQ7 **Score:** 21 **Matches:** 1(0) **Sequences:** 1(0)
IQ and AAA domain-containing protein 1-like OS=Rattus norvegicus GN=Iqca1p1 PE=2 SV=2

Query	Observed	Mr(expt)	Mr(calc)	ppm	Miss	Score	Expect	Rank	Unique	Peptide
7	354.71	707.40	707.40	-5.95	0	21	0.057	1	U	R.YVLWK.R

112. P22791 **Score:** 20 **Matches:** 1(0) **Sequences:** 1(0)
Hydroxymethylglutaryl-CoA synthase, mitochondrial OS=Rattus norvegicus GN=Hmgcs2 PE=2 SV=1

Query	Observed	Mr(expt)	Mr(calc)	ppm	Miss	Score	Expect	Rank	Unique	Peptide
605	472.77	943.52	943.52	-0.74	1	20	0.092	3	U	R.KIQNQWK.Q

Mascot: http://www.matrixscience.com/

Supplementary Figure 1

Figure 1c

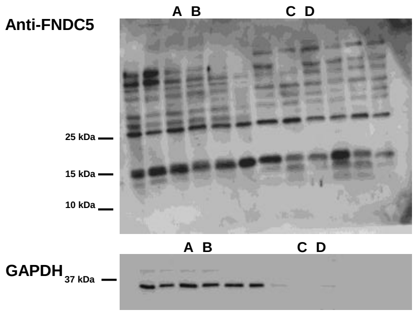

Figure 1d

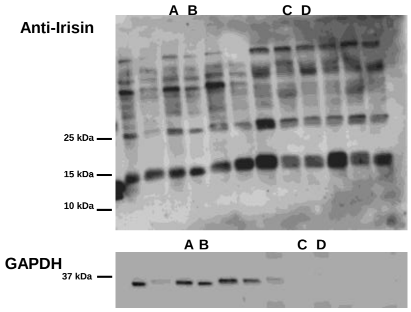

A and B: gastric mucosa

C and D: gastric secretoma

Figure 2a

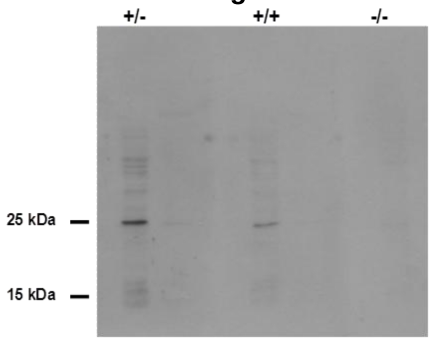

(+/-) FNDC5 antibody not preincubated with the immunogen, (+/+) FNDC5 antibody preincubated with FNDC5 recombinant protein, (-/-) negative control without primary antibody.

Figure 5a (mono Ab)

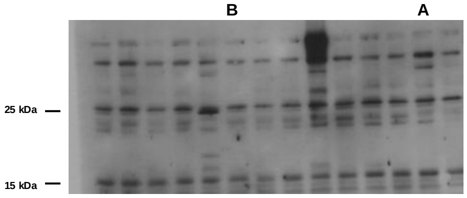

A: C (control)

B: HF (high fat diet)

Figure 6a BAT

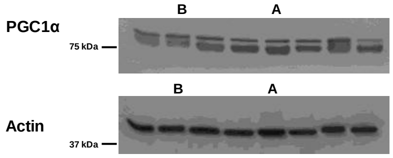

Figure 6a mucosa

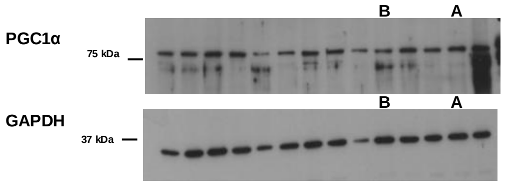

A: C (control)

B: HF (high fat diet)

Full blots for the representative images in figure 1c, 1d, 2a, 5a and 6a that were cropped in the submitted figures.

Supplementary figure 2

(a)

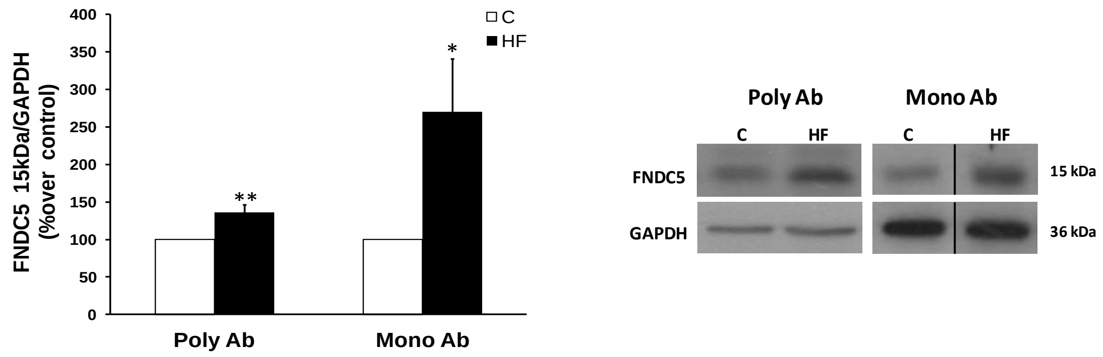

(b)

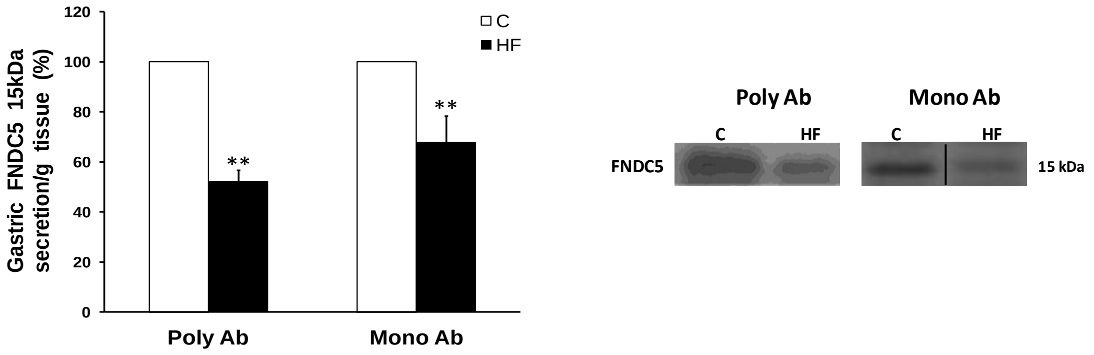

Levels of expression of 15 kDa band. (a)15 kDa band levels in gastric mucosa and (b) in gastric secretome and representative blot images detected by western blot with two FNDC5 antibodies (Poly Ab, polyclonal antibody; Mono Ab, monoclonal antibody). GAPDH was used to normalize the protein levels. Values are expressed as the mean SEM. N=6-8. Error bars indicate the SEM. *P<0.05, **P<0.01 vs. Control. C, control; HF, high fat diet. Dividing lines indicate splicing of the same gel.
